# Structure of co-expression networks of *Bifidobacterium* species in response to human milk oligosaccharides

**DOI:** 10.3389/fmolb.2023.1040721

**Published:** 2023-01-26

**Authors:** Kevin J. González-Morelo, Edgardo Galán-Vásquez, Felipe Melis, Ernesto Pérez-Rueda, Daniel Garrido

**Affiliations:** ^1^ Department of Chemical and Bioprocess Engineering, School of Engineering, Pontificia Universidad Católica de Chile, Santiago, Chile; ^2^ Departamento de Ingeniería de Sistemas Computacionales y Automatización, Instituto de Investigación en Matemáticas Aplicadas y en Sistemas. Universidad Nacional Autónoma de México, Ciudad Universitaria, México City, México; ^3^ Instituto de Investigaciones en Matemáticas Aplicadas y en Sistemas, Universidad Nacional Autónoma de México, Unidad Académica Yucatán, Mérida, Mexico

**Keywords:** *Bifidobacterium*, gut microbiota, co-expression network, HMOS, WGCNA

## Abstract

Biological systems respond to environmental perturbations and a large diversity of compounds through gene interactions, and these genetic factors comprise complex networks. Experimental information from transcriptomic studies has allowed the identification of gene networks that contribute to our understanding of microbial adaptations. In this study, we analyzed the gene co-expression networks of three Bifidobacterium species in response to different types of human milk oligosaccharides (HMO) using weighted gene co-expression analysis (WGCNA). RNA-seq data obtained from Geo Datasets were obtained for *Bifidobacterium longum subsp. Infantis, Bifidobacterium bifidum and Bifidobacterium longum subsp. Longum*. Between 10 and 20 co-expressing modules were obtained for each dataset. HMO-associated genes appeared in the modules with more genes for *B. infantis* and *B. bifidum*, in contrast with *B. longum*. Hub genes were identified in each module, and in general they participated in conserved essential processes. Certain modules were differentially enriched with LacI-like transcription factors, and others with certain metabolic pathways such as the biosynthesis of secondary metabolites. The three Bifidobacterium transcriptomes showed distinct regulation patterns for HMO utilization. HMO-associated genes in *B. infantis* co-expressed in two modules according to their participation in galactose or N-Acetylglucosamine utilization. Instead, *B. bifidum* showed a less structured co-expression of genes participating in HMO utilization. Finally, this category of genes in *B. longum* clustered in a small module, indicating a lack of co-expression with main cell processes and suggesting a recent acquisition. This study highlights distinct co-expression architectures in these bifidobacterial genomes during HMO consumption, and contributes to understanding gene regulation and co-expression in these species of the gut microbiome.

## 1 Introduction

The human gut microbiota is a community of anaerobic microorganisms that plays an important role in the metabolization of complex carbohydrates that are not degradable by host enzymes (Flint et al., 2012). Their presence directly influences gastrointestinal physiology. Consumption of fiber and prebiotics has been considered positive for our health. These benefits include a reduced load of pathogens (Gibson, McCartney, and Rastall 2005; Markowiak and Śliżewska 2017), stimulation of the immune system (Shokryazdan et al., 2017), lower allergy rates (Brosseau et al., 2019), and production of short-chain fatty acids as metabolites resulting from their degradation (Tsukuda et al., 2021). For this reason, a great interest in this research has arisen, considering the contribution of the intestinal microbiota to our wellbeing (Couto et al., 2020).

Free human milk oligosaccharides (HMO) are the third most abundant component in human milk after lactose and lipids. They are structurally complex glycans composed of different monomer units that act as prebiotics (Castro et al., 2022). HMO can be classified into three groups: 1) HMO decorated with fucose or N-acetylneuraminic acid linked to lactose as a common core, producing neutral or acidic HMO such as 2′- or 3-fucosyllactose (FL), and 3′- or 6′-sialyllactose (SL); 2) type 1 HMO, characterized by lacto-N-biose (Galβ1-3GLcNAc) repeats attached to a lactose core, rendering molecules such as lacto-N-tetraose (LNT); and 3) type 2 HMO, composed of N-acetyllactosamine units (LacNAc; Galβ1-4GlcNAc) attached to a lactose core, forming molecules such as lacto-N-neotetraose (LNnT) (Thomson, Medina, and Garrido 2018).

The Bifidobacterium genus is the most dominant in the infant intestinal microbiota, stimulated by HMOs in the first years of life (Turroni et al., 2022). Most infant bifidobacteria are well known for their adaptations to the infant gut, displaying several mechanisms for utilization of HMO (Zhang et al., 2022). These include the presence in their genomes of several ATP-binding cassette (ABC) and major facilitator superfamily (MFS) transporters, glycolytic enzymes targeting different linkages in HMO, and feeder pathways deriving HMO molecules to central metabolism (Sakanaka et al., 2020). How bifidobacteria orchestrate molecular responses to HMO using transcriptional factors has been little studied.

RNA-sequencing (RNA-seq) is a powerful high-throughput technology that provides insights into differential gene expression and allows the identification of co-expressed genes in a particular condition (Ozsolak and Milos 2011). Using RNA-seq data provided from multiple samples, network analysis has been used as an approach to study biological systems. This analysis models the interaction of real biological networks and can be intuitively understood by users (DiLeo et al., 2011; Kukurba and Montgomery 2015; Chung et al., 2021). In the context of this analysis, a network has a set of nodes represented by genes and a set of edges, indicating significant co-expression relationships. In these networks, there are highly connected nodes (hubs) and a large number of nodes with a small number of connections. Both maintain the structural properties of real networks, such as scale-free topology (Stuart et al., 2003; Galán-Vásquez and Perez-Rueda 2019).

Weighted gene co-expression network analysis (WGCNA) is a practical methodology for network reconstruction that considers the co-expression patterns between two genes, and the overlapping of neighbor genes (Langfelder and Horvath 2008). To do this, clusters of co-expressed molecules known as modules are constructed, reflecting different groups (Zhang and Horvath 2005; DiLeo et al., 2011). The data are included in an adjacency matrix, in which the linkage intensity between genes is defined. Also, a soft threshold parameter is used, which is essential in reconstructing the network. Then, a topological overlap measure (TOM) is implemented as a proximity measure of genes in network modules that combine the adjacency of two genes and the intensity of their connections with neighboring genes. Gene co-expression networks have been used to predict functions of unknown genes, possible relationships with diseases (Liao et al., 2020; Rezaei et al., 2022), or how microorganisms behave in response to intra- or extracellular signals (Duran-Pinedo et al., 2011). Transcriptional factors (TFs) and metabolic enzymes have been found and considered as conserved processes between organisms focused on gene regulation and metabolism (Jha et al., 2020). Due to its relevance in the genome and microbial adaptations, it is essential to evaluate how gene regulation patterns are present to explain the biological activity in each module.

Some studies have demonstrated that the complexity in the structure of HMOs drives species of the Bifidobacterium genus to adapt their gene expression for their molecular utilization (Alessandri, van Sinderen, and Ventura 2021). These changes allow the production of enzymes necessary for processing these compounds. For example, *B. longum* subsp. Infantis (*B. infantis*) can internalize intact HMOs through ABC transporters, which are broken down into monomers for intracellular metabolization (Masi and Stewart 2022). In contrast, *B. bifidum* produces extracellular enzymes that degrade the oligosaccharides and internalize the monomers (Kitaoka 2012). The subspecies *B. longum* subsp. Longum (*B. longum*) can grow on various HMO structures, with a greater preference to fucosylated HMO. HMO-utilization genes are usually contained in discrete clusters, controlled by predicted TFs, and scattered across the genome (Duar et al., 2020). *B. infantis* ATCC 15697 is characterized by at least four HMO clusters, one devoted to type 1 HMO (LNB/GNB cluster), another for fucosylated and type 2 HMO consumption (HMO Cluster I), and two cluster targeting FL (Sela et al., 2008; Dedon et al., 2020; Zabel et al., 2020). *B. longum* species are thought to be adapted to the adult gut microbiota, but some isolates also contain HMO clusters allowing type 1 and FL consumption (Sakanaka et al., 2019).

Although molecular strategies for HMO utilization have been well described recently, it is unknown how transcriptional responses to these molecules are orchestrated at genome levels. Furthermore, despite the fact that all these organisms belong to the Bifidobacterium genus, they have unique HMO utilization patterns, so the regulation of these responses might differ. Therefore, in this work, gene co-expression networks based on WGCNA were constructed and studied for three representative species of the Bifidobacterium genus during HMO utilization. Also, co-expressed modules are identified and evaluated with regulatory proteins and metabolic maps to identify unexplored co-expression patterns.

## 2 Materials and methods

### 2.1 Datasets

The gene expression dataset was obtained from NCBI Geo DataSets (https://www.ncbi.nlm.nih.gov/gds). The dataset includes 18 RNA-seq samples of transcriptome response for *B. infantis* ATCC 15697 (GSE58773) and *B. longum* SC596 (GSE87697), excepting *B. bifidum* SC555 (GSE59053) with 20 samples (mucin as carbon source included) to pooled and individual HMO (Garrido et al., 2015; 2016). Among evaluated individual substrates were LNT, LNnT, 2FL, 3FL, and 6SL. Pooled HMOs were evaluated at the early (OD600 nm = 0.25), middle (OD600 nm = 0.5–0.7), and late time (OD600 nm = 0.9–1.1) points of growth (Supplementary Table S1).

**FIGURE 1:**
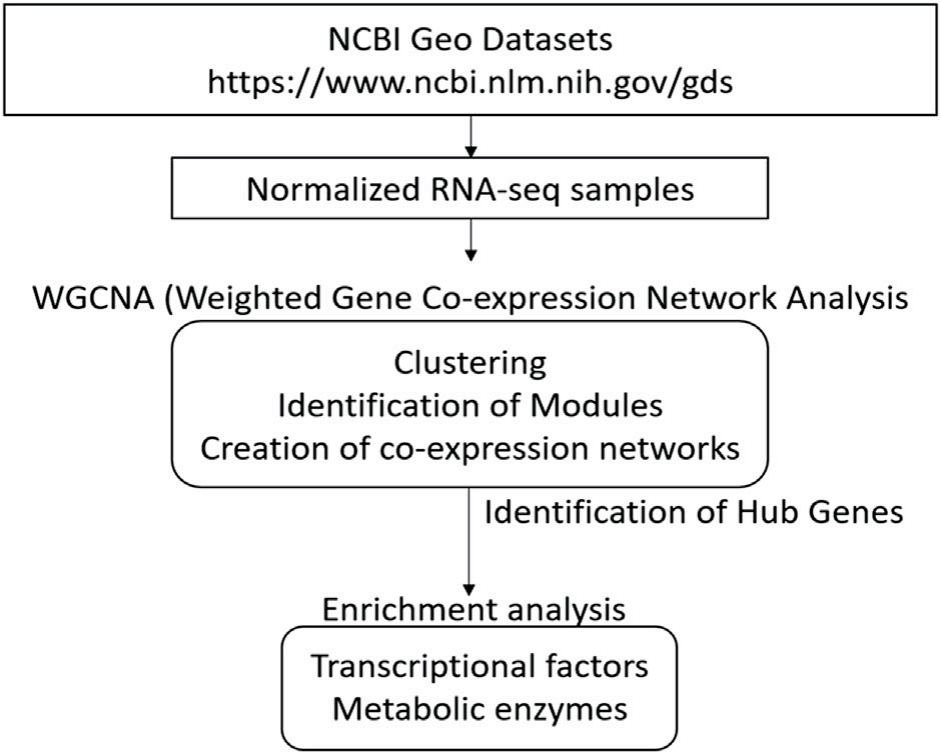
Overview for conducting Weighted Correlation Network Analyses (WGCNA) of different strains of Bifidobacterium genus using several HMOs. This schematization represents all steps since acquisition of sample dataset, WGCNA processing, until analysis of networks and identification of hub genes.

GoodSamplesgenes function on the WGCNA R package was used to inspect the dataset results for missing values. Genes and samples classified as “good genes” and “good samples” were respectively conserved (Langfelder and Horvath 2008). A schematic representation of all analysis procedures is included in Figure 1.

### 2.2 Construction of co-expression networks

Gene co-expression networks were created through WGCNA in R from normalized samples (Supplementary Figure S1). This package was useful for clustering samples, module distribution, and determining topological properties (Langfelder and Horvath 2008). Power (β) values were calculated per organism using pickSoftThereshold function (Table 1). Scale-free topology properties of biological networks were added for this purpose. Then, an adjacency matrix was constructed for each bacterial strain using correlation networks, where negative correlations in genes were considered unconnected.

**TABLE 1:** Overview of dataset and co-expression modules in this study.

Genome	Sample	Genes in WGCNA	Size max/Min genes per module	β	Genes in Grey Module	Coverage	# Modules without grey	# Genes delete in GoodSampleGenes
*B. longum infantis* ATCC 15697	18	2,577	516/27	12	0	2,577	20	0
*B. bifidum* SC555	20	1894	532/23	12	2	1892	10	0
*B. longum* subsp. *Longum* SC596	18	2,251	700/66	16	125	2,219	11	7

After, the adjacency matrix was converted into a TOM matrix to minimize noise effects and spurious associations. Higher TOM values allowed the identification of gene modules. Therefore, signed correlation networks were used, pairwise biweight midcorrelation coefficients and β values. Clustered genes were put into modules with analog expression patterns using the average linkage hierarchical clustering algorithm (flashClust function). The cutreeDynamic function cut the dendrogram branches and generated the gene modules. It used 1-TOM as a distance or dissimilarity matrix with a minimum module size equal to 20. Finally, modules with highly correlated eigengenes were merged based on a minimum height of 0.20 (mergeCloseModules function). Each module was differentiated by a specific color, where grey was set for uncorrelated and discarded genes (Horvath 2011). The rest of the modules were renamed with a number.

Modules were exported using the exportNetworkToCytoscape function to analyze hubs about modules of interest. The 100 most highly correlated genes were chosen for each module. The hub genes were those most highly connected nodes within the module, therefore, the degree of connectivity for each node (K) was calculated, which is defined as the number of edges adjacent to each node (Junker and Schreiber 2008). For smaller modules, genes were filtered to the top 50% according to the threshold value of the correlation. KEEG ortholog categories were assigned to each gene and the legend was created using Legend Creator App on Cytoscape.A general description of data information of genomes was included in Table 1 and all scripts in Supplementary Section.

### 2.3 Distribution of TFs and enzymes in modules

Each *Bifidobacterium* genome gene was associated with its Enzyme Commission (EC) number from the Kyoto Encyclopedia of Genes and Genomes (KEGG) database (Kanehisa and Goto 2000). Each enzyme with an E.C. number was related to its respective metabolic map. Similarly, for TFs, we used the compendium of TFs predicted by (Flores-Bautista et al., 2020), assigned from the hidden Markov model (HMM) profiles, and orthologous comparisons. The abundance and distribution of each dataset were determined by calculating an incidence rate and heatmap for each genome.

### 2.4 Enrichment analysis

Enrichment analysis was necessary to evaluate the functional relationship between obtained modules, TFs, and enzymes through a hypergeometric test. Statistical significance at a *p*-value of <0.05 was set. The enrichment analysis was performed using in house scripts in Python language (https://www.python.org/).

## 3 Results

### 3.1 Construction of gene co-expression networks

**FIGURE 2:**
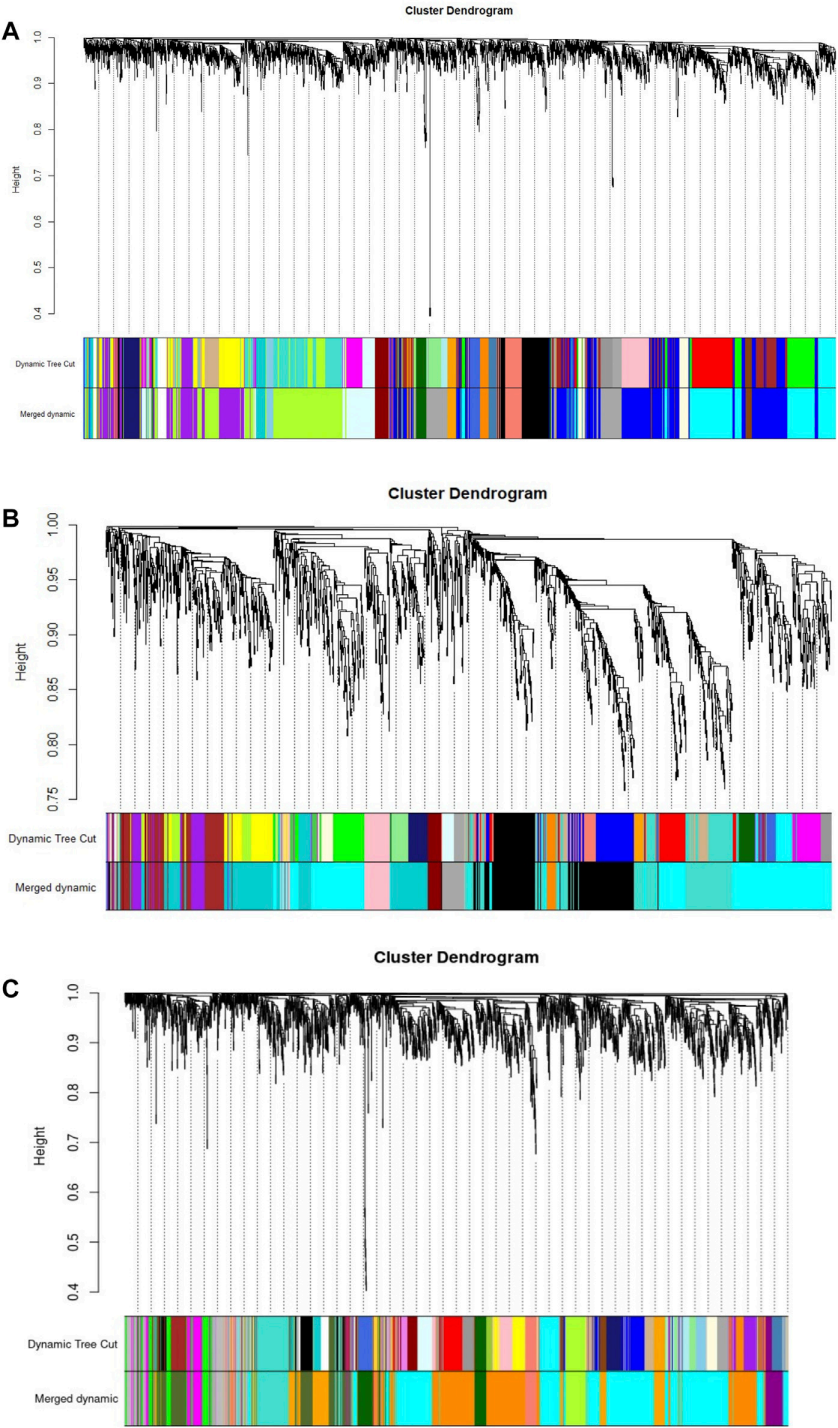
Gene clustering, with dissimilarity based on topological overlap (TOM), with the corresponding module colors indicated by the color row (Merged dynamic). **(A)** *B. longum* subsp. *Infantis* ATCC 15697. **(B)** *B. bifidum* SC555, and **(C)** *B. longum* subsp. *Longum* SC596. Each colored row represents a color-coded module which contains a group of highly connected genes.

A diagram of the analyses performed in this study is shown in Figure 1. All RNA-req samples for each Bifidobacterium strain were obtained from NCBI Geo Datasets (Garrido et al., 2015; 2016). For WGCNA analysis, log10 normalized read counts for all samples were taken from NCBI Geo Datasets (Supplementary Figure S1). RNA-seq datasets (GSE58773, GSE87697, GSE59053) were evaluated by sample clustering according to the Euclidean distance between different samples observed for each bacterium (Figure 2). No outliers were detected in the clusters; therefore, 56 samples were used to construct a hierarchical clustering tree (Supplementary Figure S2).

**FIGURE 3:**
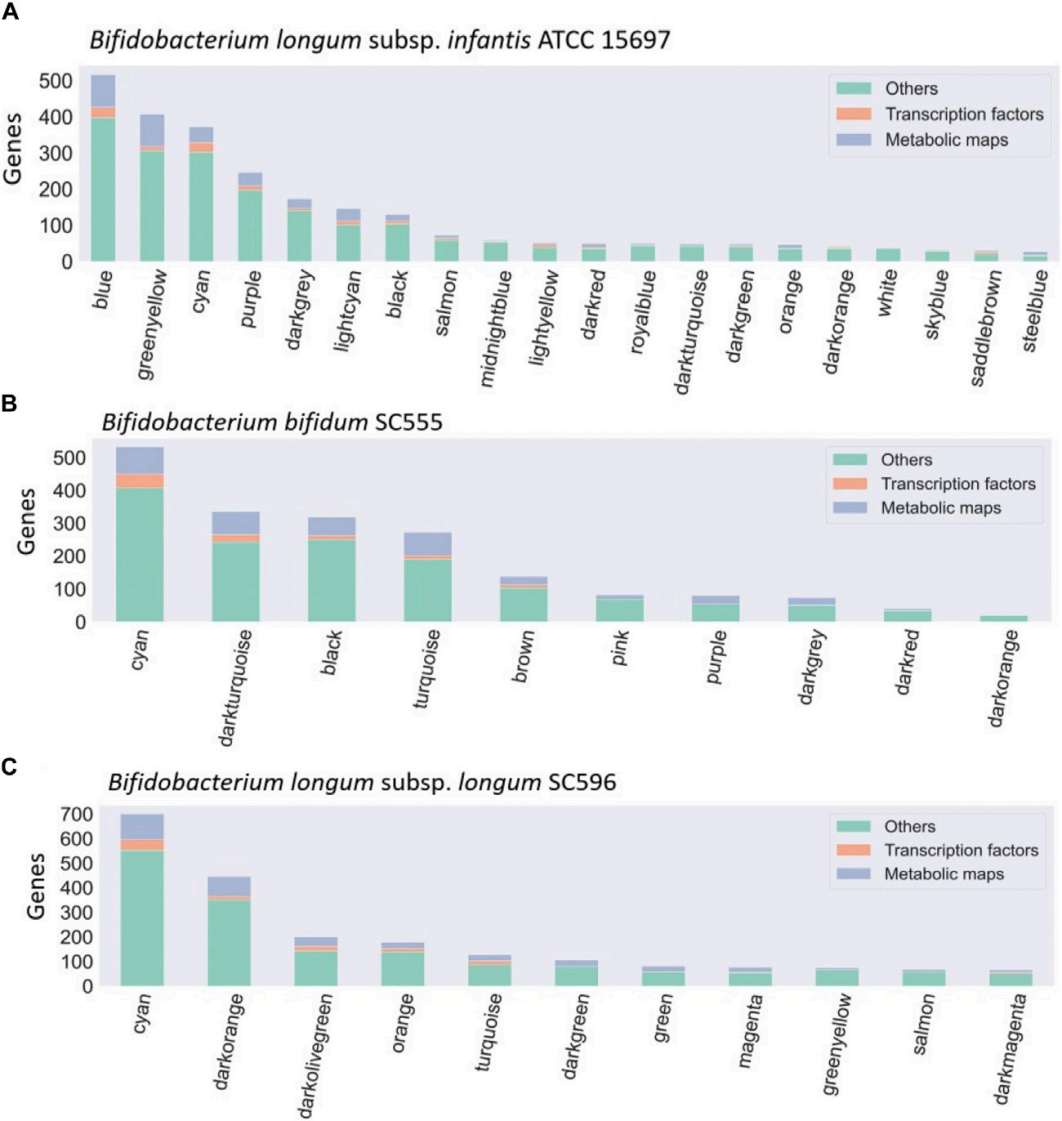
Bacteria co-expression modules in **(A)** B. *infantis* ATCC 15697, **(B)** *B. bifidum* SC55, and **(C)** B. *longum* subsp. *longum* SC596. On the x-axis are shown the modules identified with the WGCNA package, identified with a number. The distribution of modules is represented in decreasing order, where the y-axis represents the number of genes per module. Each module is made up of a set of genes associated with TFs (orange), metabolic maps (blue), and others unclassified genes (green).

WGCNA identified 20 modules for *B. infantis*, 10 for *B. bifidum* SC555, and 11 for *B. longum* SC596 (Figure 3). The soft-threshold power was adjusted to 12, 12, and 16 for *B. infantis* ATCC 15697, *B. bifidum* SC555, and *B. longum* SC596, respectively. These values were selected to define the adjacency matrix based on the criterion of approximate scale-free topology (Supplementary Figure S3), with a minimum module size of 20, and 0.20 cut height for merging of modules, which means that the modules whose eigengenes are correlated above 0.80 must be merged (Supplementary Figure S4).

Dominant modules such as Blue and Greenyellow in *B. infantis*, included sugar transport proteins and carbohydrate metabolism genes (Supplementary Table S2; Supplementary Table S3). For *B. bifidum* SC555, the Cyan module was the most extensive module exceeding 500 genes (Figure 3). This module contained diverse categories of biological functions, such as genes encoding sugar transporter proteins, transcriptional regulators, ribosomal proteins, MFS transporters, ATP-binding proteins, and central metabolism genes. However, this module did not contain any HMO-related gene. All modules in *B. bifidum* contained regulatory and metabolic pathway genes. The Darkturquoise and Purple modules included the highest number of HMO genes, with 13 and 12, respectively (Supplementary Table S4; Supplementary Table S5). Finally, in *B. longum* SC596 the Cyan module was the largest (Figure 3). Darkgreen and Green modules in this genome contained carbohydrate metabolism genes specifically for HMOs, not being dominant compared to other modules (Supplementary Table S6; Supplementary Table S7).

### 3.2 Identification of hub genes

Later, the top 100 most highly correlated genes were chosen for each module in order to identify hub genes. These are determined considering the most highly connected node within the module, calculating the degree of connectivity for each node. For smaller modules, genes were filtered to the top 50% according to the correlation threshold value. Hub genes are shown in Supplementary Tables S8–S10. They contained diverse functions, from a hypothetical protein with a transmembrane domain, ABC permeases not related to HMO utilization, metabolic enzymes, and a transposase in *B. infantis* (Supplementary Table S8). Hub genes in *B. bifidum* modules appeared to be related to protein synthesis (Supplementary Table S9) and in *B. longum* were more varied and included proteases, cell division and transport proteins (Supplementary Table S10). These highly connected genes could be important for bifidobacterial physiology or HMO metabolism, and their actual role in these processes could be validated using directed mutagenesis.

### 3.3 Enrichment analysis for TFs and metabolic pathways

Gene regulation and metabolism are among the most conserved processes among microorganisms. They are characterized by DNA-binding regulatory proteins and enzymes involved in metabolic processes (Downs 2003; Schmid 2018). The distributions between modules were mapped to determine similar co-expression patterns between metabolism and gene regulation processes. Consequently, TFs from Hidden Markov model (HMM) profiles and enzymes from the KEGG database were associated with the genes of each Bifidobacterium strain.

**FIGURE 4:**
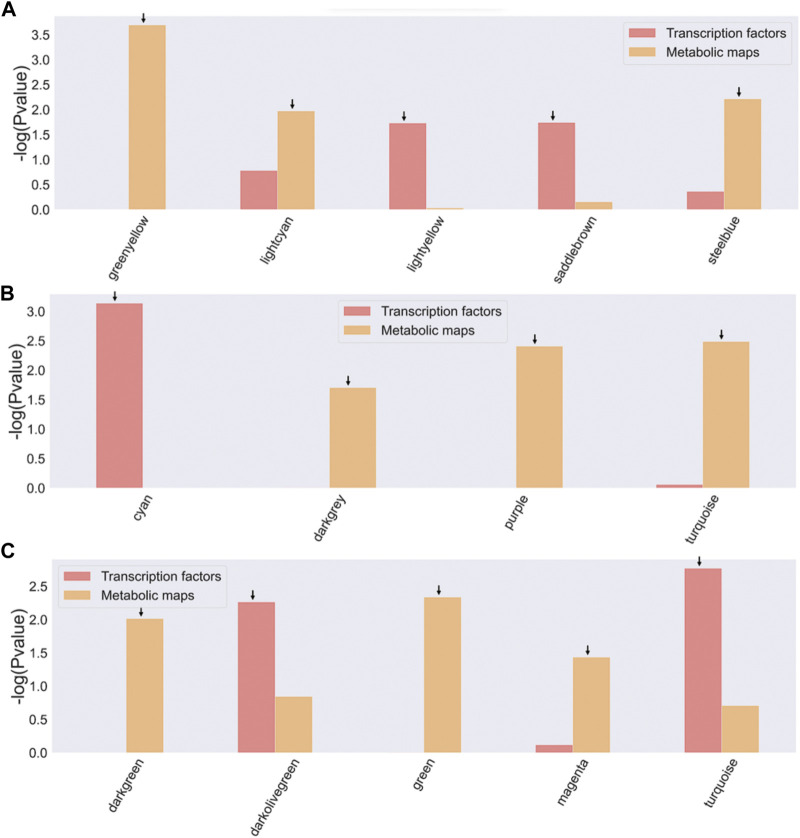
Enrichment of TFs and metabolic maps for Bifidobacterium strains. **(A)** *B. longum* subsp. *Infantis* ATCC 15697, *B bifidum* SC555, **(C)** *B. longum* subsp. *Longum* SC596. Modules with a −log10 (*p*-value) > 1.5 (corresponding to a *p*-value <0.05) were selected as enriched and are indicated by an arrow on the bar. The red bars represent modules enriched with TF families, and the orange bars represent modules enriched with metabolic map.

For all three bifidobacterial genomes, enzymes involved in metabolic processes and TFs were found in almost all co-expression network modules (Figure 3). However, some modules contained a significant enrichment (-log10 (*p*-value) > 1.5) in TFs or metabolic pathways across the three genomes (Figure 4). Enriched modules with HMO-associated genes differed from those enriched with TFs (Figure 4). The most enriched modules with TFs contained on average 15.48% of the predicted genes with this function in *B. infantis* modules, an 8.08% average for *B. bifidum* SC555, and 10.65% average for *B. longum* SC596. Meanwhile, the modules enriched with metabolic enzymes contained on average 27.47% genes predicted to be related to metabolism in *B. infantis*, 29.57% for *B. bifidum* SC555, and 26.20% for *B. longum* SC596. Analyzing the enriched functions identified, there was at least one module with a high percentage of TFs and enzymes in each bacterium. This case led us to evaluate whether the richer modules prefer a particular TF family or metabolic maps.

### 3.4 TFs and bifidobacterial metabolism

**FIGURE 5:**
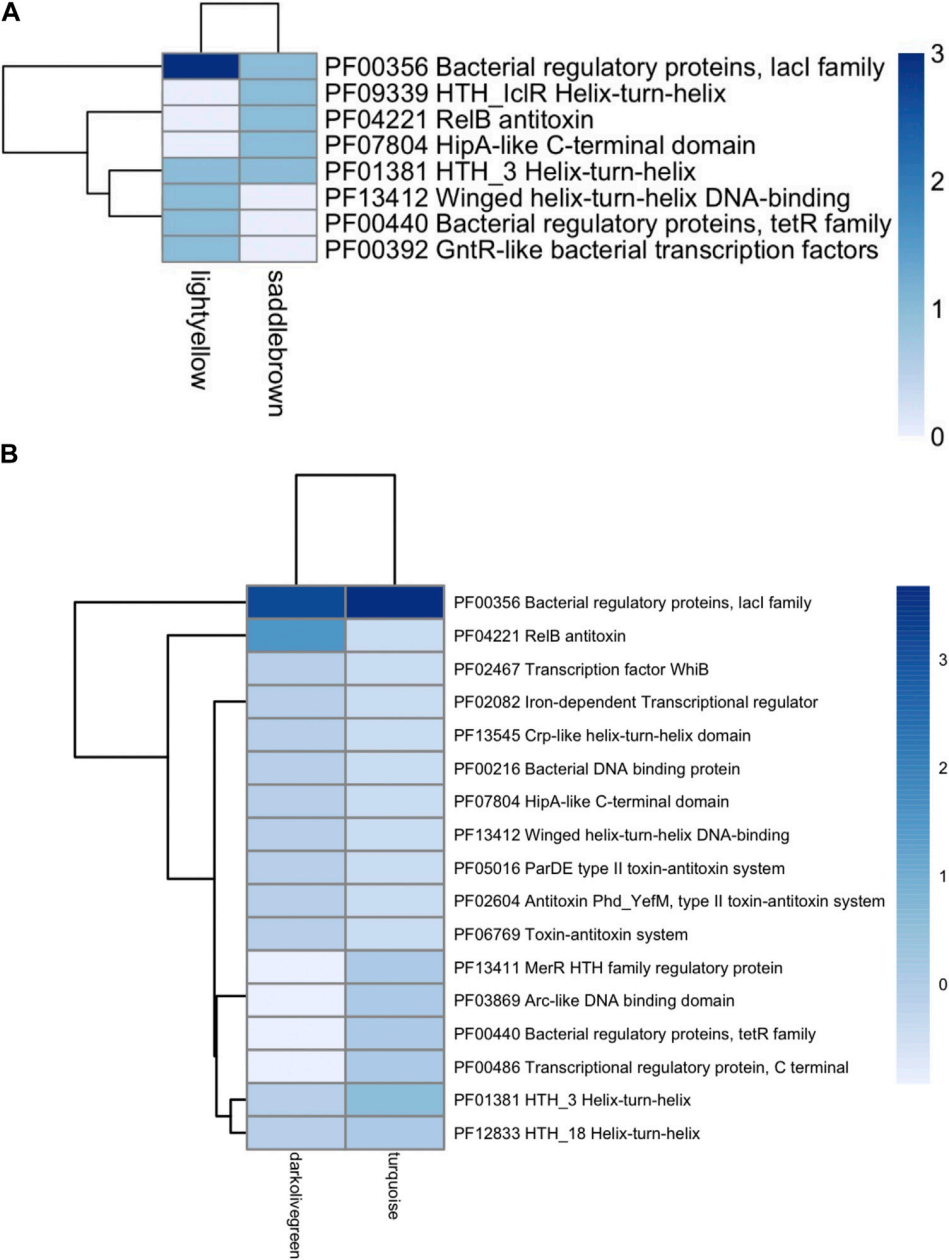
Heat map of TFs abundance for Bifidobacterium species. **(A)** *B. longum* subsp. *Infantis* ATCC 15697, **(B)** *B. longum* subsp. *Longum* SC596. Each row represents the PFAM, and each column represents the most enriched module for that bacterial species.

Each TF family of the enriched modules in Figure 4 was classified using the Pfam database and the z-score of frequencies of the clustered families (Sun et al., 2017). This clustering was performed by determining the Euclidean distance measure and Ward’s method for linkage analysis (Dvorak et al., 2017). Overall, the TF with the highest frequency was the LacI family (PF00356) in each enriched module (Figure 5). This family is usually associated with regulation of carbohydrate metabolism. Other TFs were identified among evaluated Bifidobacterium genomes, such as TetR family (PF00440). TetR was present in the Lightyellow module in *B. infantis*, Cyan module in *B. bifidum* SC555 (Table 2), and Turquoise module in *B. longum* SC596. GntR family (PF00392) was also identified in both *B. infantis* and *B. bifidum* SC555, but not in *B. longum* SC596.

**TABLE 2:** TF families found in Cyan as an Enriched module in *B. bifidum*.

Name	Pfam family	Frequency
MerR family regulatory protein	PF00376	1.0
Bacterial regulatory proteins, tetR family	PF00440	2.0
MerR HTH family regulatory protein	PF131411	2.0
GntR-like bacterial transcription factors	PF00392	2.0
MarR family	PF01047	1.0
Transcription factor WhiB	PF02467	1.0
Bacterial regulatory proteins, lacI family	PF00356	5.0
RelB antitoxin	PF04221	3.0
Transcriptional regulatory protein, C terminal	PF00486	3.0
LuxR-type DNA-binding HTH domain	PF00196	3.0
PspC domain	PF04024	1.0
Cold-shock domain	PF00313	1.0
Repressor lexA	PF01726	1.0
Helix-turn-helix	PF09339	1.0
	PF01381	2.0
	PF13936	2.0
PucR C-terminal helix-turn-helix domain	PF01402	1.0
Ribbon-helix-helix protein, copG family	PF13412	1.0
Winged helix-turn-helix DNA-binding	PF01418	1.0

**FIGURE 6:**
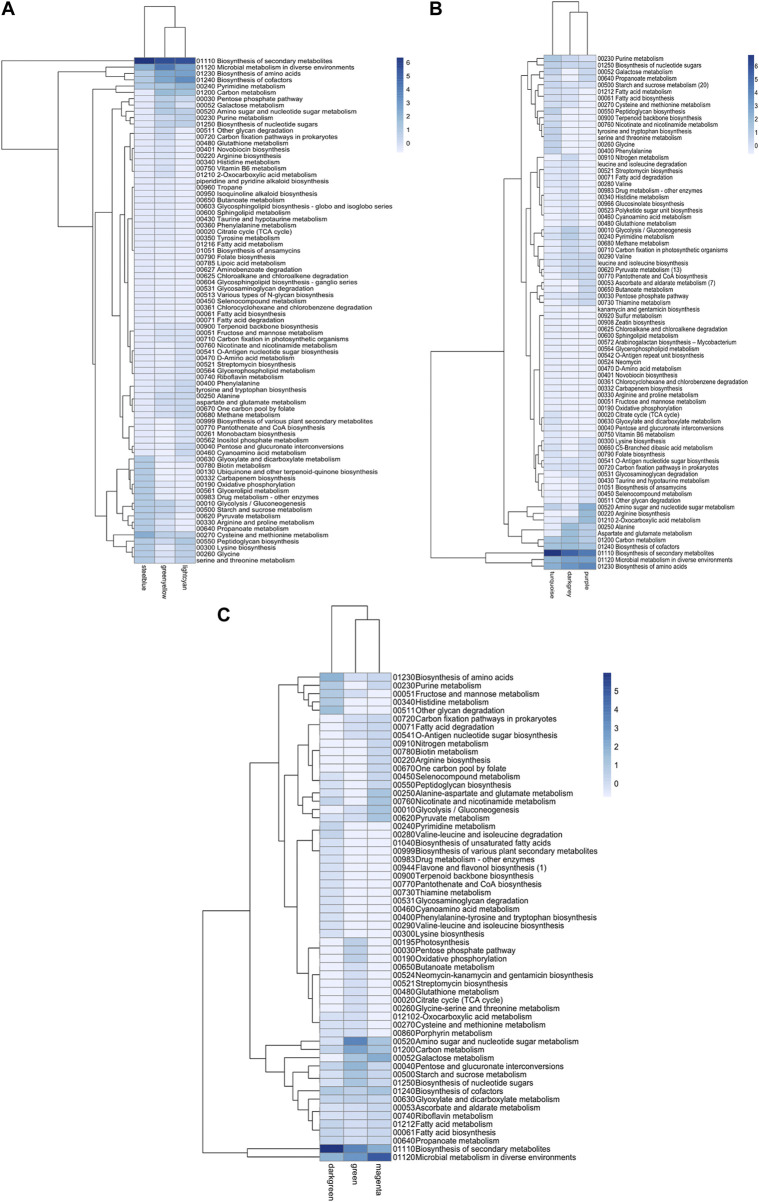
Metabolic map abundance in *Bifidobacterium* species. **(A)** B. *infantis* ATCC 15697, **(B)** B. *bifidum* SC555, **(C)** B. *longum* subsp. *longum* SC596. Each row represents a metabolic map (KEGG), and each column represents the most enriched module.

Similarly, metabolic enzymes were classified according to the KEGG pathway database (Kanehisa et al., 2019). z-scores of the frequency of each metabolic map were clustered together, similar to those performed for transcriptional factors (Figure 6). Modules significantly enriched were characterized by an overabundance of general metabolic pathways, followed by biosynthesis of amino acids, cofactors, and secondary metabolites. This trend was common for the three transcriptomes studied. Biosynthesis of secondary metabolites was the only category differentially enriched among modules (Figure 6).

### 3.5 Metabolism and regulation for HMOs consumption

**FIGURE 7:**
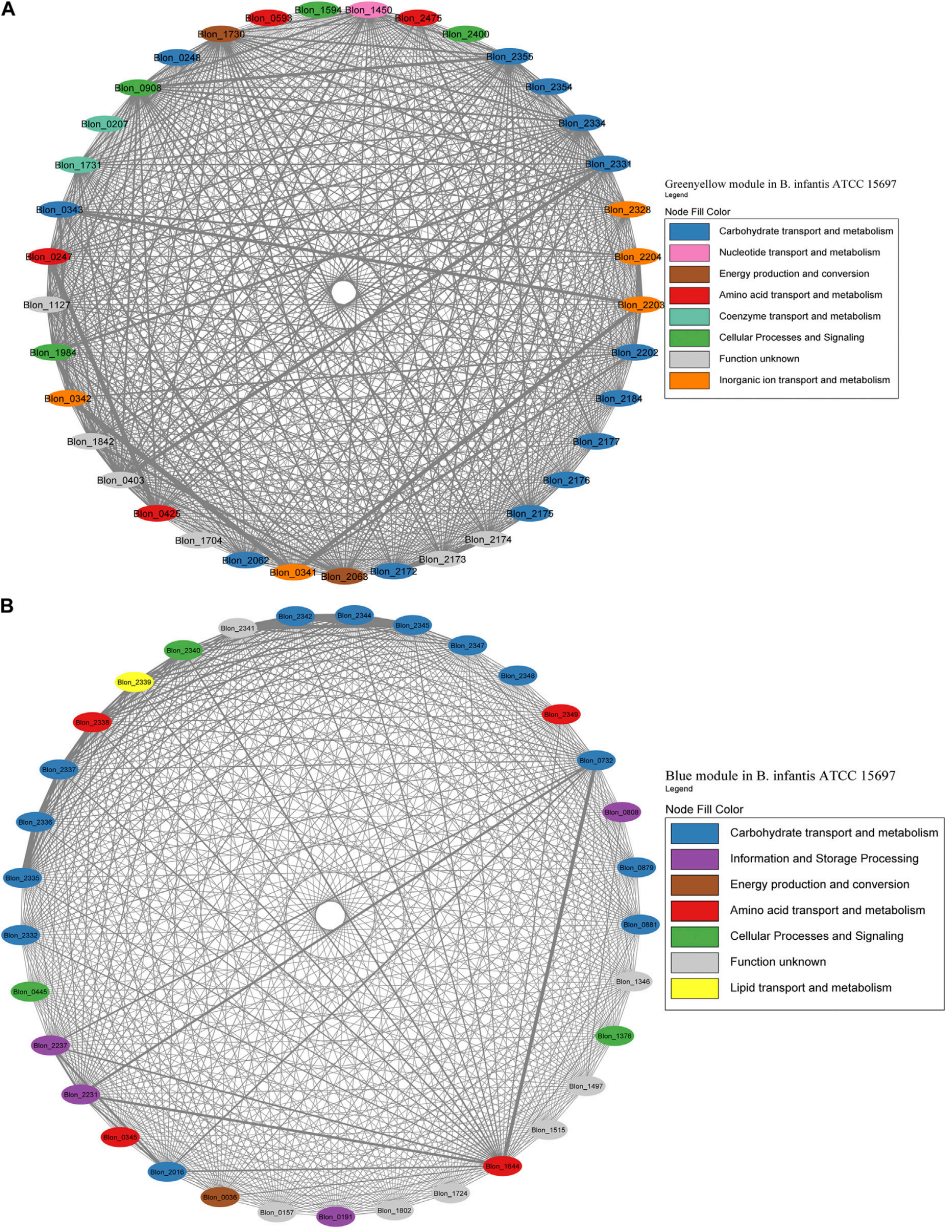
HMO-related co-expression networks in *B. infantis*. **(A)** Greenyellow and **(B)** Blue module in for *B. infantis*. All Network construction consisted of modules were manually analyzed, and those containing HMO-metabolizing genes were used to create a co-expression network. This analysis considered the neighboring genes of each node with the highest correlation.

**FIGURE 8:**
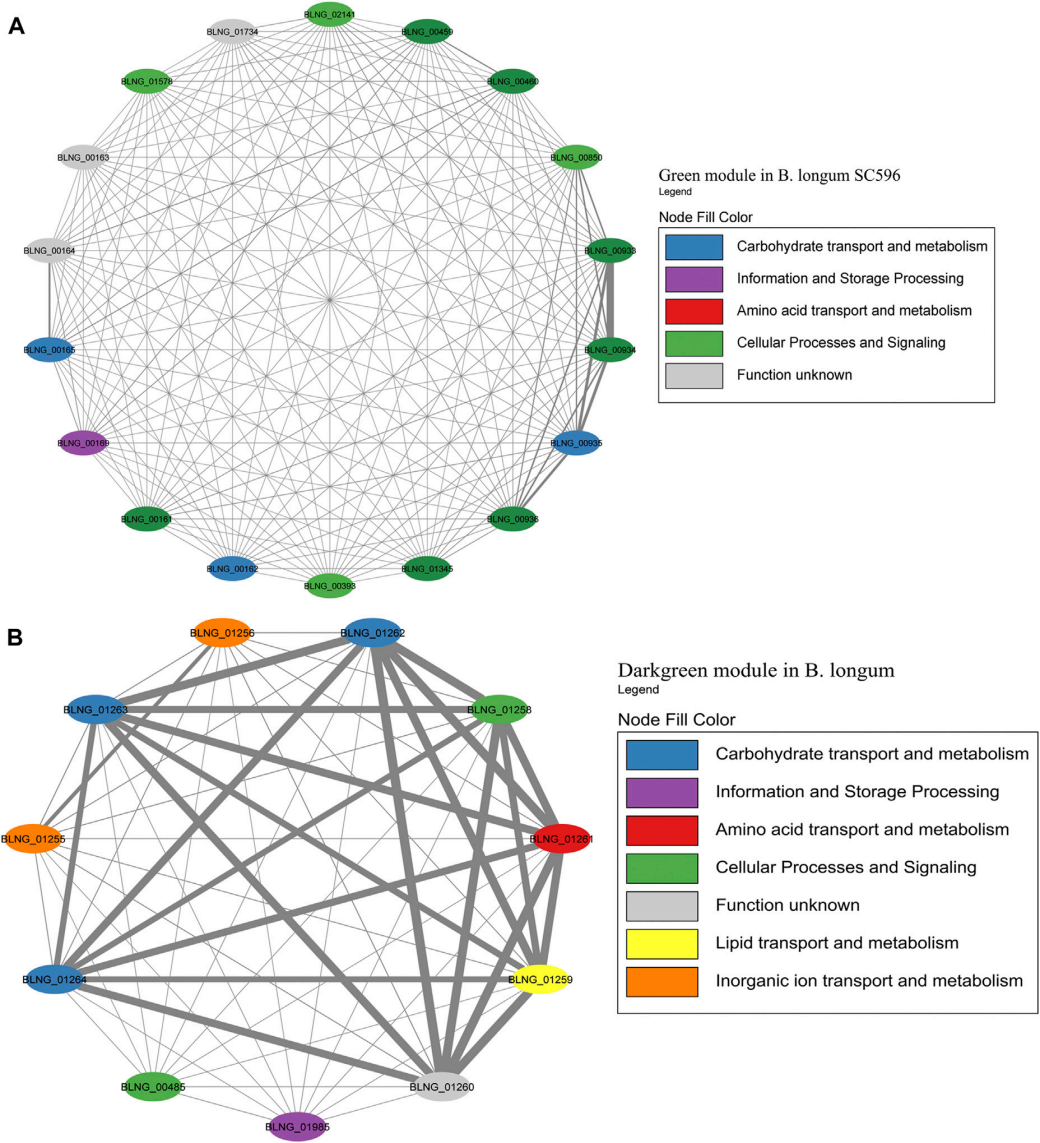
HMO-related co-expression networks in *B. bifidum*. **(A)** Darkturquoise and **(B)** Purple module for *B. bifidum* SC555. All Network construction consisted of modules were manually analyzed, and those containing HMO-metabolizing genes were used to create a co-expression network. This analysis considered the neighboring genes of each node with the highest correlation.

**FIGURE 9:**
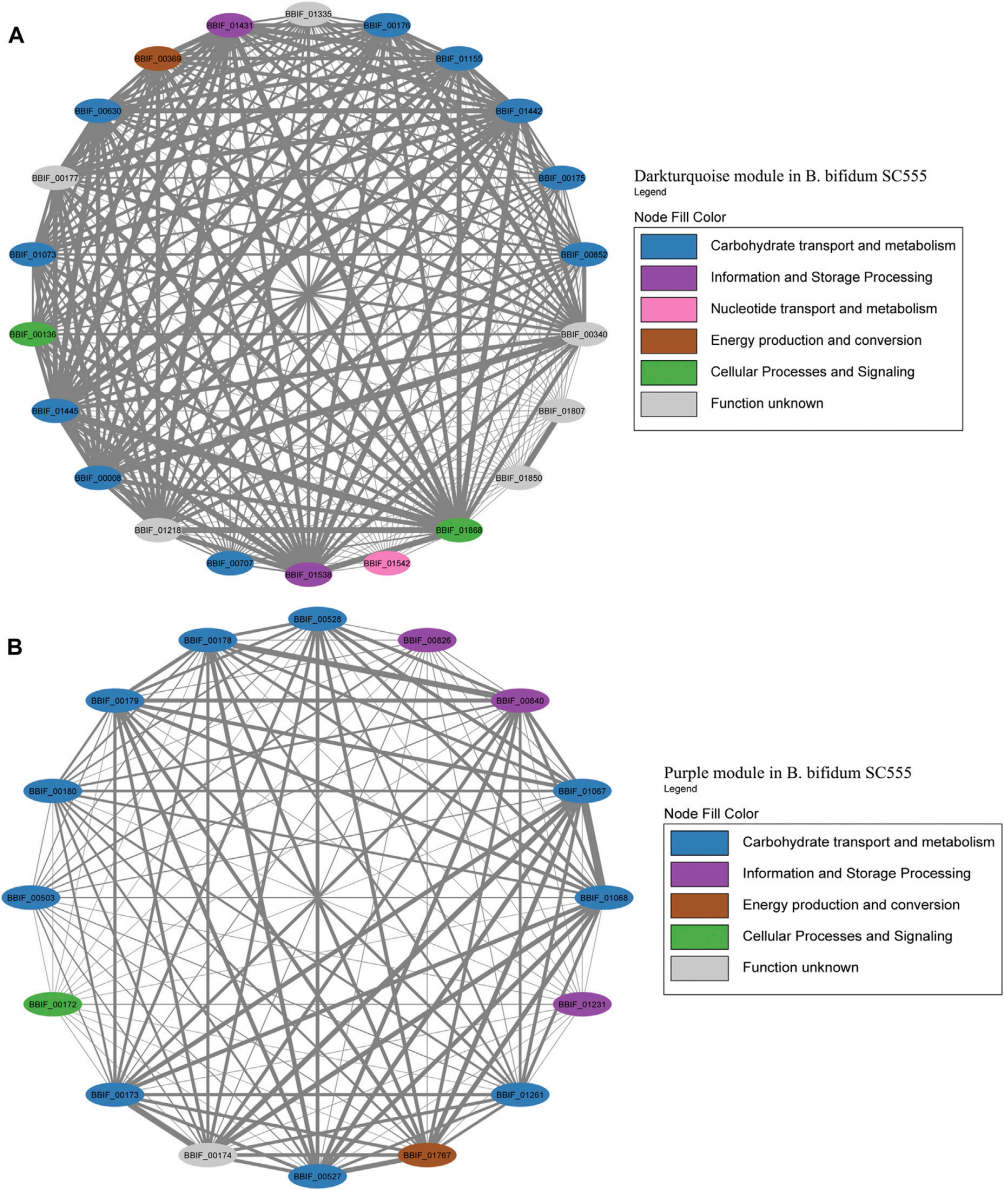
HMO-related co-expression networks in *B. longum*. **(A)** Green and **(B)** Darkgreen modules for *B. longum* SC596. All Network construction consisted of modules were manually analyzed, and those containing HMO-metabolizing genes were used to create a co-expression network. This analysis considered the neighboring genes of each node with the highest correlation.

Later, modules were manually analyzed, and those containing HMO-metabolizing genes were used to create co-expression networks (Figures 7–9). This analysis considered the neighboring genes of each node with the highest correlation. Selected modules were Greenyellow and Blue in *B. infantis*, Darkturquoise and Purple in *B. bifidum*, and Green and Darkgreen in *B. longum* (Figures 7–9).

The Greenyellow module network included 22 HMO-related genes (Figure 7A; Supplementary Table S2). These genes include transporters and enzymes for FL metabolism (Blon_0247, Blon_0248, Blon_0341 - Blon_0343, Blon_2202 - Blon_2204), which showed an important degree of co-expression. The Greenyellow network also included the GNB/LNB and type 1 HMO processing cluster (Blon_2172-Blon_2177), and galactose metabolism enzymes (Blon_2062-Blon_2063, Blon_2184) (Supplementary Table S1). Therefore, this module appears to orchestrate metabolic responses to HMO-derived galactose and fucose. Interestingly, a few genes of the complete HMO cluster I (Sela et al., 2008) appeared in this module (Blon_2331, permease; Blon_2334, β-galactosidase; Blon_2354, SBP; Blon_2355, hexosaminidase). This suggests that the HMO cluster I does not behave as one single transcriptional unit.

The Blue module for *B. infantis* contained 19 genes related to HMO, being the second with the highest number of genes of this category (Figure 7B; Supplementary Table S3). This module showed a higher degree of connectivity between nodes compared to the Greenyellow. The remaining genes of the HMO cluster I were included in this module, containing functions such as ABC transporters, fucose metabolism and HMO-glycolytic enzymes. These genes displayed a high co-expression (Figure 7B). Outside this cluster, the module also contained a β-N-acetylhexosaminidase (Blon_0732), TFs and enzymes for GlcNAc metabolism (Blon_0879, Blon_0881), and a β-galactosidase (Blon_2016). Blon_0732 showed high co-expression with two ribosomal proteins and an MFS porter. Other single HMO-utilization genes were scattered in several modules (Supplementary Table S3).

For *B. bifidum*, the highest number of genes related to HMO consumption was included in the Darkturquoise module, with 13 genes (Figure 8A). Modules in *B. bifidum* showed a less structured organization compared to *B. infantis*. The Darkturquoise module contained glycolytic enzymes, TFs for GlcNAc metabolism, and LNB processing enzymes (Figure 8A; Supplementary Table S4). The Purple module in *B. bifidum* contained 12 HMO-utilization genes related to galactose metabolism, transport, GlcNAc metabolism and an α-L-fucosidase (BBIF_01261). These genes showed a high degree of connectivity (Figure 8B; Supplementary Table S5). The Turquoise module contained five genes for galactose metabolism (BBIF_00368, BBIF_00550, BBIF_00871). Other HMO-utilization genes were found dispersed in other modules. Within the network, a total of nine genes unrelated to HMO utilization such as hypothetical proteins, a cell wall biosynthesis protein, and a transcriptional regulator, among others, were identified.

The Green module contained the highest number of genes in *B. longum*, with 12 HMO-related genes (Figure 9A; Supplementary Table S6). This module was only the seventh with the higher number of genes in *B. longum* (Figure 2C), suggesting HMO responses are rather modular in this species and do not co-express with other conserved processes. The Green module contained HMO transport (BLNG_00161, BLNG_00162, BLNG_00933-BLNG_00936, BLNG_01345) and galactose metabolism genes (BLNG_0163-BLNG_00165, BLNG_00459, BLNG_00460). The Darkgreen was only the sixth module with the most genes in *B. longum* (Figure 3) and contained nine genes participating in FL transport (BLNG_01255, BLNG_01256), fucose metabolism (BLNG_01258-BLNG_01262), and α-L-fucosidases (BLNG_01263, BLNG_01264) (Figure 9B; Supplementary Table S7). This modularity and lack of association with other cellular processes suggests a recent acquisition of this gene cluster. Finally, other genes in *B. longum* appeared as pairs or single in other modules.

## 4 Discussion

Bifidobacteria are important members of the gut microbiota in both infants and adults. HMOs are considered the primary substrates for the abundance of these species in the infant gut (Walsh et al., 2020). Considering the predicted transcriptional responses to Bifidobacterium species mediated by the structural complexity of HMOs, likewise in order to have a more comprehensive and global understanding of the gene associations involved, gene co-expression networks were constructed for three representative species such as *B. infantis*, *B. bifidum*, and *B. longum*.

Co-expression networks provide a simpler way to analyze genes that are correlated between biological processes that could be candidates, e.g., for the study of diseases (van Dam et al., 2017). WGCNA analysis has been successfully used as a system biology method for describing expression correlation patterns among genes across RNA samples corresponding to pooled and individual types of HMOs generating modules. By clustering the expression data, *B. infantis* possessed a higher number of modules compared to *B. bifidum* and *B. longum* (Figure 3). The number of modules in a co-expression network can be explained by multiple reasons, including physiological aspects such as adaptations to environmental circumstances and resource management between species (Duran-Pinedo et al., 2011). The variety of modules in *B. infantis* could indicate a more complex or diverse expression response compared to *B. bifidum* and *B. longum* (Horvath 2011; Duar et al., 2020; Zabel et al., 2020). Interestingly, some modules have been identified that group the majority of genes focused on metabolizing HMOs.

WGCNA modules may represent independent units responsible for certain biological functions (Jia, Zhao, and Jia 2020). This observation was similar to detecting biomarkers linked to gut microbiota (Vernocchi et al., 2020), and biofilm formation genes from the bacterial community (Liu et al., 2021a; Chen and Ma 2021). HMO metabolization could also be a multifactorial process that includes other genes concerned with other biological functions, as observed a co-expression study for *B. longum* FGSZY16M3 (Liu et al., 2021b). More experiments are needed to confirm the function of some unknown function genes and the relevance of identified hub genes in these networks.

Among TF regulatory processes found in enriched modules, LacI family (PF00356) was with a high frequency in each genome. LacI family transcriptional regulators are a group of allosteric DNA-binding regulators with conserved amino acid sequences (Lewis, 2005; Ravcheev et al., 2014). Most of the characterized LacI family transcriptional regulators sense sugar effectors and regulate carbohydrate utilization genes (Tsevelkhoroloo et al., 2021). Likewise, TetR family (PF00440) is known to be involved in multidrug resistance, and presumably controls the glucoside and galactoside utilization pathways (Ramos et al., 2005; De Bruyn et al., 2013). In the Bifidobacterium genus, TetR family has within its regulators the BgrT genes are co-localized with genes encoding various β-glucoside or β-galactoside hydrolases (e.g., bglB, bgaB, bglX, bglY), and β-glucoside or β-galactoside transporters of the MFS and ABC families. Specifically, BgrT1 regulon has been identified with the β-glucosidase gene bglX in *B. infantis*. For *B. longum* NCC2705, β-glucosidase genes bglX2 and bglX3 also have been identified with the BgrT2 regulon (Khoroshkin et al., 2016). In the case of *B. bifidum*, no regulator genes or regulons have yet been identified.

Concerning to HMO utilization, *B. infantis* is a dominant strain of the infant gut microbiota that can efficiently consume several classes of structures(Lawson et al., 2020; Ojima et al., 2022). HMO-related genes appeared to be sorted by functionality and activity of HMO clusters in the key modules. In the case of *B. bifidum* SC555, the organization of HMO-related genes can be explained by considering the consumption mechanism. *B. bifidum* prefers short HMOs, using enzymes to release fucose and sialic acid decorations on the oligos, which it ultimately does not use. This preference may also be directed to other carbon sources (Garrido et al., 2015). It should be highlighted that the HMO cluster I is found specifically in *B. infantis*, while fucose clusters are found in some *B. longum*, B. breve and *B. bifidum* strains. The LNT utilization cluster (Blon_2171—Blon_2177) is present in most infant-associated bifidobacteria (Zúñiga, Monedero, and Yebra 2018), and in *B. infantis* clustered with 2FL and galactose pathways.

A few *B. longum* strains can utilize certain HMOs, especially LNT and 2FL (Díaz et al., 2021). However, this subspecies is adapted to the adult gut microbiome (Díaz et al., 2021). *B. longum* SC596 is a strain isolated from an infant. *B. longum* key modules appeared to be closer to the HMO utilization genes, considering that Green module contained HMO transport and galactose metabolism genes (Figure 9A), and Darkgreen module was addressed to genes of FL metabolism and transport, including additionally fucosidases (Figure 9B). Previous transcriptomic analyses have revealed the affinity of *B. longum* to consume fucosylated HMOs allowing the strain to be more selective to HMO consumption compared to *B. infantis*, being an evolved strain (Garrido et al., 2016). This observation points at the Darkgreen as a single fucose metabolism module.

Regarding hub genes among three evaluated genomes (Supplementary Tables S8–S10), hypothetical, transport, and ribosomal proteins were identified as the most connected genes among modules. Hypothetical proteins could take a role as glycosyl hydrolase in the utilization of HMOs. However, future analysis will allow us to determine their particular functional role (Galán-Vásquez and Perez-Rueda 2019). Likewise, inside smaller modules, some hub genes with 3, 4, 5, 6, and 7 degrees of connections were identified, considered the most important in the co-expression network. This study illustrates how HMO genetic responses in Bifidobacterium are coordinated according to the constituent monosaccharide: galactose, GlcNAc, and fucose responses usually appear in distinct modules and clusters. This correlates with monosaccharides potential role in activating or repressing TFs and triggering the expression of cognate clusters. This organization in the modules has also been previously observed, in the transcriptional response in denitrifying bacteria on carbon nanotubes, whereby WGCNA they have obtained specific modules corresponding to the activity of various types of carbon nanotube structures (Zheng et al., 2018).

Despite the extensive analysis of this study, there are still some limitations, such as low reports of WGCNA studies in bifidobacteria and the deficiency in gene annotation of novel isolates bacteria. More datasets from Bifidobacterium species under different conditions are required to refine their regulatory networks. In some cases, the most important hub and non-HMO-related genes encodes for hypothetical proteins, allowing future analysis to determine their functional role. Also, experimental validation of co-expression networks and incorporation of different species in utilizing HMOs could further provide a widely understanding. These experiments could be combined with mutational analysis of hub or essential genes. The results obtained by WGCNA in the Bifidobacterium strains tested with different HMOs provide evidence of expression patterns identified with genes unrelated to HMO metabolism.

## 5 Conclusion

In this work, we identified and analyzed modules considered metabolically and regulatory relevant in a set of infant bifidobacteria, using a weighted gene co-expression analysis method (WGCNA) in the context of HMO utilization. From this analysis, we identified some modules enriched with TFs and metabolic enzymes. In the case of regulation, we identified TFs from the LacI, TetR, RelB, and HTH_3, GntR families, which are related to sugar utilization and biological processes, such as biosynthetic processes, and cellular metabolic processes. Our approach also identified genes involved in similar metabolic or regulatory functions. Among modules for Bifidobacterium strains, ABC and superfamily MFS transport proteins, transcriptional regulators such as TetR were identified as hubs genes because of their high correlation with other genes. The networks generated allowed us to identify co-expressed genes involved in responses to HMO consumption. Substantial differences were found in the structure of modules and regulation across these three Bifidobacterium species. In summary, this analysis allowed us to determine that, despite the diversity of experimental information available for each organism, these mechanisms are similar in all organisms, which will allow us to address new experimental results, such as the use of gene expression data in metagenomic studies.

## Data Availability

The original contributions presented in the study are included in the article/Supplementary Material, further inquiries can be directed to the corresponding author.

## References

[B1] Alessandri G. Douwe van S. Ventura M. (2021). The genus bifidobacterium: From genomics to functionality of an important component of the mammalian gut microbiota running title: Bifidobacterial adaptation to and interaction with the host. Comput. Struct. Biotechnol. J. 19 (1), 1472–1487. 10.1016/j.csbj.2021.03.006 33777340 PMC7979991

[B2] Brosseau C. Amandine S. Palmer D. J. Prescott S. L. Barbarot S. Bodinier M. (2019). Sébastien barbarot, and marie bodinierprebiotics: Mechanisms and preventive effects in allergy. Nutrients 11 (8), 1841. 10.3390/nu11081841 31398959 PMC6722770

[B3] Bruyn F. D. Beauprez J. Maertens J. Soetaert W. De Mey M. (2013). Unraveling the leloir pathway of bifidobacterium bifidum: Significance of the uridylyltransferases. Appl. Environ. Microbiol. 79 (22), 7028–7035. 10.1128/AEM.02460-13 24014529 PMC3811521

[B4] Castro I. García-Carral C. Furst A. Khwajazada S. García J. Arroyo R. (2022). Interactions between human milk oligosaccharides, microbiota and immune factors in milk of women with and without mastitis. Sci. Rep. 12 (1), 1367. 10.1038/s41598-022-05250-7 35079053 PMC8789856

[B5] Chen X. Ma J. (2021). Weighted gene Co-expression network analysis (WGCNA) to explore genes responsive to Streptococcus oralis biofilm and immune infiltration analysis in human gingival fibroblasts cells. Bioengineered 12 (1), 1054–1065. 10.1080/21655979.2021.1902697 33781179 PMC8806260

[B6] Chung M. Bruno V. M. Rasko D. A. Cuomo C. A. Muñoz J. F. Livny J. (2021). Best practices on the differential expression analysis of multi-species RNA-seq. Genome Biol. 22 (1), 121. 10.1186/s13059-021-02337-8 33926528 PMC8082843

[B7] Couto M. R. Gonçalves P. Magro F. Martel F. (2020). Microbiota-derived butyrate regulates intestinal inflammation: Focus on inflammatory bowel disease. Pharmacol. Res. 159, 104947. 10.1016/j.phrs.2020.104947 32492488

[B8] Dam S. v. Võsa U. Adriaan van d. G. Franke L. Pedro d. Magalhães J. (2017). Gene Co-expression analysis for functional classification and gene–disease predictions. Briefings Bioinforma. 19 (4), 575–592. 10.1093/bib/bbw139 PMC6054162 28077403

[B9] Dedon L. R. Özcan E. Rani A. Sela D. A. (2020). Bifidobacterium infantis metabolizes 2′Fucosyllactose-derived and free fucose through a common catabolic pathway resulting in 1,2-propanediol secretion. Front. Nutr. 7, 583397. 10.3389/fnut.2020.583397 33330584 PMC7732495

[B10] Díaz R. Torres-Miranda A. Orellana G. Garrido D. (2021). Comparative genomic analysis of novel bifidobacterium longum subsp. longum strains reveals functional divergence in the human gut microbiota. Microorganisms 9 (9), 1906. 10.3390/microorganisms9091906 34576801 PMC8470182

[B11] DiLeo Ma. V. Strahan G. D. Meghan den B. Hoekenga O. A. (2011). Weighted correlation network analysis (WGCNA) applied to the tomato fruit metabolome. PLoS ONE 6 (10), 26683. 10.1371/journal.pone.0026683 PMC3198806 22039529

[B12] Downs D. (2003). Genomics and bacterial metabolism. Curr. Issues Mol. Biol. 5 (1), 17–25. 10.21775/cimb.005.017 12638661

[B13] Duar R. M. Casaburi G. RyanMitchellScofield D. L. N. C. Ortega Ramirez A. Barile D. (2020). Comparative genome analysis of bifidobacterium longum subsp. infantis strains reveals variation in human milk oligosaccharide utilization genes among commercial probiotics. Nutrients 12 (11), 3247. 10.3390/nu12113247 33114073 PMC7690671

[B14] Duran-Pinedo A. E. Paster B. Teles R. Frias-Lopez J. (2011). Correlation network analysis applied to complex biofilm communities. PLoS ONE 6 (12), 28438. 10.1371/journal.pone.0028438 PMC3233593 22163302

[B15] Dvorak P. Hlavac V. Mohelnikova-Duchonova B. Liska V. Martin P. Pavel S. (2017). Downregulation of ABC transporters in non-neoplastic tissues confers better prognosis for pancreatic and colorectal cancer patients. J. Cancer 8 (11), 1959–1971. 10.7150/jca.19364 28819395 PMC5559956

[B16] Flint H. J. Scott K. P. S. H. D. Duncan S. H. Louis P. (2012). Petra louis, and evelyne foranomicrobial degradation of complex carbohydrates in the gut. Gut Microbes 3 (4), 289–306. 10.4161/gmic.19897 22572875 PMC3463488

[B17] Flores-BautistaHernandez-Guerrero E. Huerta-Saquero A. Tenorio-Salgado S. Rivera-Gomez N. Alba R. Jose Antonio I. Perez-Rueda E. (2020). Deciphering the functional diversity of DNA-binding transcription factors in Bacteria and Archaea organisms. PLOS ONE 15 (8), 0237135. 10.1371/journal.pone.0237135 PMC7446807 32822422

[B18] Galán-Vásquez E. Perez-Rueda E. (2019). Identification of modules with similar gene regulation and metabolic functions based on Co-expression data. Front. Mol. Biosci. 6 (139), 139. 10.3389/fmolb.2019.00139 31921888 PMC6929668

[B19] Garrido D. Ruiz-Moyano S. Lemay D. G. Sela D. A. Bruce German J. Mills D. A. (2015). Comparative transcriptomics reveals key differences in the response to milk oligosaccharides of infant gut-associated bifidobacteria. Sci. Rep. 5 (1), 13517. 10.1038/srep13517 26337101 PMC4559671

[B20] Garrido D. Ruiz-Moyano S. Kirmiz N. Davis J. C. Totten S. M. Lemay D. G. (2016). A novel gene cluster allows preferential utilization of fucosylated milk oligosaccharides in bifidobacterium longum subsp. longum SC596. Sci. Rep. 6 (1), 35045. 10.1038/srep35045 27756904 PMC5069460

[B21] Gibson G. R. McCartney A. L. Rastall R. A. (2005). Prebiotics and resistance to gastrointestinal infections. Br. J. Nutr. 93 (S1), S31–S34. 10.1079/BJN20041343 15877892

[B22] Horvath S. (2011). Weighted network Analysis|Applications in genomics and systems biology. 1st ed. New York, NY: Springer New York. 10.1007/978-1-4419-8819-5

[B23] Jha R. K. Udupa S. Rai A. K. Rani P. Singh P. R. Govind S. (2020). Conditional down-regulation of GreA impacts expression of RRNA and transcription factors, affecting Mycobacterium smegmatis survival. Sci. Rep. 10 (1), 5802. 10.1038/s41598-020-62703-7 32242064 PMC7118132

[B24] Jia R. Zhao H. Jia M. (2020). Identification of Co-expression modules and potential biomarkers of breast cancer by WGCNA. Gene 750, 144757. 10.1016/j.gene.2020.144757 32387385

[B25] Junker B. H. Falk S. (2008). “Analysis of biological networks,” Wiley series on bioinformatics: Computational techniques and engineering. Bjrn H. Junker Falk S. (Hoboken, NJ, USA: John Wiley and Sons). 10.1002/9780470253489

[B26] Kanehisa M. Sato Y. Furumichi M. Morishima K. Tanabe M. (2019). New approach for understanding genome variations in KEGG. Nucleic Acids Res. 47 (1), D590–95. 10.1093/nar/gky962 30321428 PMC6324070

[B27] Kanehisa M. Goto S. (2000). Kegg: Kyoto Encyclopedia of genes and genomes. Nucleic Acids Res. 28 (1), 27–30. 10.1093/nar/28.1.27 10592173 PMC102409

[B28] Khoroshkin M. S. Leyn S. A. Van Sinderen D. Rodionov D. A. (2016). Transcriptional regulation of carbohydrate utilization pathways in the bifidobacterium genus. Front. Microbiol. 7 (1), 120. 10.3389/fmicb.2016.00120 26903998 PMC4746261

[B29] Kitaoka M. (2012). Bifidobacterial enzymes involved in the metabolism of human milk oligosaccharides. Adv. Nutr. 3 (3), 422S–429S. 10.3945/an.111.001420 22585921 PMC3649479

[B30] Kukurba K. R. Montgomery. S. B. (2015). RNA sequencing and analysis. Cold Spring Harb. Protoc. 2015 (11), 951–969. 10.1101/pdb.top084970 25870306 PMC4863231

[B31] Langfelder P. Horvath S. (2008). Wgcna: An R package for weighted correlation network analysis. BMC Bioinforma. 9 (1), 559. 10.1186/1471-2105-9-559 PMC2631488 19114008

[B32] Lawson M. A. E. O’Neill I. J. Kujawska M. Sree Gowrinadh J. Wijeyesekera A. Zak F. Chalklen L. (2020). Breast milk-derived human milk oligosaccharides promote bifidobacterium interactions within a single ecosystem. ISME J. 14 (2), 635–648. 10.1038/s41396-019-0553-2 31740752 PMC6976680

[B33] Lewis M. (2005). The lac repressor. Comptes Rendus Biol. 328 (6), 521–548. 10.1016/j.crvi.2005.04.004 15950160

[B34] Liao Y. Wang Y. Cheng M. Huang C. Fan X. (2020). Weighted gene coexpression network analysis of features that control cancer stem cells reveals prognostic biomarkers in lung adenocarcinoma. Front. Genet. 11, 311. 10.3389/fgene.2020.00311 32391047 PMC7192063

[B35] Liu Z. Li L. Wang Q. Ahmed Sadiq F. Lee Y. Zhao J. (2021). Transcriptome analysis reveals the genes involved in bifidobacterium longum FGSZY16M3 biofilm formation. Microorganisms 9 (2), 385. 10.3390/microorganisms9020385 33672820 PMC7917626

[B36] Markowiak P. Śliżewska K. (2017). Effects of probiotics, prebiotics, and synbiotics on human health. Nutrients 9 (9), 1021. 10.3390/nu9091021 28914794 PMC5622781

[B37] Masi A. C. Stewart C. J. (2022). Untangling human milk oligosaccharides and infant gut microbiome. IScience 25 (1), 103542. 10.1016/j.isci.2021.103542 34950861 PMC8671521

[B38] Ojima M. N. Jiang L. Arzamasov A. A. Yoshida K. Odamaki T. Xiao J. (2022). Priority effects shape the structure of infant-type bifidobacterium communities on human milk oligosaccharides. ISME J. 16, 2265–2279. 10.1038/s41396-022-01270-3 35768643 PMC9381805

[B39] Ozsolak F. Milos P. M. (2011). RNA sequencing: Advances, challenges and opportunities. Nat. Rev. Genet. 12 (2), 87–98. 10.1038/nrg2934 21191423 PMC3031867

[B40] Ramos J. L. Martínez-Bueno M. Antonio J. Teran W. Watanabe K. Zhang X. (2005). Molina-henares, wilson terán, kazuya watanabe, xiaodong zhang, maría trinidad gallegos, richard brennan, and raquel tobesthe TetR family of transcriptional repressors. Microbiol. Mol. Biol. Rev. 69 (2), 326–356. 10.1128/MMBR.69.2.326-356.2005 15944459 PMC1197418

[B41] Ravcheev D. A. Khoroshkin M. S. Laikova Olga V Tsoy, Natalia V Sernova S. A. Petrova A. B Rakhmaninova P. S Novichkov M. S Gelfand O. N. Rodionov D. A. Sernova N. V. Petrova S. A. (2014). Comparative genomics and evolution of regulons of the LacI-family transcription factors. Front. Microbiol. 5, 294. 10.3389/fmicb.2014.00294 24966856 PMC4052901

[B42] Rezaei Z. Ranjbaran J. Safarpour H. Nomiri S. Salmani F. Chamani E. (2022). Identification of early diagnostic biomarkers via WGCNA in gastric cancer. Biomed. Pharmacother. 145, 112477. 10.1016/j.biopha.2021.112477 34864309

[B43] Sakanaka M. Gotoh A. Yoshida K. Odamaki T. Koguchi H. Xiao J.-z. (2020). Varied pathways of infant gut-associated bifidobacterium to assimilate human milk oligosaccharides: Prevalence of the gene set and its correlation with bifidobacteria-rich microbiota formation. Nutrients 12 (1), 71. 10.3390/nu12010071 PMC7019425 31888048

[B44] Sakanaka M. Hansen M. E. Gotoh A. Katoh T. Yoshida K. Odamaki T. (2019). Evolutionary adaptation in fucosyllactose uptake systems supports bifidobacteria-infant symbiosis. Sci. Adv. 5 (8), eaaw7696–16. 10.1126/sciadv.aaw7696 31489370 PMC6713505

[B45] Schmid A. K. (2018). “Conserved principles of transcriptional networks controlling metabolic flexibility in archaea,” Emerg. Top. Life Sci. Robinson Nicholas P., 2, 659–669. 10.1042/ETLS20180036 4 33525832 PMC7289023

[B46] Sela D. A. Chapman J. Adeuya A. Kim J. H. Chen F. Whitehead T. R. (2008). The genome sequence of bifidobacterium longum subsp. infantis reveals adaptations for milk utilization within the infant microbiome. Proc. Natl. Acad. Sci. 105 (48), 18964–18969. 10.1073/pnas.0809584105 19033196 PMC2596198

[B47] Shokryazdan P. Faseleh Jahromi M. Navidshad B. Juan Boo L. (2017). Effects of prebiotics on immune system and cytokine expression. Med. Microbiol. Immunol. 206 (1), 1–9. 10.1007/s00430-016-0481-y 27704207

[B48] Stuart J. M. Segal E. Koller D. Kim S. K. (2003). A gene-coexpression network for global discovery of conserved genetic modules. Science 302 (5643), 249–255. 10.1126/science.1087447 12934013

[B49] Sun Q. Zhao H. Zhang C. Hu T. Wu J. Lin X. (2017). Gene Co-expression network reveals shared modules predictive of stage and grade in serous ovarian cancers. Oncotarget 8 (26), 42983–42996. 10.18632/oncotarget.17785 28562334 PMC5522121

[B50] Thomson P. Medina D. A. Garrido D. (2018). Human milk oligosaccharides and infant gut bifidobacteria: Molecular strategies for their utilization. Food Microbiol. 75, 37–46. 10.1016/j.fm.2017.09.001 30056961

[B51] Tsevelkhoroloo M. Shim S. H. Lee C.-R. Hong S.-K. Young-Soo H. (2021). LacI-family transcriptional regulator DagR acts as a repressor of the agarolytic pathway genes in streptomyces coelicolor A3(2). Front. Microbiol. 12, 658657. 10.3389/fmicb.2021.658657 33889146 PMC8055832

[B52] Tsukuda N. Yahagi K. Hara T. Watanabe Y. Matsumoto H. Mori H. (2021). Key bacterial taxa and metabolic pathways affecting gut short-chain fatty acid profiles in early life. ISME J. 15 (9), 2574–2590. 10.1038/s41396-021-00937-7 33723382 PMC8397723

[B53] Turroni F. Milani C. Ventura M. Douwe van S. (2022). The human gut microbiota during the initial stages of life: Insights from bifidobacteria. Curr. Opin. Biotechnol. 73, 81–87. 10.1016/j.copbio.2021.07.012 34333445

[B54] Vernocchi P. Gili T. Federica C. Federica Del C. Conta G. Miccheli A. (2020). Network analysis of gut microbiome and metabolome to discover microbiota-linked biomarkers in patients affected by non-small cell lung cancer. Int. J. Mol. Sci. 21 (22), 8730. 10.3390/ijms21228730 33227982 PMC7699235

[B55] Walsh C. Lane J. A. Douwe van S. RitaHickey M. (2020). Human milk oligosaccharides: Shaping the infant gut microbiota and supporting health. J. Funct. Foods 72, 104074. 10.1016/j.jff.2020.104074 32834834 PMC7332462

[B56] Zabel B. E. Svetlana GerdesEvans K. C. Evans K. C. Nedveck D. Singles S. K. Volk B. (2020). Strain-specific strategies of 2'-fucosyllactose, 3-fucosyllactose, and difucosyllactose assimilation by Bifidobacterium longum subsp. infantis Bi-26 and ATCC 15697. Sci. Rep. 10 (1), 15919. 10.1038/s41598-020-72792-z 32985563 PMC7522266

[B57] Zhang B. Li L.-Q. Liu F. Wu J.-Y. (2022). Human milk oligosaccharides and infant gut microbiota: Molecular structures, utilization strategies and immune function. Carbohydr. Polym. 276, 118738. 10.1016/j.carbpol.2021.118738 34823774

[B58] Zhang B. Horvath S. (2005). A general framework for weighted gene Co-expression network analysis. Stat. Appl. Genet. Mol. Biol. 4 (1), 17. 10.2202/1544-6115.1128 16646834

[B59] Zheng X. Su Y. Chen Y. Huang H. Shen Q. (2018). Global transcriptional responses of denitrifying bacteria to functionalized single-walled carbon nanotubes revealed by weighted gene-coexpression network analysis. Sci. Total Environ. 613–614, 1240–1249. 10.1016/j.scitotenv.2017.09.193 28958131

[B60] Zúñiga M. Monedero V. Yebra M. J. (2018). Utilization of host-derived glycans by intestinal lactobacillus and bifidobacterium species. Front. Microbiol. 9, 1917. 10.3389/fmicb.2018.01917 30177920 PMC6109692

