# Supplementary figures and images for: Structure of co-expression networks of Bifidobacterium species in response to human milk oligosaccharides
Source: Front Mol Biosci. 2023 Jan 26;10:1040721. doi: 10.3389/fmolb.2023.1040721 (PMC9908966; doi:10.3389/fmolb.2023.1040721)

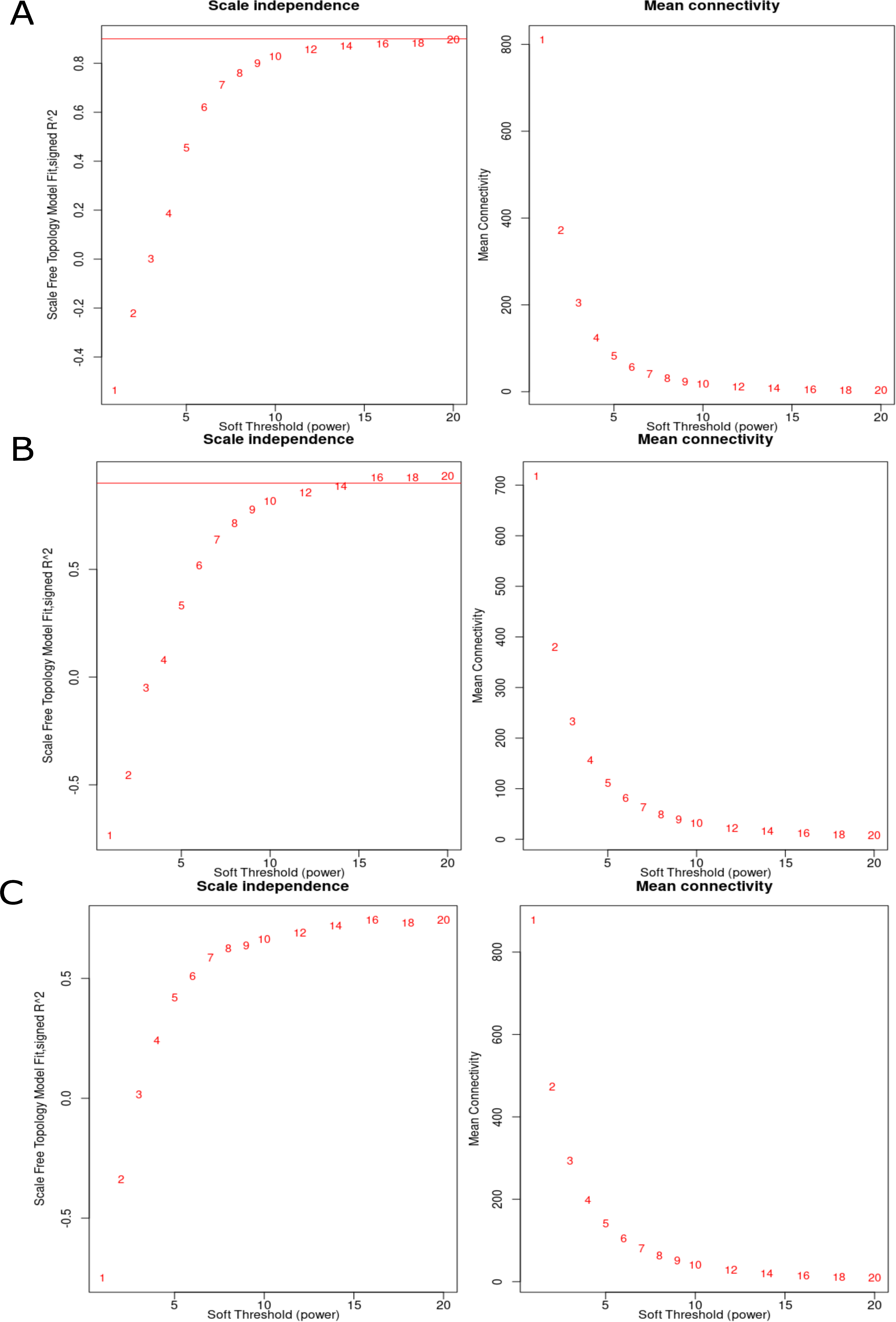

Supplement: Supplementary file 1 [file Image3.TIFF]

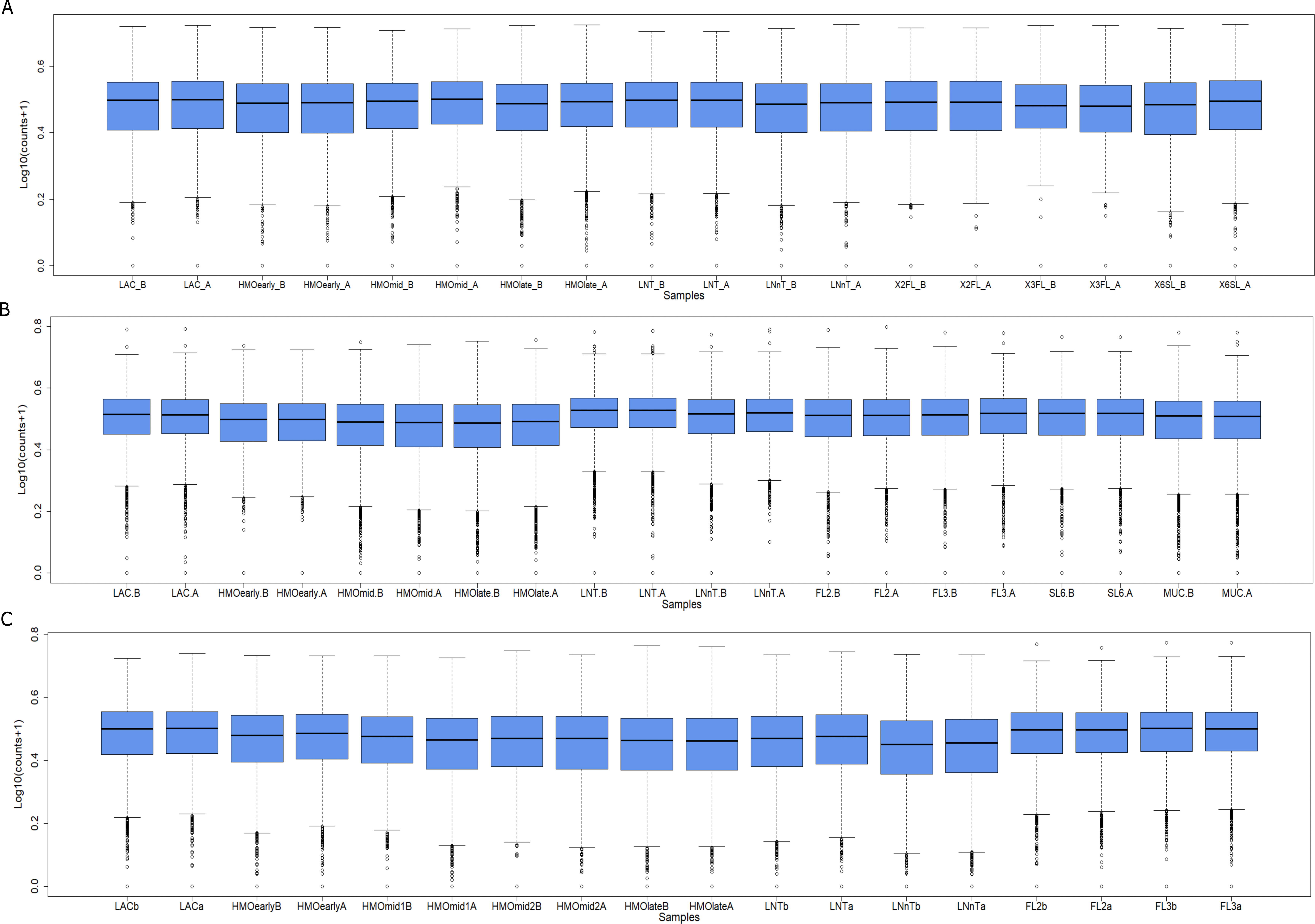

Supplement: Supplementary file 2 [file Image1.TIFF]

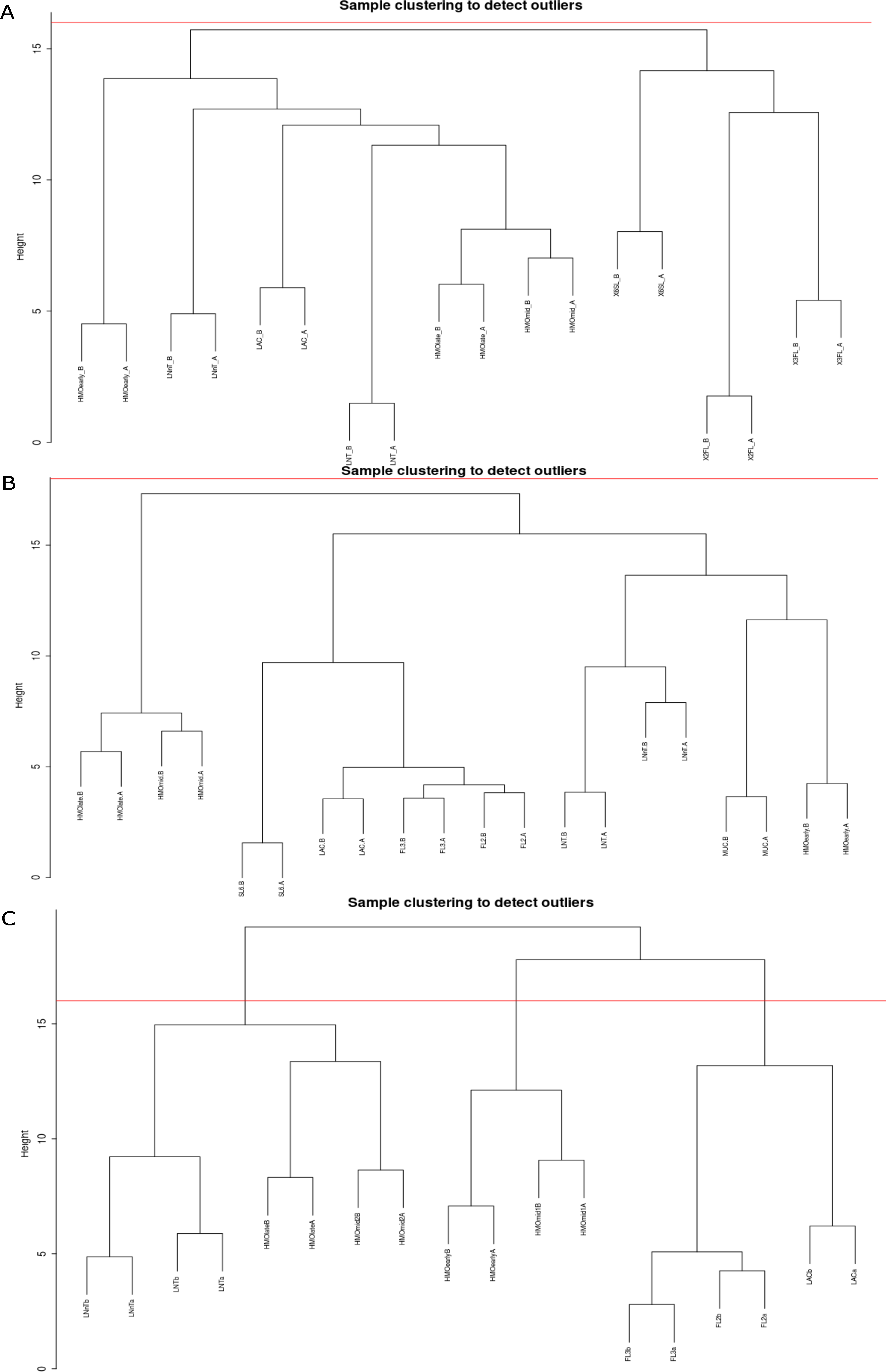

Supplement: Supplementary file 4 [file Image2.TIFF]

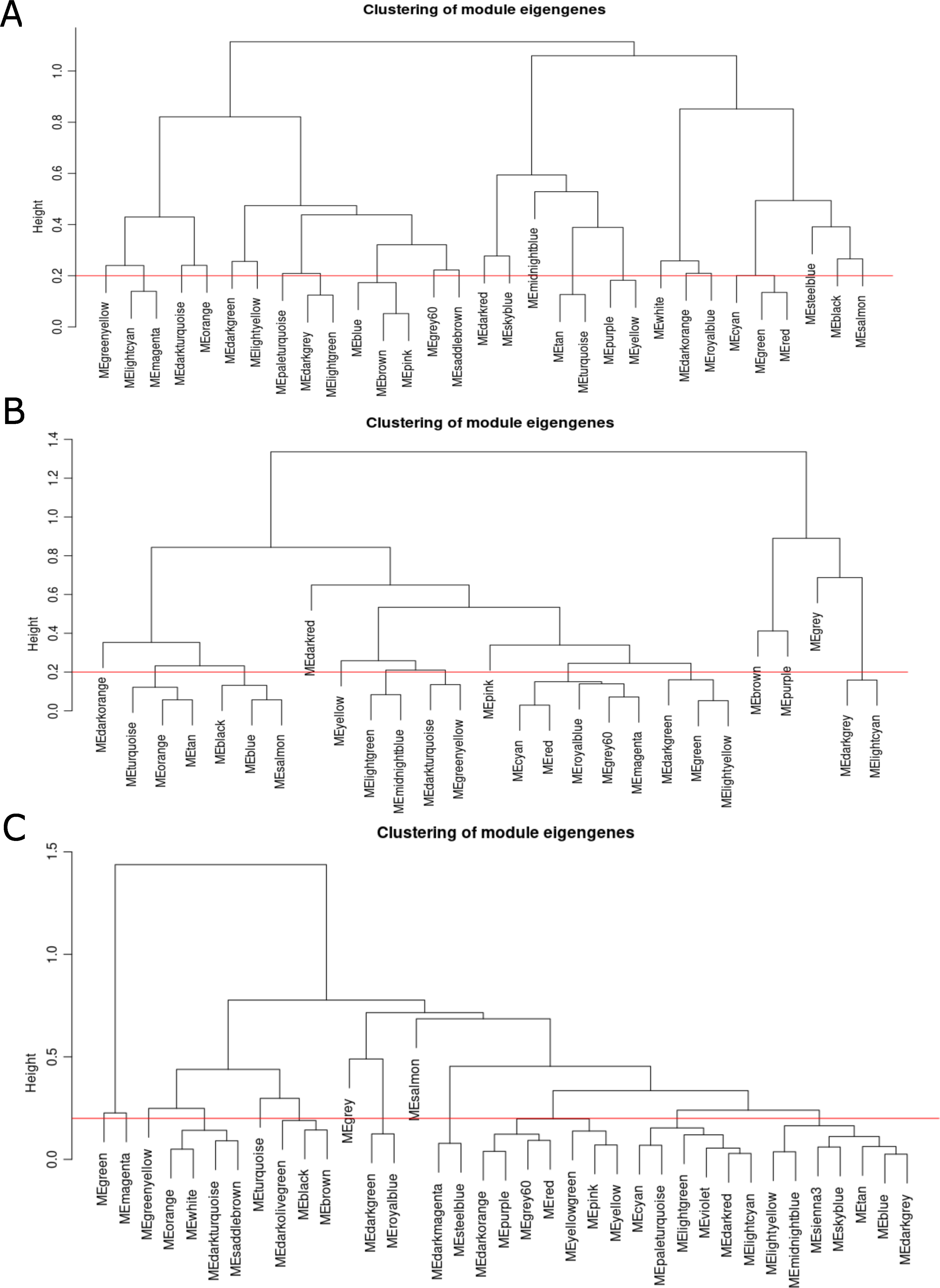

Supplement: Supplementary file 5 [file Image4.TIFF]
