# Supplementary material for: Structure of co-expression networks of Bifidobacterium species in response to human milk oligosaccharides
Source: Front Mol Biosci. 2023 Jan 26;10:1040721. doi: 10.3389/fmolb.2023.1040721 (PMC9908966; doi:10.3389/fmolb.2023.1040721)
Supplement: Supplementary file 3 [file Table1.DOCX]

Structure of Co-expression Networks of Bifidobacterium species in respond to Human Milk Oligosaccharides

**Kevin J. González-Morelo^1*^, Edgardo Galán-Vásquez^2^, Felipe Melis^1^, Ernesto Pérez-Rueda^1,2^, Daniel Garrido^1^**

^1^Department of Chemical and Bioprocess Engineering, School of Engineering, Pontificia Universidad Católica de Chile, Santiago, Chile.

^2^Department of Computer Systems Engineering and Automation, Instituto de Investigaciones en Matemáticas Aplicadas y en Sistemas, Ciudad Universitaria, Universidad Nacional Autónoma de México, Ciudad de México, México.

^3^Instituto de Investigaciones en Matemáticas Aplicadas y en Sistemas, Universidad Nacional Autónoma de México, Unidad Académica Yucatán, Mérida, México.

Supplementary Material

# Supplementary Tables

**Table S1. RNA-seq samples from different *Bifidobacterium* strains growth in various substrates.**

| ***Bifidobacterium longum* subsp. *infantis* ATCC 15697** | ***Bifidobacterium bifidum* SC555** | **B. longum subsp. longum SC596** |
| --- | --- | --- |
| GSM1419252,  lactose treatment, replicate A | GSM1425041,  lactose treatment, replicate A | GSM2338941,  lactose treatment, replicate A |
| GSM1419253,  lactose treatment, replicate B | GSM142504,  lactose treatment, replicate B | GSM2338942,  lactose treatment, replicate B |
| GSM1419254,  pooled HMO, early time point, replicate A | GSM142504,  pooled HMO, early time point, replicate A | GSM2338943, pooled HMO, early time point, replicate A |
| GSM1419255,  pooled HMO, early time point, replicate B | GSM1425044,  pooled HMO, early time point, replicate B | GSM2338944,  pooled HMO, early time point, replicate B |
| GSM1419256,  pooled HMO, mid-time point, replicate A | GSM1425045,  pooled HMO, mid-time point, replicate A | GSM2338945,  pooled HMO, mid1 time point, replicate A |
| GSM1419257,  pooled HMO, mid-time point, replicate B | GSM1425046,  pooled HMO, mid-time point, replicate B | GSM2338946,  pooled HMO, mid1 time point, replicate B |
| GSM1419258,  pooled HMO, late time point, replicate A | GSM1425047,  pooled HMO, late time point, replicate A | GSM2338947,  pooled HMO, mid2 time point, replicate A |
| GSM1419259,  pooled HMO, late time point, replicate B | GSM1425048,  pooled HMO, late time point, replicate B | GSM2338948,  pooled HMO, mid2 time point, replicate B |
| GSM1419260,  lacto-*N*-tetraose treatment, replicate A | GSM1425049,  lacto-*N*-tetraose treatment, replicate A | GSM2338949,  pooled HMO, late time point, replicate A |
| GSM1419261,  lacto-*N*-tetraose treatment, replicate B | GSM1425050,  lacto-*N*-tetraose treatment, replicate B | GSM2338950,  pooled HMO, late time point, replicate B |
| GSM1419262,  lacto-*N*-neotetraose treatment, replicate A | GSM1425051,  lacto-*N*-neotetraose treatment, replicate A | GSM2338951,  lacto-N-tetraose treatment, replicate A |
| GSM1419263,  lacto-*N*-neotetraose treatment, replicate B | GSM1425052,  lacto-*N*-neotetraose treatment, replicate B | GSM2338952,  lacto-*N*-tetraose treatment, replicate B |
| GSM1419264,  2’fucosyllactose treatment, replicate A | GSM1425053,  2’fucosyllactose treatment, replicate A | GSM2338953,  lacto-*N*-neotetraose treatment, replicate A |
| GSM1419265,  2’fucosyllactose treatment, replicate B | GSM1425054,  2’fucosyllactose treatment, replicate B | GSM2338954,  lacto-*N*-neotetraose treatment, replicate B |
| GSM1419266,  3-fucosyllactose treatment, replicate A | GSM1425055,  3-fucosyllactose treatment, replicate A | GSM2338955,  2’fucosyllactose treatment, replicate A |
| GSM1419267,  3-fucosyllactose treatment, replicate B | GSM1425056,  3-fucosyllactose treatment, replicate B | GSM2338956,  2’fucosyllactose treatment, replicate B |
| GSM1419268,  6’sialyllactose treatment, replicate A | GSM1425057,  6’sialyllactose treatment, replicate A | GSM2338957,  3-fucosyllactose treatment, replicate A |
| GSM1419269,  6’sialyllactose treatment, replicate B | GSM1425058,  6’sialyllactose treatment, replicate B | GSM2338958,  3-fucosyllactose treatment, replicate B |
|  | GSM1425059,  mucin treatment, replicate A |  |
|  | GSM1425060,  mucin treatment, replicate B |  |

**Table S2. Genes in module Greenyellow from B. *infantis*.***

| **Locus tag** | **Module** | **Annotation** |
| --- | --- | --- |
| Blon_0006 | greenyellow | DNA gyrase, B subunit |
| Blon_0017 | greenyellow | hypothetical protein |
| Blon_0018 | greenyellow | hypothetical protein |
| Blon_0020 | greenyellow | phosphonate ABC transporter, inner membrane subunit |
| Blon_0062 | greenyellow | conserved hypothetical protein |
| Blon_0070 | greenyellow | glycogen/starch/alpha-glucan phosphorylase |
| Blon_0077 | greenyellow | protein kinase |
| Blon_0078 | greenyellow | protein kinase |
| Blon_0079 | greenyellow | Peptidoglycan glycosyltransferase |
| Blon_0080 | greenyellow | cell cycle protein |
| Blon_0100 | greenyellow | #N/D |
| Blon_0117 | greenyellow | peptidase M48, Ste24p |
| Blon_0122 | greenyellow | hypothetical protein |
| Blon_0128 | greenyellow | Sucrose phosphorylase |
| Blon_0129 | greenyellow | major facilitator superfamily MFS_1 |
| Blon_0135 | greenyellow | zinc finger, CHY domain protein |
| Blon_0152 | greenyellow | type II secretion system protein E |
| Blon_0153 | greenyellow | conserved hypothetical protein |
| Blon_0155 | greenyellow | conserved hypothetical protein |
| Blon_0177 | greenyellow | Phosphotransferase system, phosphocarrier protein HPr |
| Blon_0179 | greenyellow | conserved hypothetical protein |
| Blon_0180 | greenyellow | peptidase U34, dipeptidase |
| Blon_0188 | greenyellow | Aldose 1-epimerase |
| Blon_0189 | greenyellow | protein of unknown function DUF805 |
| Blon_0204 | greenyellow | conserved hypothetical membrane protein |
| Blon_0205 | greenyellow | ABC-2 type transporter |
| Blon_0207 | greenyellow | 6-phosphogluconate dehydrogenase, decarboxylating |
| Blon_0210 | greenyellow | glucose-6-phosphate 1-dehydrogenase |
| Blon_0244 | greenyellow | Signal transduction histidine kinase-like protein |
| Blon_0247 | greenyellow | major facilitator superfamily MFS_1 |
| Blon_0248 | greenyellow | Alpha-L-fucosidase |
| Blon_0257 | greenyellow | conserved hypothetical protein |
| Blon_0258 | greenyellow | glutamyl-tRNA(Gln) amidotransferase, C subunit |
| Blon_0269 | greenyellow | #N/D |
| Blon_0287 | greenyellow | hypothetical protein |
| Blon_0300 | greenyellow | hypothetical protein |
| Blon_0328 | greenyellow | transcriptional regulator, TetR family |
| Blon_0340 | greenyellow | LacI-family transcriptional regulator |
| Blon_0341 | greenyellow | binding-protein-dependent transport systems inner membrane component |
| Blon_0342 | greenyellow | binding-protein-dependent transport systems inner membrane component |
| Blon_0343 | greenyellow | extracellular solute-binding protein, family 1 |
| Blon_0347 | greenyellow | hypothetical protein |
| Blon_0353 | greenyellow | 4-diphosphocytidyl-2C-methyl-D-erythritol synthase |
| Blon_0354 | greenyellow | Pyroglutamyl-peptidase I |
| Blon_0376 | greenyellow | binding-protein-dependent transport systems inner membrane component |
| Blon_0377 | greenyellow | binding-protein-dependent transport systems inner membrane component |
| Blon_0378 | greenyellow | hypothetical protein |
| Blon_0386 | greenyellow | riboflavin biosynthesis protein RibD |
| Blon_0387 | greenyellow | riboflavin synthase, alpha subunit |
| Blon_0403 | greenyellow | conserved hypothetical protein |
| Blon_0415 | greenyellow | Methicillin resistance protein |
| Blon_0417 | greenyellow | Glucose-6-phosphate isomerase |
| Blon_0425 | greenyellow | major facilitator superfamily MFS_1 |
| Blon_0436 | greenyellow | ABC transporter related |
| Blon_0446 | greenyellow | conserved hypothetical protein |
| Blon_0447 | greenyellow | DNA primase, small subunit |
| Blon_0450 | greenyellow | hypothetical protein |
| Blon_0451 | greenyellow | hypothetical protein |
| Blon_0452 | greenyellow | #N/D |
| Blon_0453 | greenyellow | hypothetical protein |
| Blon_0458 | greenyellow | phage integrase family protein |
| Blon_0463 | greenyellow | two component transcriptional regulator, LuxR family |
| Blon_0474 | greenyellow | ABC transporter related |
| Blon_0475 | greenyellow | ABC transporter related |
| Blon_0480 | greenyellow | ABC transporter related |
| Blon_0489 | greenyellow | phage integrase family protein |
| Blon_0509 | greenyellow | integral membrane sensor signal transduction histidine kinase |
| Blon_0510 | greenyellow | two component transcriptional regulator, winged helix family |
| Blon_0511 | greenyellow | hypothetical protein |
| Blon_0512 | greenyellow | conserved hypothetical protein |
| Blon_0513 | greenyellow | Peptidoglycan-binding domain 1 protein |
| Blon_0521 | greenyellow | Lanthionine synthetase C family protein |
| Blon_0523 | greenyellow | ABC-2 type transporter |
| Blon_0544 | greenyellow | ABC transporter related |
| Blon_0545 | greenyellow | conserved hypothetical transmembrane protein with unknown function |
| Blon_0563 | greenyellow | transposase IS116/IS110/IS902 family protein |
| Blon_0567 | greenyellow | 2,5-didehydrogluconate reductase |
| Blon_0574 | greenyellow | ribosomal protein L31 |
| Blon_0593 | greenyellow | methionine aminopeptidase, type I |
| Blon_0595 | greenyellow | 2,3,4,5-tetrahydropyridine-2-carboxylate N-succinyltransferase |
| Blon_0608 | greenyellow | NAD(P)H dehydrogenase (quinone) |
| Blon_0610 | greenyellow | Exonuclease VII, small subunit |
| Blon_0615 | greenyellow | Resolvase, N-terminal domain protein |
| Blon_0617 | greenyellow | glutamate--cysteine ligase, GCS2 |
| Blon_0628 | greenyellow | membrane protein |
| Blon_0629 | greenyellow | glycosyl transferase, group 1 |
| Blon_0630 | greenyellow | conserved hypothetical protein |
| Blon_0631 | greenyellow | membrane lipoprotein lipid attachment site |
| Blon_0632 | greenyellow | conserved hypothetical protein |
| Blon_0638 | greenyellow | Purine nucleosidase |
| Blon_0639 | greenyellow | major facilitator superfamily MFS_1 |
| Blon_0640 | greenyellow | PfkB domain protein |
| Blon_0655 | greenyellow | ABC-type spermidine/putrescine transport system permease component II-like protein |
| Blon_0659 | greenyellow | IS3 family transposase |
| Blon_0660 | greenyellow | hypothetical protein |
| Blon_0679 | greenyellow | histone family protein DNA-binding protein |
| Blon_0680 | greenyellow | narrowly conserved hypothetical membrane protein |
| Blon_0685 | greenyellow | cell surface elastin binding protein EbpS |
| Blon_0704 | greenyellow | protein of unknown function DUF349 |
| Blon_0720 | greenyellow | conserved hypothetical protein |
| Blon_0737 | greenyellow | carbamoyl-phosphate synthase, small subunit |
| Blon_0738 | greenyellow | carbamoyl-phosphate synthase, large subunit |
| Blon_0745 | greenyellow | polar amino acid ABC transporter, inner membrane subunit |
| Blon_0746 | greenyellow | ABC transporter related |
| Blon_0778 | greenyellow | conserved hypothetical protein |
| Blon_0779 | greenyellow | 1-deoxy-D-xylulose 5-phosphate reductoisomerase |
| Blon_0783 | greenyellow | undecaprenyl diphosphate synthase |
| Blon_0785 | greenyellow | membrane lipoprotein lipid attachment site |
| Blon_0788 | greenyellow | oligosaccharide/H+ symporter, major facilitator superfamily (MFS) |
| Blon_0810 | greenyellow | UvrD/REP helicase |
| Blon_0814 | greenyellow | protein of unknown function DUF262 |
| Blon_0822 | greenyellow | acylphosphatase |
| Blon_0823 | greenyellow | Histidinol dehydrogenase |
| Blon_0833 | greenyellow | binding-protein-dependent transport systems inner membrane component |
| Blon_0834 | greenyellow | extracellular solute-binding protein, family 5 |
| Blon_0835 | greenyellow | ABC transporter related |
| Blon_0840 | greenyellow | L-lactate dehydrogenase |
| Blon_0853 | greenyellow | phospho-N-acetylmuramoyl-pentapeptide-transferase |
| Blon_0854 | greenyellow | UDP-N-acetylmuramoylalanine--D-glutamate ligase |
| Blon_0862 | greenyellow | ABC transporter related |
| Blon_0863 | greenyellow | ABC-2 type transporter |
| Blon_0868 | greenyellow | glycoside hydrolase, family 38 |
| Blon_0869 | greenyellow | glycoside hydrolase, family 38 |
| Blon_0876 | greenyellow | Uncharacterised conserved protein UCP028846 |
| Blon_0889 | greenyellow | conserved hypothetical protein |
| Blon_0892 | greenyellow | conserved hypothetical protein |
| Blon_0894 | greenyellow | RNA polymerase, sigma-24 subunit, ECF subfamily |
| Blon_0896 | greenyellow | Aldose 1-epimerase |
| Blon_0900 | greenyellow | glyceraldehyde-3-phosphate dehydrogenase, type I |
| Blon_0908 | greenyellow | putative transcriptional regulator |
| Blon_0918 | greenyellow | Prephenate dehydratase |
| Blon_0919 | greenyellow | Prephenate dehydrogenase |
| Blon_0922 | greenyellow | extracellular solute-binding protein, family 5 |
| Blon_0923 | greenyellow | binding-protein-dependent transport systems inner membrane component |
| Blon_0924 | greenyellow | binding-protein-dependent transport systems inner membrane component |
| Blon_0932 | greenyellow | aconitate hydratase 1 |
| Blon_0935 | greenyellow | protein of unknown function DUF262 |
| Blon_0936 | greenyellow | GCN5-related N-acetyltransferase |
| Blon_0937 | greenyellow | #N/D |
| Blon_0995 | greenyellow | ABC transporter related |
| Blon_0996 | greenyellow | conserved hypothetical protein |
| Blon_1007 | greenyellow | pyridoxamine 5'-phosphate oxidase-related, FMN-binding |
| Blon_1008 | greenyellow | conserved hypothetical protein |
| Blon_1026 | greenyellow | hypothetical protein |
| Blon_1027 | greenyellow | hypothetical protein |
| Blon_1028 | greenyellow | hypothetical protein |
| Blon_1029 | greenyellow | Methylase of polypeptide chain release factors-like protein |
| Blon_1030 | greenyellow | ABC transporter related |
| Blon_1036 | greenyellow | narrowly conserved hypothetical protein |
| Blon_1037 | greenyellow | conserved hypothetical protein |
| Blon_1038 | greenyellow | hypothetical protein |
| Blon_1042 | greenyellow | ABC transporter related |
| Blon_1058 | greenyellow | uridylate kinase |
| Blon_1059 | greenyellow | ribosome recycling factor |
| Blon_1060 | greenyellow | phosphatidate cytidylyltransferase |
| Blon_1095 | greenyellow | transcriptional regulator, Fis family |
| Blon_1096 | greenyellow | transketolase |
| Blon_1099 | greenyellow | hypothetical protein |
| Blon_1101 | greenyellow | hypothetical protein |
| Blon_1116 | greenyellow | phage major capsid protein, HK97 |
| Blon_1125 | greenyellow | hypothetical protein |
| Blon_1127 | greenyellow | hypothetical protein |
| Blon_1128 | greenyellow | pancreatic ribonuclease |
| Blon_1131 | greenyellow | hypothetical protein |
| Blon_1133 | greenyellow | hypothetical protein |
| Blon_1134 | greenyellow | hypothetical protein |
| Blon_1136 | greenyellow | hypothetical protein |
| Blon_1137 | greenyellow | hypothetical protein |
| Blon_1138 | greenyellow | hypothetical protein |
| Blon_1139 | greenyellow | hypothetical protein |
| Blon_1147 | greenyellow | hypothetical protein |
| Blon_1163 | greenyellow | conserved hypothetical protein |
| Blon_1164 | greenyellow | Phosphoribosylaminoimidazolecarboxamide formyltransferase |
| Blon_1169 | greenyellow | UTP--glucose-1-phosphate uridylyltransferase |
| Blon_1171 | greenyellow | hypothetical protein |
| Blon_1173 | greenyellow | narrowly conserved hypothetical protein |
| Blon_1179 | greenyellow | transposase, IS605 OrfB family |
| Blon_1180 | greenyellow | hypothetical protein |
| Blon_1190 | greenyellow | hypothetical protein |
| Blon_1196 | greenyellow | #N/D |
| Blon_1197 | greenyellow | adenine-specific methyltransferase EcoRI |
| Blon_1198 | greenyellow | hypothetical protein |
| Blon_1199 | greenyellow | hypothetical protein |
| Blon_1238 | greenyellow | transcriptional regulator, XRE family |
| Blon_1241 | greenyellow | transposase IS116/IS110/IS902 family protein |
| Blon_1276 | greenyellow | hypothetical protein |
| Blon_1279 | greenyellow | hypothetical protein |
| Blon_1281 | greenyellow | hypothetical protein |
| Blon_1282 | greenyellow | hypothetical protein |
| Blon_1283 | greenyellow | hypothetical protein |
| Blon_1288 | greenyellow | metallophosphoesterase |
| Blon_1292 | greenyellow | hypothetical protein |
| Blon_1293 | greenyellow | hypothetical protein |
| Blon_1299 | greenyellow | hypothetical protein |
| Blon_1302 | greenyellow | hypothetical protein |
| Blon_1304 | greenyellow | hypothetical protein |
| Blon_1305 | greenyellow | hypothetical protein |
| Blon_1345 | greenyellow | hypothetical protein |
| Blon_1347 | greenyellow | hypothetical protein |
| Blon_1348 | greenyellow | phage integrase family protein |
| Blon_1357 | greenyellow | ABC transporter related |
| Blon_1358 | greenyellow | protein of unknown function DUF214 |
| Blon_1365 | greenyellow | CDP-alcohol phosphatidyltransferase |
| Blon_1366 | greenyellow | ATP phosphoribosyltransferase |
| Blon_1367 | greenyellow | phosphoribosyl-ATP pyrophosphohydrolase |
| Blon_1380 | greenyellow | GCN5-related N-acetyltransferase |
| Blon_1383 | greenyellow | SSS sodium solute transporter superfamily |
| Blon_1385 | greenyellow | hypothetical protein |
| Blon_1393 | greenyellow | #N/D |
| Blon_1439 | greenyellow | aminotransferase, class I and II |
| Blon_1441 | greenyellow | HAD-superfamily hydrolase, subfamily IA, variant 3 |
| Blon_1446 | greenyellow | dihydroorotate dehydrogenase family protein |
| Blon_1448 | greenyellow | orotidine 5'-phosphate decarboxylase |
| Blon_1449 | greenyellow | amidohydrolase |
| Blon_1450 | greenyellow | aspartate transcarbamylase regulatory subunit |
| Blon_1454 | greenyellow | 5,10-methylenetetrahydrofolate reductase |
| Blon_1455 | greenyellow | 5-methyltetrahydropteroyltriglutamate--homocysteine S-methyltransferase |
| Blon_1457 | greenyellow | putative protein-L-isoaspartate methyltransferase |
| Blon_1465 | greenyellow | plasmid pRiA4b ORF-3 family protein |
| Blon_1491 | greenyellow | conserved hypothetical protein |
| Blon_1493 | greenyellow | hypothetical protein |
| Blon_1509 | greenyellow | hypothetical protein |
| Blon_1516 | greenyellow | transcription factor WhiB |
| Blon_1517 | greenyellow | hypothetical protein |
| Blon_1518 | greenyellow | conserved hypothetical protein |
| Blon_1519 | greenyellow | hypothetical protein |
| Blon_1526 | greenyellow | DNA-cytosine methyltransferase |
| Blon_1527 | greenyellow | #N/D |
| Blon_1528 | greenyellow | phage antirepressor protein |
| Blon_1533 | greenyellow | hypothetical protein |
| Blon_1535 | greenyellow | conserved hypothetical protein |
| Blon_1539 | greenyellow | hypothetical protein |
| Blon_1543 | greenyellow | hypothetical protein |
| Blon_1544 | greenyellow | hypothetical protein |
| Blon_1567 | greenyellow | transposase IS116/IS110/IS902 family protein |
| Blon_1580 | greenyellow | AMP-dependent synthetase and ligase |
| Blon_1582 | greenyellow | CBS domain containing protein |
| Blon_1583 | greenyellow | protein of unknown function UPF0054 |
| Blon_1592 | greenyellow | FeS assembly ATPase SufC |
| Blon_1593 | greenyellow | FeS assembly protein SufD |
| Blon_1594 | greenyellow | FeS assembly protein SufB |
| Blon_1595 | greenyellow | CTP synthase |
| Blon_1606 | greenyellow | RelB antitoxin |
| Blon_1630 | greenyellow | conserved hypothetical protein |
| Blon_1631 | greenyellow | ABC transporter related |
| Blon_1634 | greenyellow | protein of unknown function DUF47 |
| Blon_1652 | greenyellow | hypothetical protein |
| Blon_1659 | greenyellow | hypothetical protein |
| Blon_1669 | greenyellow | hypothetical protein |
| Blon_1670 | greenyellow | hypothetical protein |
| Blon_1671 | greenyellow | hypothetical protein |
| Blon_1674 | greenyellow | transcriptional regulator, TetR family |
| Blon_1686 | greenyellow | MATE efflux family protein |
| Blon_1690 | greenyellow | pyrroline-5-carboxylate reductase |
| Blon_1691 | greenyellow | proline iminopeptidase |
| Blon_1692 | greenyellow | integral membrane sensor signal transduction histidine kinase |
| Blon_1693 | greenyellow | two component transcriptional regulator, LuxR family |
| Blon_1696 | greenyellow | O-acetylhomoserine/O-acetylserine sulfhydrylase |
| Blon_1702 | greenyellow | succinate dehydrogenase and fumarate reductase iron-sulfur protein |
| Blon_1704 | greenyellow | conserved hypothetical protein 730 |
| Blon_1715 | greenyellow | formate acetyltransferase |
| Blon_1716 | greenyellow | conserved hypothetical protein |
| Blon_1719 | greenyellow | binding-protein-dependent transport systems inner membrane component |
| Blon_1720 | greenyellow | ABC transporter related |
| Blon_1721 | greenyellow | NLPA lipoprotein |
| Blon_1722 | greenyellow | Fructose-6-phosphate phosphoketolase |
| Blon_1723 | greenyellow | GMP synthase, large subunit |
| Blon_1730 | greenyellow | phosphate acetyltransferase |
| Blon_1731 | greenyellow | acetate kinase |
| Blon_1745 | greenyellow | Pyruvate kinase |
| Blon_1756 | greenyellow | periplasmic solute binding protein |
| Blon_1761 | greenyellow | 1,4-alpha-glucan branching enzyme |
| Blon_1781 | greenyellow | filamentation induced by cAMP protein Fic |
| Blon_1782 | greenyellow | protein of unknown function DUF450 |
| Blon_1786 | greenyellow | hypothetical protein |
| Blon_1797 | greenyellow | hypothetical protein |
| Blon_1799 | greenyellow | hypothetical protein |
| Blon_1808 | greenyellow | hypothetical protein |
| Blon_1810 | greenyellow | hypothetical protein |
| Blon_1814 | greenyellow | hypothetical protein |
| Blon_1832 | greenyellow | holin |
| Blon_1842 | greenyellow | protein of unknown function UPF0182 |
| Blon_1848 | greenyellow | threonine synthase |
| Blon_1857 | greenyellow | Signal transduction histidine kinase-like protein |
| Blon_1878 | greenyellow | acetylornithine and succinylornithine aminotransferase |
| Blon_1898 | greenyellow | transcriptional regulator, PadR-like family |
| Blon_1899 | greenyellow | HhH-GPD family protein |
| Blon_1903 | greenyellow | hypothetical protein |
| Blon_1906 | greenyellow | electron transport complex, RnfABCDGE type, D subunit |
| Blon_1910 | greenyellow | conserved hypothetical protein |
| Blon_1922 | greenyellow | translation elongation factor Tu |
| Blon_1941 | greenyellow | phosphoribosylformylglycinamidine synthase |
| Blon_1943 | greenyellow | conserved hypothetical protein |
| Blon_1965 | greenyellow | ribosomal protein L28 |
| Blon_1967 | greenyellow | ribosomal protein S14 |
| Blon_1968 | greenyellow | ribosomal protein L31 |
| Blon_1969 | greenyellow | ribosomal protein L36 |
| Blon_1970 | greenyellow | ribosomal protein L32 |
| Blon_1984 | greenyellow | protein of unknown function DUF214 |
| Blon_1985 | greenyellow | conserved hypothetical protein |
| Blon_1986 | greenyellow | ABC transporter related |
| Blon_1990 | greenyellow | hypothetical protein |
| Blon_1992 | greenyellow | putative transcriptional regulator, GntR family |
| Blon_1993 | greenyellow | SNO glutamine amidotransferase |
| Blon_1994 | greenyellow | pyridoxine biosynthesis protein |
| Blon_2006 | greenyellow | major facilitator superfamily MFS_1 |
| Blon_2030 | greenyellow | Peptide deformylase |
| Blon_2031 | greenyellow | phosphoglucosamine mutase |
| Blon_2058 | greenyellow | protein of unknown function DUF624 |
| Blon_2061 | greenyellow | extracellular solute-binding protein, family 1 |
| Blon_2062 | greenyellow | galactokinase |
| Blon_2063 | greenyellow | galactose-1-phosphate uridylyltransferase |
| Blon_2065 | greenyellow | Dihydroorotate oxidase |
| Blon_2069 | greenyellow | biotin/lipoate A/B protein ligase |
| Blon_2071 | greenyellow | Oligopeptidase B |
| Blon_2072 | greenyellow | narrowly conserved hypothetical protein |
| Blon_2078 | greenyellow | G5 domain protein |
| Blon_2104 | greenyellow | glycosyl transferase, family 14 |
| Blon_2113 | greenyellow | transposase IS116/IS110/IS902 family protein |
| Blon_2115 | greenyellow | hypothetical protein |
| Blon_2122 | greenyellow | metallophosphoesterase |
| Blon_2124 | greenyellow | protein of unknown function DUF245 domain protein |
| Blon_2125 | greenyellow | protein of unknown function DUF797 |
| Blon_2127 | greenyellow | protein of unknown function DUF245 domain protein |
| Blon_2128 | greenyellow | AAA ATPase, central domain protein |
| Blon_2129 | greenyellow | conserved hypothetical conserved transmembrane protein in the DedA family |
| Blon_2133 | greenyellow | methionyl-tRNA formyltransferase |
| Blon_2141 | greenyellow | Dihydrofolate reductase |
| Blon_2148 | greenyellow | putative phosphoserine aminotransferase |
| Blon_2149 | greenyellow | conserved hypothetical protein |
| Blon_2151 | greenyellow | phosphate uptake regulator, PhoU |
| Blon_2152 | greenyellow | phosphoglycerate mutase 1 family |
| Blon_2153 | greenyellow | putative exodeoxyribonuclease V |
| Blon_2164 | greenyellow | Tetratricopeptide TPR_2 repeat protein |
| Blon_2165 | greenyellow | conserved hypothetical protein |
| Blon_2172 | greenyellow | UDP-glucose--hexose-1-phosphate uridylyltransferase |
| Blon_2173 | greenyellow | aminoglycoside phosphotransferase |
| Blon_2174 | greenyellow | conserved hypothetical protein |
| Blon_2175 | greenyellow | binding-protein-dependent transport systems inner membrane component |
| Blon_2176 | greenyellow | binding-protein-dependent transport systems inner membrane component |
| Blon_2177 | greenyellow | extracellular solute-binding protein, family 1 |
| Blon_2183 | greenyellow | PTS system, glucose subfamily, IIA subunit |
| Blon_2184 | greenyellow | phosphoglucomutase, alpha-D-glucose phosphate-specific |
| Blon_2187 | greenyellow | transcriptional regulator, BadM/Rrf2 family |
| Blon_2201 | greenyellow | #N/D |
| Blon_2202 | greenyellow | extracellular solute-binding protein, family 1 |
| Blon_2203 | greenyellow | binding-protein-dependent transport systems inner membrane component |
| Blon_2204 | greenyellow | binding-protein-dependent transport systems inner membrane component |
| Blon_2268 | greenyellow | conserved hypothetical protein |
| Blon_2297 | greenyellow | aminotransferase, class I and II |
| Blon_2305 | greenyellow | RbsD or FucU transport |
| Blon_2306 | greenyellow | amidohydrolase 2 |
| Blon_2307 | greenyellow | major facilitator superfamily MFS_1 |
| Blon_2308 | greenyellow | short-chain dehydrogenase/reductase SDR |
| Blon_2309 | greenyellow | Mandelate racemase/muconate lactonizing enzyme, C-terminal domain protein |
| Blon_2328 | greenyellow | Polyphosphate kinase |
| Blon_2331 | greenyellow | sugar (Glycoside-Pentoside-Hexuronide) transporter |
| Blon_2334 | greenyellow | glycoside hydrolase family 2, TIM barrel |
| Blon_2354 | greenyellow | extracellular solute-binding protein, family 1 |
| Blon_2355 | greenyellow | glycoside hydrolase, family 20 |
| Blon_2359 | greenyellow | binding-protein-dependent transport systems inner membrane component |
| Blon_2360 | greenyellow | binding-protein-dependent transport systems inner membrane component |
| Blon_2363 | greenyellow | uracil phosphoribosyltransferase |
| Blon_2364 | greenyellow | protein of unknown function DUF163 |
| Blon_2375 | greenyellow | fumarylacetoacetate (FAA) hydrolase |
| Blon_2377 | greenyellow | narrowly conserved hypothetical protein |
| Blon_2378 | greenyellow | binding-protein-dependent transport systems inner membrane component |
| Blon_2379 | greenyellow | binding-protein-dependent transport systems inner membrane component |
| Blon_2380 | greenyellow | extracellular solute-binding protein, family 1 |
| Blon_2384 | greenyellow | hypothetical protein |
| Blon_2386 | greenyellow | ABC transporter related |
| Blon_2397 | greenyellow | K+ potassium transporter |
| Blon_2399 | greenyellow | Esterase/lipase-like protein |
| Blon_2400 | greenyellow | sugar transporter |
| Blon_2405 | greenyellow | #N/D |
| Blon_2427 | greenyellow | conserved hypothetical protein |
| Blon_2453 | greenyellow | alpha amylase, catalytic region |
| Blon_2455 | greenyellow | conserved hypothetical protein |
| Blon_2456 | greenyellow | binding-protein-dependent transport systems inner membrane component |
| Blon_2457 | greenyellow | binding-protein-dependent transport systems inner membrane component |
| Blon_2458 | greenyellow | extracellular solute-binding protein, family 1 |
| Blon_2460 | greenyellow | glycoside hydrolase, clan GH-D |
| Blon_2463 | greenyellow | phospholipase/Carboxylesterase |
| Blon_2465 | greenyellow | deoxycytidine triphosphate deaminase |
| Blon_2467 | greenyellow | sodium:dicarboxylate symporter |
| Blon_2469 | greenyellow | ATPase, P-type (transporting), HAD superfamily, subfamily IC |
| Blon_2471 | greenyellow | PTS system, N-acetylglucosamine-specific IIBC subunit |
| Blon_2472 | greenyellow | major facilitator superfamily MFS_1 |
| Blon_2473 | greenyellow | RNA methyltransferase, TrmH family, group 3 |
| Blon_2474 | greenyellow | narrowly conserved hypothetical protein |
| Blon_2475 | greenyellow | ABC transporter related |
| Blon_2483 | greenyellow | G5 domain protein |
| Blon_2484 | greenyellow | dimethyladenosine transferase |
| Blon_2485 | greenyellow | 4-(cytidine 5'-diphospho)-2-C-methyl-D-erythritol kinase |
| Blon_2488 | greenyellow | NUDIX hydrolase |
| Blon_R0004 | greenyellow | #N/D |
| Blon_R0025 | greenyellow | #N/D |
| Blon_R0047 | greenyellow | #N/D |
| Blon_R0050 | greenyellow | #N/D |
| Blon_R0052 | greenyellow | #N/D |
| Blon_R0054 | greenyellow | #N/D |
| Blon_R0055 | greenyellow | #N/D |
| Blon_R0057 | greenyellow | #N/D |
| Blon_R0071 | greenyellow | #N/D |
| Blon_R0077 | greenyellow | #N/D |
| Blon_R0088 | greenyellow | #N/D |
| Blon_R0091 | greenyellow | #N/D |

*** Shaded rows refer to genes associated with HMO consumption according to Garrido et al., (2015)**

**Table S3. Genes in module Blue from B. *infantis*.***

| **Locus Tag** | **Module** | **Annotation** |
| --- | --- | --- |
| Blon_0002 | blue | conserved hypothetical protein |
| Blon_0007 | blue | DNA gyrase, A subunit |
| Blon_0014 | blue | #N/D |
| Blon_0016 | blue | hypothetical protein |
| Blon_0022 | blue | phosphonate ABC transporter, ATPase subunit |
| Blon_0027 | blue | protein of unknown function DUF45 |
| Blon_0032 | blue | #N/D |
| Blon_0033 | blue | #N/D |
| Blon_0034 | blue | carbonic anhydrase |
| Blon_0036 | blue | FAD-dependent pyridine nucleotide-disulfide oxidoreductase |
| Blon_0038 | blue | conserved hypothetical protein |
| Blon_0040 | blue | transposase, mutator type |
| Blon_0041 | blue | conserved hypothetical protein |
| Blon_0053 | blue | extracellular solute-binding protein, family 5 |
| Blon_0054 | blue | binding-protein-dependent transport systems inner membrane component |
| Blon_0055 | blue | binding-protein-dependent transport systems inner membrane component |
| Blon_0064 | blue | tryptophanyl-tRNA synthetase |
| Blon_0065 | blue | conserved hypothetical protein |
| Blon_0066 | blue | two component transcriptional regulator, LuxR family |
| Blon_0069 | blue | narrowly conserved hypothetical protein |
| Blon_0075 | blue | sortase family protein |
| Blon_0076 | blue | glutamine amidotransferase of anthranilate synthase |
| Blon_0086 | blue | protein of unknown function DUF6, transmembrane |
| Blon_0101 | blue | queuine tRNA-ribosyltransferase |
| Blon_0104 | blue | Extracellular ligand-binding receptor |
| Blon_0105 | blue | inner-membrane translocator |
| Blon_0106 | blue | inner-membrane translocator |
| Blon_0108 | blue | ABC transporter related |
| Blon_0111 | blue | urease, alpha subunit |
| Blon_0112 | blue | UreE urease accessory domain protein |
| Blon_0113 | blue | Urease accessory protein UreF |
| Blon_0114 | blue | urease accessory protein UreG |
| Blon_0115 | blue | Urease accessory protein UreD |
| Blon_0121 | blue | hypothetical protein |
| Blon_0132 | blue | cobalt transport protein |
| Blon_0133 | blue | ABC transporter related |
| Blon_0134 | blue | BioY protein |
| Blon_0136 | blue | ketol-acid reductoisomerase |
| Blon_0150 | blue | DedA integral membrane protein |
| Blon_0151 | blue | conserved hypothetical protein |
| Blon_0157 | blue | conserved hypothetical protein |
| Blon_0160 | blue | DNA polymerase III, subunits gamma and tau |
| Blon_0161 | blue | recombination protein RecR |
| Blon_0162 | blue | sortase family protein |
| Blon_0169 | blue | conserved hypothetical protein |
| Blon_0171 | blue | glycosyl transferase, family 51 |
| Blon_0172 | blue | phosphoesterase, PA-phosphatase related |
| Blon_0174 | blue | dTMP kinase |
| Blon_0184 | blue | putative transcriptional regulator |
| Blon_0185 | blue | major facilitator superfamily MFS_1 |
| Blon_0191 | blue | glutamyl-tRNA synthetase |
| Blon_0193 | blue | protein of unknown function DUF1275 |
| Blon_0195 | blue | conserved hypothetical membrane protein with unknown function |
| Blon_0206 | blue | protein of unknown function DUF1113 |
| Blon_0218 | blue | peptidase U34, dipeptidase |
| Blon_0220 | blue | transcriptional regulator, TetR family |
| Blon_0223 | blue | ammonium transporter |
| Blon_0227 | blue | replicative DNA helicase |
| Blon_0229 | blue | CobB/CobQ domain protein glutamine amidotransferase |
| Blon_0233 | blue | ribose-phosphate pyrophosphokinase |
| Blon_0234 | blue | ribosomal protein S6 |
| Blon_0235 | blue | single-strand binding protein |
| Blon_0236 | blue | ribosomal protein S18 |
| Blon_0237 | blue | ribosomal protein L9 |
| Blon_0241 | blue | ABC-2 type transporter |
| Blon_0243 | blue | two component transcriptional regulator, LuxR family |
| Blon_0245 | blue | major facilitator superfamily MFS_1 |
| Blon_0252 | blue | protein of unknown function DUF156 |
| Blon_0253 | blue | protein of unknown function DUF195 |
| Blon_0263 | blue | histidine acid phosphatase |
| Blon_0270 | blue | transposase, mutator type |
| Blon_0271 | blue | phage integrase family protein |
| Blon_0272 | blue | #N/D |
| Blon_0292 | blue | helix-turn-helix domain protein |
| Blon_0304 | blue | ATP synthase F0, C subunit |
| Blon_0305 | blue | ATP synthase F0, B subunit |
| Blon_0306 | blue | ATP synthase F1, delta subunit |
| Blon_0307 | blue | ATP synthase F1, alpha subunit |
| Blon_0308 | blue | ATP synthase F1, gamma subunit |
| Blon_0311 | blue | protein of unknown function DUF91 |
| Blon_0321 | blue | extracellular solute-binding protein, family 1 |
| Blon_0324 | blue | ABC transporter related |
| Blon_0325 | blue | Abortive infection protein |
| Blon_0336 | blue | major facilitator superfamily MFS_1 |
| Blon_0345 | blue | major facilitator superfamily MFS_1 |
| Blon_0351 | blue | peptidase U32 |
| Blon_0352 | blue | protein of unknown function DUF152 |
| Blon_0356 | blue | Bleomycin hydrolase |
| Blon_0357 | blue | phospho-2-dehydro-3-deoxyheptonate aldolase |
| Blon_0358 | blue | phospho-2-dehydro-3-deoxyheptonate aldolase |
| Blon_0359 | blue | Methylthioadenosine nucleosidase |
| Blon_0362 | blue | two component transcriptional regulator, winged helix family |
| Blon_0372 | blue | aldo/keto reductase |
| Blon_0373 | blue | Inosine/uridine-preferring nucleoside hydrolase |
| Blon_0380 | blue | helix-turn-helix domain protein |
| Blon_0384 | blue | aminotransferase, class IV |
| Blon_0385 | blue | para-aminobenzoate synthase, subunit I |
| Blon_0395 | blue | ABC transporter related |
| Blon_0397 | blue | hypothetical protein |
| Blon_0398 | blue | acetolactate synthase, small subunit |
| Blon_0400 | blue | Ribonuclease III |
| Blon_0419 | blue | binding-protein-dependent transport systems inner membrane component |
| Blon_0420 | blue | ribosomal protein L19 |
| Blon_0426 | blue | Alpha-L-fucosidase |
| Blon_0432 | blue | hypothetical protein |
| Blon_0433 | blue | two component transcriptional regulator, LuxR family |
| Blon_0435 | blue | hypothetical protein |
| Blon_0441 | blue | conserved hypothetical protein |
| Blon_0445 | blue | drug resistance transporter, EmrB/QacA subfamily |
| Blon_0456 | blue | hypothetical protein |
| Blon_0464 | blue | histidine kinase, dimerisation and phosphoacceptor region |
| Blon_0506 | blue | hypothetical protein |
| Blon_0514 | blue | ABC transporter related |
| Blon_0529 | blue | hypothetical protein |
| Blon_0530 | blue | conserved hypothetical protein |
| Blon_0531 | blue | transcription factor WhiB |
| Blon_0532 | blue | hypothetical protein |
| Blon_0535 | blue | hypothetical protein |
| Blon_0554 | blue | acyl-CoA thioesterase |
| Blon_0557 | blue | #N/D |
| Blon_0558 | blue | major facilitator superfamily MFS_1 |
| Blon_0559 | blue | hypothetical protein |
| Blon_0565 | blue | putative glucokinase, ROK family |
| Blon_0577 | blue | Extracellular ligand-binding receptor |
| Blon_0578 | blue | inner-membrane translocator |
| Blon_0584 | blue | inosine-5'-monophosphate dehydrogenase |
| Blon_0587 | blue | #N/D |
| Blon_0589 | blue | prolyl-tRNA synthetase |
| Blon_0596 | blue | conserved hypothetical protein |
| Blon_0604 | blue | #N/D |
| Blon_0605 | blue | Endonuclease/exonuclease/phosphatase |
| Blon_0614 | blue | putative CoA-substrate-specific enzyme activase |
| Blon_0635 | blue | ABC transporter related |
| Blon_0650 | blue | ABC transporter related |
| Blon_0652 | blue | Aspartate transaminase |
| Blon_0656 | blue | ABC transporter related |
| Blon_0658 | blue | putative transposase |
| Blon_0676 | blue | conserved hypothetical protein |
| Blon_0684 | blue | conserved hypothetical protein |
| Blon_0688 | blue | conserved hypothetical protein |
| Blon_0703 | blue | metallophosphoesterase |
| Blon_0705 | blue | Histidine--tRNA ligase |
| Blon_0706 | blue | aspartyl-tRNA synthetase |
| Blon_0707 | blue | narrowly conserved hypothetical protein |
| Blon_0718 | blue | D-isomer specific 2-hydroxyacid dehydrogenase, NAD-binding |
| Blon_0722 | blue | ABC transporter related |
| Blon_0723 | blue | narrowly conserved hypothetical protein |
| Blon_0729 | blue | conserved hypothetical protein |
| Blon_0732 | blue | glycoside hydrolase, family 20 |
| Blon_0736 | blue | NusB antitermination factor |
| Blon_0748 | blue | Cystathionine gamma-synthase |
| Blon_0752 | blue | hypothetical protein |
| Blon_0753 | blue | Extracellular ligand-binding receptor |
| Blon_0760 | blue | extracellular solute-binding protein, family 3 |
| Blon_0767 | blue | Patatin |
| Blon_0768 | blue | glutamate racemase |
| Blon_0770 | blue | Vitamin K epoxide reductase |
| Blon_0776 | blue | peptidase S16, lon domain protein |
| Blon_0787 | blue | Glycosyl hydrolase family 32, N terminal domain protein |
| Blon_0789 | blue | periplasmic binding protein/LacI transcriptional regulator |
| Blon_0791 | blue | hypothetical protein |
| Blon_0795 | blue | Vesicle-fusing ATPase |
| Blon_0799 | blue | putative TIM-barrel protein, nifR3 family |
| Blon_0808 | blue | DNA polymerase III, alpha subunit |
| Blon_0816 | blue | Transposase and inactivated derivatives-like protein |
| Blon_0821 | blue | carbohydrate kinase, FGGY |
| Blon_0831 | blue | major facilitator superfamily MFS_1 |
| Blon_0836 | blue | conserved hypothetical protein |
| Blon_0837 | blue | ATP-dependent helicase HrpA |
| Blon_0838 | blue | methyltransferase small |
| Blon_0841 | blue | cation diffusion facilitator family transporter |
| Blon_0843 | blue | Peptidoglycan-binding LysM |
| Blon_0851 | blue | conserved hypothetical protein |
| Blon_0861 | blue | #N/D |
| Blon_0870 | blue | extracellular solute-binding protein, family 5 |
| Blon_0871 | blue | binding-protein-dependent transport systems inner membrane component |
| Blon_0879 | blue | ROK family protein |
| Blon_0881 | blue | glucosamine-6-phosphate isomerase |
| Blon_0903 | blue | ribosomal protein L35 |
| Blon_0905 | blue | tyrosine recombinase XerD |
| Blon_0910 | blue | quinolinate synthetase complex, A subunit |
| Blon_0911 | blue | L-aspartate oxidase |
| Blon_0913 | blue | aminotransferase, class V |
| Blon_0914 | blue | GTP-binding protein TypA |
| Blon_0928 | blue | narrowly conserved hypothetical transmembrane protein |
| Blon_0933 | blue | RelB antitoxin |
| Blon_0941 | blue | RNA modification enzyme, MiaB family |
| Blon_0944 | blue | cell divisionFtsK/SpoIIIE |
| Blon_0950 | blue | regulatory protein RecX |
| Blon_0958 | blue | Polyprenyl synthetase |
| Blon_0965 | blue | two component transcriptional regulator, winged helix family |
| Blon_0966 | blue | DNA gyrase/topoisomerase IV, subunit A |
| Blon_0967 | blue | type I phosphodiesterase/nucleotide pyrophosphatase |
| Blon_0969 | blue | narrowly conserved hypothetical protein |
| Blon_0971 | blue | (p)ppGpp synthetase I, SpoT/RelA |
| Blon_0976 | blue | #N/D |
| Blon_0981 | blue | hypothetical protein |
| Blon_0993 | blue | hypothetical protein |
| Blon_0994 | blue | transcriptional regulator, Fis family |
| Blon_1003 | blue | two component transcriptional regulator, winged helix family |
| Blon_1005 | blue | conserved hypothetical protein |
| Blon_1010 | blue | Mg2+ transporter protein, CorA family protein |
| Blon_1011 | blue | extracellular solute-binding protein, family 3 |
| Blon_1012 | blue | leucyl-tRNA synthetase |
| Blon_1013 | blue | competence protein ComEA helix-hairpin-helix repeat protein |
| Blon_1014 | blue | ComEC/Rec2-related protein |
| Blon_1016 | blue | protein of unknown function UPF0079 |
| Blon_1020 | blue | hypothetical protein |
| Blon_1041 | blue | #N/D |
| Blon_1043 | blue | hypothetical protein |
| Blon_1045 | blue | hypothetical protein |
| Blon_1049 | blue | peptidase M23B |
| Blon_1050 | blue | conserved hypothetical protein |
| Blon_1052 | blue | IMP dehydrogenase family protein |
| Blon_1056 | blue | ribosomal protein S2 |
| Blon_1062 | blue | ThiJ/PfpI domain protein |
| Blon_1065 | blue | anthranilate synthase component I |
| Blon_1067 | blue | protein of unknown function DUF979 |
| Blon_1068 | blue | protein of unknown function DUF969 |
| Blon_1069 | blue | ABC transporter related |
| Blon_1070 | blue | short-chain dehydrogenase/reductase SDR |
| Blon_1071 | blue | conserved hypothetical protein |
| Blon_1074 | blue | #N/D |
| Blon_1075 | blue | #N/D |
| Blon_1079 | blue | conserved hypothetical protein |
| Blon_1085 | blue | Uncharacterised P-loop ATPase protein UPF0042 |
| Blon_1086 | blue | protein of unknown function DUF199 |
| Blon_1094 | blue | branched-chain amino acid transport system II carrier protein |
| Blon_1104 | blue | hypothetical protein |
| Blon_1159 | blue | preprotein translocase YajC subunit |
| Blon_1162 | blue | succinyl-CoA synthetase, alpha subunit |
| Blon_1166 | blue | pseudouridine synthase |
| Blon_1167 | blue | small GTP-binding protein |
| Blon_1185 | blue | leucine rich repeat variant |
| Blon_1194 | blue | hypothetical protein |
| Blon_1200 | blue | N-formylglutamate amidohydrolase |
| Blon_1207 | blue | hypothetical protein |
| Blon_1208 | blue | hypothetical protein |
| Blon_1211 | blue | hypothetical protein |
| Blon_1227 | blue | LPXTG-motif cell wall anchor domain protein |
| Blon_1228 | blue | #N/D |
| Blon_1232 | blue | conserved hypothetical protein |
| Blon_1236 | blue | hypothetical protein |
| Blon_1237 | blue | Exonuclease, RNase T and DNA polymerase III |
| Blon_1248 | blue | hypothetical protein |
| Blon_1249 | blue | DNA polymerase III, beta subunit |
| Blon_1250 | blue | hypothetical protein |
| Blon_1255 | blue | hypothetical protein |
| Blon_1271 | blue | hypothetical protein |
| Blon_1273 | blue | hypothetical protein |
| Blon_1274 | blue | hypothetical protein |
| Blon_1275 | blue | hypothetical protein |
| Blon_1277 | blue | hypothetical protein |
| Blon_1278 | blue | hypothetical protein |
| Blon_1294 | blue | hypothetical protein |
| Blon_1296 | blue | hypothetical protein |
| Blon_1306 | blue | hypothetical protein |
| Blon_1314 | blue | hypothetical protein |
| Blon_1316 | blue | hypothetical protein |
| Blon_1319 | blue | hypothetical protein |
| Blon_1322 | blue | hypothetical protein |
| Blon_1346 | blue | DNA methylase N-4/N-6 domain protein |
| Blon_1356 | blue | Integrase, catalytic region |
| Blon_1369 | blue | prolipoprotein diacylglyceryl transferase |
| Blon_1370 | blue | tryptophan synthase, alpha subunit |
| Blon_1371 | blue | tryptophan synthase, beta subunit |
| Blon_1374 | blue | amino acid permease-associated region |
| Blon_1375 | blue | conserved hypothetical membrane protein with unknown function |
| Blon_1376 | blue | VanZ family protein |
| Blon_1391 | blue | conserved hypothetical protein |
| Blon_1392 | blue | Signal transduction histidine kinase-like protein |
| Blon_1394 | blue | hypothetical protein |
| Blon_1396 | blue | ABC transporter related |
| Blon_1397 | blue | protein of unknown function DUF214 |
| Blon_1407 | blue | hypothetical protein |
| Blon_1408 | blue | protein of unknown function DUF6, transmembrane |
| Blon_1427 | blue | amino acid permease-associated region |
| Blon_1431 | blue | hypothetical protein |
| Blon_1433 | blue | hypothetical protein |
| Blon_1434 | blue | #N/D |
| Blon_1435 | blue | Death-on-curing protein |
| Blon_1459 | blue | hypothetical protein |
| Blon_1460 | blue | transcriptional activator, TenA family |
| Blon_1462 | blue | #N/D |
| Blon_1481 | blue | glutamate synthase, NADH/NADPH, small subunit |
| Blon_1482 | blue | Glutamate synthase (ferredoxin) |
| Blon_1497 | blue | hypothetical protein |
| Blon_1500 | blue | hypothetical protein |
| Blon_1502 | blue | hypothetical protein |
| Blon_1505 | blue | single-strand binding protein |
| Blon_1506 | blue | hypothetical protein |
| Blon_1507 | blue | hypothetical protein |
| Blon_1508 | blue | hypothetical protein |
| Blon_1510 | blue | hypothetical protein |
| Blon_1511 | blue | hypothetical protein |
| Blon_1512 | blue | hypothetical protein |
| Blon_1513 | blue | hypothetical protein |
| Blon_1515 | blue | hypothetical protein |
| Blon_1520 | blue | hypothetical protein |
| Blon_1521 | blue | hypothetical protein |
| Blon_1522 | blue | hypothetical protein |
| Blon_1523 | blue | hypothetical protein |
| Blon_1532 | blue | hypothetical protein |
| Blon_1560 | blue | hypothetical protein |
| Blon_1565 | blue | hypothetical protein |
| Blon_1571 | blue | glycoside hydrolase, family 25 |
| Blon_1572 | blue | hypothetical protein |
| Blon_1573 | blue | hypothetical protein |
| Blon_1587 | blue | tRNA/rRNA methyltransferase (SpoU) |
| Blon_1590 | blue | SUF system FeS assembly protein, NifU family |
| Blon_1597 | blue | Shikimate kinase., 3-dehydroquinate synthase |
| Blon_1602 | blue | alanyl-tRNA synthetase |
| Blon_1612 | blue | RNA-binding S4 domain protein |
| Blon_1616 | blue | UvrD/REP helicase |
| Blon_1636 | blue | alpha amylase, catalytic region |
| Blon_1644 | blue | major facilitator superfamily MFS_1 |
| Blon_1650 | blue | drug resistance transporter, EmrB/QacA subfamily |
| Blon_1653 | blue | hypothetical protein |
| Blon_1672 | blue | IstB domain protein ATP-binding protein |
| Blon_1673 | blue | prophage LambdaBa04, site-specific recombinase, phage integrase family |
| Blon_1681 | blue | Methyltransferase type 11 |
| Blon_1699 | blue | Mg chelatase, subunit ChlI |
| Blon_1700 | blue | SMF family protein |
| Blon_1710 | blue | Cl- channel, voltage-gated family protein |
| Blon_1711 | blue | trigger factor |
| Blon_1714 | blue | pyruvate formate-lyase activating enzyme |
| Blon_1724 | blue | #N/D |
| Blon_1727 | blue | Nucleotidyl transferase |
| Blon_1734 | blue | hypothetical protein |
| Blon_1749 | blue | two component transcriptional regulator, LuxR family |
| Blon_1751 | blue | conserved hypothetical protein |
| Blon_1770 | blue | transcription factor WhiB |
| Blon_1772 | blue | cell divisionFtsK/SpoIIIE |
| Blon_1802 | blue | hypothetical protein |
| Blon_1805 | blue | hypothetical protein |
| Blon_1821 | blue | prophage LambdaSa03, structural protein, putative |
| Blon_1823 | blue | phage tape measure protein |
| Blon_1825 | blue | Fibronectin, type III domain protein |
| Blon_1827 | blue | hypothetical protein |
| Blon_1833 | blue | Ppx/GppA phosphatase |
| Blon_1838 | blue | transcription-repair coupling factor |
| Blon_1839 | blue | Aminoacyl-tRNA hydrolase |
| Blon_1851 | blue | #N/D |
| Blon_1856 | blue | two component transcriptional regulator, LuxR family |
| Blon_1860 | blue | HAD-superfamily hydrolase, subfamily IA, variant 3 |
| Blon_1863 | blue | conserved hypothetical protein |
| Blon_1864 | blue | hemolysin A |
| Blon_1865 | blue | HAD-superfamily hydrolase, subfamily IIA |
| Blon_1866 | blue | conserved hypothetical protein |
| Blon_1868 | blue | conserved hypothetical protein |
| Blon_1869 | blue | metal dependent phosphohydrolase |
| Blon_1874 | blue | argininosuccinate lyase |
| Blon_1875 | blue | Argininosuccinate synthase |
| Blon_1881 | blue | N-acetyl-gamma-glutamyl-phosphate reductase |
| Blon_1882 | blue | conserved hypothetical protein |
| Blon_1883 | blue | phenylalanyl-tRNA synthetase, beta subunit |
| Blon_1884 | blue | phenylalanyl-tRNA synthetase, alpha subunit |
| Blon_1886 | blue | cobalt transport protein |
| Blon_1888 | blue | narrowly conserved hypothetical protein |
| Blon_1889 | blue | peptidase M20 |
| Blon_1891 | blue | dihydrolipoamide dehydrogenase |
| Blon_1893 | blue | glutamine synthetase, type I |
| Blon_1894 | blue | #N/D |
| Blon_1897 | blue | galactoside O-acetyltransferase |
| Blon_1911 | blue | protein of unknown function DUF214 |
| Blon_1917 | blue | Camphor resistance CrcB protein |
| Blon_1920 | blue | conserved hypothetical protein 698 |
| Blon_1923 | blue | translation elongation factor G |
| Blon_1924 | blue | ribosomal protein S7 |
| Blon_1925 | blue | ribosomal protein S12 |
| Blon_1937 | blue | aminotransferase, class I and II |
| Blon_1948 | blue | #N/D |
| Blon_1949 | blue | #N/D |
| Blon_1950 | blue | hypothetical protein |
| Blon_1951 | blue | UMUC domain protein DNA-repair protein |
| Blon_1960 | blue | phosphoribosylamine--glycine ligase |
| Blon_1971 | blue | putative high-affinity zinc ABC transporter |
| Blon_1976 | blue | hypothetical protein |
| Blon_1977 | blue | #N/D |
| Blon_1979 | blue | ferric uptake regulator, Fur family |
| Blon_1980 | blue | phosphoribosylaminoimidazole carboxylase, ATPase subunit |
| Blon_1981 | blue | phosphoribosylaminoimidazole carboxylase, catalytic subunit |
| Blon_1983 | blue | 1-deoxy-D-xylulose-5-phosphate synthase |
| Blon_1988 | blue | transcriptional regulator, RpiR family |
| Blon_1997 | blue | alanine racemase |
| Blon_2002 | blue | ErfK/YbiS/YcfS/YnhG family protein |
| Blon_2003 | blue | Cystathionine gamma-synthase |
| Blon_2009 | blue | ABC transporter related |
| Blon_2010 | blue | binding-protein-dependent transport systems inner membrane component |
| Blon_2013 | blue | phosphopantothenoylcysteine decarboxylase/phosphopantothenate--cysteine ligase |
| Blon_2014 | blue | putative transcriptional activator, Baf family |
| Blon_2016 | blue | Beta-galactosidase |
| Blon_2021 | blue | extracellular solute-binding protein, family 3 |
| Blon_2023 | blue | SsrA-binding protein |
| Blon_2024 | blue | CHAP domain-containing protein |
| Blon_2025 | blue | protein of unknown function DUF214 |
| Blon_2026 | blue | cell division ATP-binding protein FtsE |
| Blon_2027 | blue | peptide chain release factor 2 |
| Blon_2037 | blue | UvrD/REP helicase |
| Blon_2039 | blue | protein kinase |
| Blon_2048 | blue | DNA-directed RNA polymerase, beta' subunit |
| Blon_2049 | blue | DNA-directed RNA polymerase, beta subunit |
| Blon_2052 | blue | tRNA/rRNA methyltransferase (SpoU) |
| Blon_2056 | blue | Glycosyl hydrolase family 32, N terminal domain protein |
| Blon_2067 | blue | glycosyl transferase, family 51 |
| Blon_2079 | blue | transcriptional regulator, XRE family |
| Blon_2083 | blue | conserved hypothetical protein |
| Blon_2086 | blue | transcriptional regulator, Fis family |
| Blon_2106 | blue | glycosyl transferase, family 2 |
| Blon_2109 | blue | glycosyl transferase, family 2 |
| Blon_2114 | blue | Undecaprenyl-phosphate galactose phosphotransferase |
| Blon_2116 | blue | conserved hypothetical protein |
| Blon_2130 | blue | phosphoserine phosphatase SerB |
| Blon_2134 | blue | dihydroxy-acid dehydratase |
| Blon_2137 | blue | protein of unknown function DUF214 |
| Blon_2159 | blue | major facilitator superfamily MFS_1 |
| Blon_2161 | blue | lysyl-tRNA synthetase |
| Blon_2162 | blue | major facilitator superfamily MFS_1 |
| Blon_2163 | blue | LPXTG-motif cell wall anchor domain protein |
| Blon_2190 | blue | ribonuclease H |
| Blon_2197 | blue | ribosome-binding factor A |
| Blon_2198 | blue | translation initiation factor IF-2 |
| Blon_2207 | blue | transglutaminase, N-terminal domain protein |
| Blon_2212 | blue | ribosomal protein S11 |
| Blon_2215 | blue | translation initiation factor IF-1 |
| Blon_2221 | blue | ribosomal protein L18 |
| Blon_2222 | blue | ribosomal protein L6 |
| Blon_2223 | blue | ribosomal protein S8 |
| Blon_2224 | blue | ribosomal protein S14 |
| Blon_2225 | blue | ribosomal protein L5 |
| Blon_2226 | blue | ribosomal protein L24 |
| Blon_2227 | blue | ribosomal protein L14 |
| Blon_2228 | blue | ribosomal protein S17 |
| Blon_2229 | blue | ribosomal protein L29 |
| Blon_2230 | blue | ribosomal protein L16 |
| Blon_2231 | blue | ribosomal protein S3 |
| Blon_2232 | blue | ribosomal protein L22 |
| Blon_2233 | blue | ribosomal protein S19 |
| Blon_2234 | blue | ribosomal protein L2 |
| Blon_2235 | blue | Ribosomal protein L25/L23 |
| Blon_2236 | blue | ribosomal protein L4/L1e |
| Blon_2237 | blue | ribosomal protein L3 |
| Blon_2238 | blue | ribosomal protein S10 |
| Blon_2239 | blue | Acetolactate decarboxylase |
| Blon_2244 | blue | ribosomal protein S9 |
| Blon_2245 | blue | ribosomal protein L13 |
| Blon_2248 | blue | protein of unknown function UPF0029 |
| Blon_2249 | blue | D-isomer-specific 2-hydroxy acid dehydrogenase, NAD-binding |
| Blon_2251 | blue | aminotransferase, class I and II |
| Blon_2252 | blue | 4Fe-4S ferredoxin, iron-sulfur binding domain protein |
| Blon_2269 | blue | Xanthine/uracil/vitamin C permease |
| Blon_2270 | blue | Polyribonucleotide nucleotidyltransferase |
| Blon_2278 | blue | hypothetical protein |
| Blon_2279 | blue | Integrase, catalytic region |
| Blon_2280 | blue | conserved hypothetical protein |
| Blon_2282 | blue | hypothetical protein |
| Blon_2283 | blue | 4'-phosphopantetheinyl transferase |
| Blon_2284 | blue | Unknown function domain DUF1729 |
| Blon_2286 | blue | Carbamoyl-phosphate synthase L chain, ATP-binding |
| Blon_2287 | blue | hypothetical protein |
| Blon_2290 | blue | narrowly conserved hypothetical protein |
| Blon_2292 | blue | regulatory protein, IclR |
| Blon_2294 | blue | ribosomal protein L11 |
| Blon_2296 | blue | preprotein translocase, SecE subunit |
| Blon_2300 | blue | ribosomal protein L27 |
| Blon_2301 | blue | ribosomal protein L21 |
| Blon_2302 | blue | ribonuclease, Rne/Rng family |
| Blon_2311 | blue | protein of unknown function DUF214 |
| Blon_2312 | blue | ABC transporter related |
| Blon_2313 | blue | hypothetical protein |
| Blon_2319 | blue | transcriptional regulator, LysR family |
| Blon_2323 | blue | dihydroorotate dehydrogenase |
| Blon_2332 | blue | sugar (Glycoside-Pentoside-Hexuronide) transporter |
| Blon_2335 | blue | conserved hypothetical protein |
| Blon_2336 | blue | alpha-1,3/4-fucosidase, putative |
| Blon_2337 | blue | RbsD or FucU transport |
| Blon_2338 | blue | dihydrodipicolinate synthetase |
| Blon_2339 | blue | short-chain dehydrogenase/reductase SDR |
| Blon_2340 | blue | Mandelate racemase/muconate lactonizing enzyme, C-terminal domain protein |
| Blon_2341 | blue | protein of unknown function DUF624 |
| Blon_2342 | blue | binding-protein-dependent transport systems inner membrane component |
| Blon_2344 | blue | extracellular solute-binding protein, family 1 |
| Blon_2345 | blue | binding-protein-dependent transport systems inner membrane component |
| Blon_2347 | blue | extracellular solute-binding protein, family 1 |
| Blon_2348 | blue | Exo-alpha-sialidase |
| Blon_2349 | blue | dihydrodipicolinate synthetase |
| Blon_2356 | blue | Haloacid dehalogenase domain protein hydrolase |
| Blon_2358 | blue | beta-lactamase domain protein |
| Blon_2373 | blue | GCN5-related N-acetyltransferase |
| Blon_2383 | blue | hypothetical protein |
| Blon_2388 | blue | glycosyl transferase, family 8 |
| Blon_2391 | blue | acyltransferase 3 |
| Blon_2393 | blue | protein of unknown function DUF214 |
| Blon_2404 | blue | transcriptional regulator, Fis family |
| Blon_2419 | blue | extracellular solute-binding protein, family 5 |
| Blon_2426 | blue | methionyl-tRNA synthetase |
| Blon_2428 | blue | conserved hypothetical protein |
| Blon_2429 | blue | conserved hypothetical protein |
| Blon_2449 | blue | ABC-2 type transporter |
| Blon_2454 | blue | hypothetical protein |
| Blon_2459 | blue | ROK family protein |
| Blon_2462 | blue | sodium/hydrogen exchanger |
| Blon_2464 | blue | conserved hypothetical protein |
| Blon_2476 | blue | glycosyl transferase, family 2 |
| Blon_2477 | blue | Ribonucleoside-diphosphate reductase |
| Blon_2478 | blue | ribonucleoside-diphosphate reductase, alpha subunit |
| Blon_2481 | blue | protein kinase |
| Blon_2482 | blue | conserved hypothetical protein |
| Blon_2491 | blue | thioredoxin reductase |
| Blon_2498 | blue | ribonuclease P protein component |
| Blon_R0013 | blue | #N/D |
| Blon_R0015 | blue | #N/D |
| Blon_R0020 | blue | #N/D |
| Blon_R0040 | blue | #N/D |
| Blon_R0082 | blue | #N/D |
| Blon_R0083 | blue | #N/D |

*** Shaded rows refer to genes associated with HMO consumption, according to Garrido et al., (2015).**

**Table S4. Genes in module Darkturquoise for B. *bifidum* SC555**.***

| **Locus Tag** | **Module** | **Annotation** |
| --- | --- | --- |
| BBIF_00001 | darkturquoise | hypothetical protein |
| BBIF_00002 | darkturquoise | O-acetylhomoserine (thiol)-lyase |
| BBIF_00004 | darkturquoise | aldehyde dehydrogenase (NAD+) |
| BBIF_00008 | darkturquoise | alpha-L-fucosidase |
| BBIF_00009 | darkturquoise | D-isomer specific 2-hydroxyacid dehydrogenase |
| BBIF_00010 | darkturquoise | transcriptional regulator |
| BBIF_00027 | darkturquoise | NAD(P) transhydrogenase subunit alpha |
| BBIF_00028 | darkturquoise | NAD(P) transhydrogenase subunit alpha |
| BBIF_00029 | darkturquoise | NAD(P) transhydrogenase subunit beta |
| BBIF_00031 | darkturquoise | large subunit ribosomal protein L25 |
| BBIF_00035 | darkturquoise | SSU ribosomal protein S20P |
| BBIF_00042 | darkturquoise | hypothetical protein |
| BBIF_00059 | darkturquoise | Acetyl esterase/lipase |
| BBIF_00062 | darkturquoise | methionine synthase (B12-independent) |
| BBIF_00063 | darkturquoise | 5,10-methylenetetrahydrofolate reductase (NAD(P)) |
| BBIF_00064 | darkturquoise | hypothetical protein |
| BBIF_00082 | darkturquoise | Transcriptional regulator PadR-like family protein |
| BBIF_00118 | darkturquoise | Prolipoprotein diacylglyceryl transferase |
| BBIF_00126 | darkturquoise | hypothetical protein |
| BBIF_00135 | darkturquoise | DNA-binding regulatory protein |
| BBIF_00136 | darkturquoise | hexosaminidase |
| BBIF_00137 | darkturquoise | threonyl-tRNA synthetase |
| BBIF_00139 | darkturquoise | #N/D |
| BBIF_00157 | darkturquoise | malate dehydrogenase (NAD) |
| BBIF_00158 | darkturquoise | preprotein translocase subunit SecG |
| BBIF_00162 | darkturquoise | UPF0042 nucleotide-binding protein |
| BBIF_00163 | darkturquoise | shikimate dehydrogenase |
| BBIF_00175 | darkturquoise | Sugar kinase of the NBD/HSP70 family |
| BBIF_00176 | darkturquoise | Sugar kinase of the NBD/HSP70 family |
| BBIF_00177 | darkturquoise | 1,3-beta-galactosyl-N-acetylhexosamine phosphorylase |
| BBIF_00226 | darkturquoise | Predicted lactoylglutathione lyase |
| BBIF_00236 | darkturquoise | hypothetical protein |
| BBIF_00246 | darkturquoise | #N/D |
| BBIF_00261 | darkturquoise | prepilin-type processing-associated H-X9-DG domain-containing protein |
| BBIF_00262 | darkturquoise | 23S rRNA (cytidine1920-2'-O)/16S rRNA (cytidine1409-2'-O)-methyltransferase |
| BBIF_00263 | darkturquoise | hypothetical protein |
| BBIF_00264 | darkturquoise | Haloacid Dehalogenase Superfamily Class (subfamily) IIA |
| BBIF_00267 | darkturquoise | hypothetical protein |
| BBIF_00269 | darkturquoise | Uncharacterized membrane protein |
| BBIF_00270 | darkturquoise | uncharacterized protein |
| BBIF_00284 | darkturquoise | hypothetical protein |
| BBIF_00285 | darkturquoise | inorganic phosphate transporter |
| BBIF_00299 | darkturquoise | DNA-binding transcriptional regulator |
| BBIF_00308 | darkturquoise | NUDIX domain-containing protein |
| BBIF_00309 | darkturquoise | dinuclear metal center protein |
| BBIF_00310 | darkturquoise | DNA polymerase I |
| BBIF_00312 | darkturquoise | #N/D |
| BBIF_00317 | darkturquoise | dephospho-CoA kinase |
| BBIF_00323 | darkturquoise | 2-C-methyl-D-erythritol 2 |
| BBIF_00324 | darkturquoise | transcriptional regulator |
| BBIF_00325 | darkturquoise | 1,4-alpha-glucan branching enzyme |
| BBIF_00328 | darkturquoise | DNA polymerase V |
| BBIF_00340 | darkturquoise | hypothetical protein |
| BBIF_00346 | darkturquoise | #N/D |
| BBIF_00362 | darkturquoise | tRNA (cytidine/uridine-2'-O-)-methyltransferase |
| BBIF_00366 | darkturquoise | ACT domain-containing protein |
| BBIF_00369 | darkturquoise | UDPglucose--hexose-1-phosphate uridylyltransferase |
| BBIF_00370 | darkturquoise | transcriptional regulator |
| BBIF_00375 | darkturquoise | lipoate-protein ligase A |
| BBIF_00377 | darkturquoise | oligopeptidase B |
| BBIF_00396 | darkturquoise | pyridoxal phosphate synthase yaaD subunit |
| BBIF_00398 | darkturquoise | transcriptional regulator |
| BBIF_00402 | darkturquoise | L-ribulose 5-phosphate 4-epimerase |
| BBIF_00407 | darkturquoise | #N/D |
| BBIF_00408 | darkturquoise | NitT/TauT family transport system permease protein |
| BBIF_00409 | darkturquoise | NitT/TauT family transport system ATP-binding protein |
| BBIF_00418 | darkturquoise | #N/D |
| BBIF_00420 | darkturquoise | hypothetical protein |
| BBIF_00421 | darkturquoise | XTP/dITP diphosphohydrolase |
| BBIF_00422 | darkturquoise | RNAse PH |
| BBIF_00428 | darkturquoise | large subunit ribosomal protein L32 |
| BBIF_00432 | darkturquoise | Protein of unknown function (DUF3039) |
| BBIF_00433 | darkturquoise | ABC transporter |
| BBIF_00442 | darkturquoise | small subunit ribosomal protein S16 |
| BBIF_00444 | darkturquoise | 16S rRNA processing protein RimM |
| BBIF_00455 | darkturquoise | LSU ribosomal protein L28P |
| BBIF_00456 | darkturquoise | Sugar or nucleoside kinase |
| BBIF_00457 | darkturquoise | hypothetical protein |
| BBIF_00466 | darkturquoise | #N/D |
| BBIF_00468 | darkturquoise | #N/D |
| BBIF_00471 | darkturquoise | long-chain acyl-CoA synthetase |
| BBIF_00472 | darkturquoise | hypothetical protein |
| BBIF_00488 | darkturquoise | glutamyl-tRNA synthetase |
| BBIF_00497 | darkturquoise | small subunit ribosomal protein S6 |
| BBIF_00498 | darkturquoise | Double zinc ribbon |
| BBIF_00500 | darkturquoise | hypothetical protein |
| BBIF_00509 | darkturquoise | preprotein translocase subunit SecE |
| BBIF_00510 | darkturquoise | #N/D |
| BBIF_00511 | darkturquoise | Aspartate/methionine/tyrosine aminotransferase |
| BBIF_00518 | darkturquoise | succinyldiaminopimelate desuccinylase |
| BBIF_00519 | darkturquoise | hypothetical protein |
| BBIF_00537 | darkturquoise | hypothetical protein |
| BBIF_00544 | darkturquoise | LacI family transcriptional regulator |
| BBIF_00545 | darkturquoise | N-acetylglucosamine-6-phosphate deacetylase |
| BBIF_00555 | darkturquoise | lactaldehyde reductase |
| BBIF_00556 | darkturquoise | Glycosyl transferase family 2 |
| BBIF_00570 | darkturquoise | hexosaminidase |
| BBIF_00591 | darkturquoise | hypothetical protein |
| BBIF_00595 | darkturquoise | Superfamily II DNA and RNA helicase |
| BBIF_00596 | darkturquoise | beta-lactamase class A |
| BBIF_00597 | darkturquoise | glucose-6-phosphate 1-epimerase |
| BBIF_00602 | darkturquoise | AraC family transcriptional regulator |
| BBIF_00604 | darkturquoise | LSU ribosomal protein L31P |
| BBIF_00606 | darkturquoise | release factor glutamine methyltransferase |
| BBIF_00607 | darkturquoise | maltose O-acetyltransferase |
| BBIF_00624 | darkturquoise | DNA polymerase-3 subunit epsilon |
| BBIF_00630 | darkturquoise | glycerate kinase |
| BBIF_00631 | darkturquoise | NADPH-dependent 2 |
| BBIF_00632 | darkturquoise | cystathionine beta-lyase |
| BBIF_00633 | darkturquoise | PTS system |
| BBIF_00634 | darkturquoise | PTS system |
| BBIF_00642 | darkturquoise | hypothetical protein |
| BBIF_00643 | darkturquoise | high-affinity iron transporter |
| BBIF_00650 | darkturquoise | transcriptional regulator |
| BBIF_00660 | darkturquoise | 2-isopropylmalate synthase |
| BBIF_00663 | darkturquoise | PASTA domain-containing protein |
| BBIF_00664 | darkturquoise | hypothetical protein |
| BBIF_00666 | darkturquoise | aspartate kinase |
| BBIF_00667 | darkturquoise | aspartate kinase |
| BBIF_00672 | darkturquoise | Protein of unknown function (DUF4244) |
| BBIF_00679 | darkturquoise | 8-oxo-dGTP diphosphatase |
| BBIF_00690 | darkturquoise | ABC-type antimicrobial peptide transport system |
| BBIF_00691 | darkturquoise | ABC-type lipoprotein export system |
| BBIF_00692 | darkturquoise | uncharacterized protein |
| BBIF_00693 | darkturquoise | Glutamine amidotransferase domain-containing protein |
| BBIF_00704 | darkturquoise | #N/D |
| BBIF_00707 | darkturquoise | sialidase-1 |
| BBIF_00712 | darkturquoise | alkaline phosphatase |
| BBIF_00714 | darkturquoise | hypothetical protein |
| BBIF_00736 | darkturquoise | Transposon-encoded protein TnpV |
| BBIF_00741 | darkturquoise | Protein of unknown function (DUF2442) |
| BBIF_00742 | darkturquoise | hypothetical protein |
| BBIF_00747 | darkturquoise | holo-[acyl-carrier protein] synthase |
| BBIF_00748 | darkturquoise | BirA family transcriptional regulator |
| BBIF_00752 | darkturquoise | SSU ribosomal protein S15P |
| BBIF_00754 | darkturquoise | regulatory protein |
| BBIF_00757 | darkturquoise | methionine-gamma-lyase |
| BBIF_00758 | darkturquoise | ribonucleoside-diphosphate reductase class Ib beta subunit |
| BBIF_00759 | darkturquoise | ribonucleoside-diphosphate reductase alpha chain |
| BBIF_00760 | darkturquoise | protein involved in ribonucleotide reduction |
| BBIF_00761 | darkturquoise | glutaredoxin-like protein NrdH |
| BBIF_00764 | darkturquoise | putative protease |
| BBIF_00771 | darkturquoise | 3-deoxy-D-arabinoheptulosonate-7-phosphate synthase |
| BBIF_00777 | darkturquoise | two-component system |
| BBIF_00783 | darkturquoise | hypothetical protein |
| BBIF_00785 | darkturquoise | hypothetical protein |
| BBIF_00786 | darkturquoise | Ig-like domain (group 2) |
| BBIF_00787 | darkturquoise | Lysophospholipase L1 |
| BBIF_00803 | darkturquoise | #N/D |
| BBIF_00804 | darkturquoise | #N/D |
| BBIF_00805 | darkturquoise | LSU ribosomal protein L33P |
| BBIF_00810 | darkturquoise | #N/D |
| BBIF_00811 | darkturquoise | poly-gamma-glutamate synthesis protein (capsule biosynthesis protein) |
| BBIF_00812 | darkturquoise | uncharacterized protein |
| BBIF_00813 | darkturquoise | Leucine rich repeat variant |
| BBIF_00814 | darkturquoise | 4-alpha-glucanotransferase |
| BBIF_00815 | darkturquoise | LSU ribosomal protein L13P |
| BBIF_00816 | darkturquoise | SSU ribosomal protein S9P |
| BBIF_00831 | darkturquoise | LSU ribosomal protein L16P |
| BBIF_00833 | darkturquoise | SSU ribosomal protein S17P |
| BBIF_00843 | darkturquoise | LSU ribosomal protein L15P |
| BBIF_00852 | darkturquoise | hexosaminidase |
| BBIF_00872 | darkturquoise | hypothetical protein |
| BBIF_00881 | darkturquoise | F5/8 type C domain-containing protein |
| BBIF_00882 | darkturquoise | hypothetical protein |
| BBIF_00893 | darkturquoise | Protein of unknown function (DUF4125) |
| BBIF_00894 | darkturquoise | Tetratricopeptide (TPR) repeat |
| BBIF_00900 | darkturquoise | hypothetical protein |
| BBIF_00905 | darkturquoise | CHAP domain-containing protein |
| BBIF_00907 | darkturquoise | Nucleotide-binding universal stress protein |
| BBIF_00913 | darkturquoise | Branched-chain amino acid transport protein (AzlD) |
| BBIF_00931 | darkturquoise | hypothetical protein |
| BBIF_00933 | darkturquoise | Protein of unknown function (DUF3043) |
| BBIF_00934 | darkturquoise | dihydrolipoamide dehydrogenase |
| BBIF_00935 | darkturquoise | protein of unknown function (DUF4191) |
| BBIF_00942 | darkturquoise | hypothetical protein |
| BBIF_00945 | darkturquoise | Methyltransferase domain-containing protein |
| BBIF_00947 | darkturquoise | hypothetical protein |
| BBIF_00948 | darkturquoise | ATP-binding protein involved in chromosome partitioning |
| BBIF_00980 | darkturquoise | cold shock protein (beta-ribbon |
| BBIF_00981 | darkturquoise | two-component system |
| BBIF_00988 | darkturquoise | Uracil-DNA glycosylase |
| BBIF_01011 | darkturquoise | DNA-directed RNA polymerase subunit omega |
| BBIF_01016 | darkturquoise | hypothetical protein |
| BBIF_01017 | darkturquoise | tRNA/tmRNA/rRNA uracil-C5-methylase |
| BBIF_01019 | darkturquoise | Protein of unknown function (DUF3710) |
| BBIF_01041 | darkturquoise | LSU ribosomal protein L20P |
| BBIF_01042 | darkturquoise | large subunit ribosomal protein L35 |
| BBIF_01069 | darkturquoise | Sugar kinase of the NBD/HSP70 family |
| BBIF_01070 | darkturquoise | copper homeostasis protein |
| BBIF_01071 | darkturquoise | Sugar kinase of the NBD/HSP70 family |
| BBIF_01072 | darkturquoise | fructokinase |
| BBIF_01073 | darkturquoise | Fucose permease |
| BBIF_01096 | darkturquoise | GTP-binding protein HflX |
| BBIF_01118 | darkturquoise | Predicted DNA-binding transcriptional regulator YafY |
| BBIF_01125 | darkturquoise | Glycosyl hydrolases family 32 N-terminal domain-containing protein |
| BBIF_01155 | darkturquoise | beta-galactosidase |
| BBIF_01170 | darkturquoise | hypothetical protein |
| BBIF_01183 | darkturquoise | hypothetical protein |
| BBIF_01184 | darkturquoise | anaerobic ribonucleoside-triphosphate reductase activating protein |
| BBIF_01190 | darkturquoise | regulatory protein |
| BBIF_01195 | darkturquoise | exopolyphosphatase / guanosine-5'-triphosphate |
| BBIF_01201 | darkturquoise | Subtilase family protein |
| BBIF_01202 | darkturquoise | aminopeptidase N |
| BBIF_01218 | darkturquoise | Lacto-N-biose phosphorylase |
| BBIF_01223 | darkturquoise | transcriptional regulator |
| BBIF_01224 | darkturquoise | #N/D |
| BBIF_01225 | darkturquoise | hypothetical protein |
| BBIF_01243 | darkturquoise | Calcineurin-like phosphoesterase |
| BBIF_01249 | darkturquoise | hypothetical protein |
| BBIF_01250 | darkturquoise | haloacid dehalogenase superfamily |
| BBIF_01269 | darkturquoise | Phage integrase family protein |
| BBIF_01273 | darkturquoise | Relaxase/Mobilisation nuclease domain-containing protein |
| BBIF_01276 | darkturquoise | Abortive infection bacteriophage resistance protein |
| BBIF_01277 | darkturquoise | type I restriction enzyme |
| BBIF_01280 | darkturquoise | hypothetical protein |
| BBIF_01281 | darkturquoise | DNA binding domain-containing protein |
| BBIF_01299 | darkturquoise | 23S rRNA (pseudouridine1915-N3)-methyltransferase |
| BBIF_01300 | darkturquoise | putative ABC transport system permease protein |
| BBIF_01301 | darkturquoise | putative ABC transport system ATP-binding protein |
| BBIF_01306 | darkturquoise | ketol-acid reductoisomerase |
| BBIF_01312 | darkturquoise | CrcB protein |
| BBIF_01313 | darkturquoise | CrcB protein |
| BBIF_01326 | darkturquoise | #N/D |
| BBIF_01334 | darkturquoise | Pimeloyl-ACP methyl ester carboxylesterase |
| BBIF_01335 | darkturquoise | Uncharacterized conserved protein YbjQ |
| BBIF_01336 | darkturquoise | DivIVA domain-containing protein |
| BBIF_01337 | darkturquoise | 8-oxo-dGTP diphosphatase |
| BBIF_01338 | darkturquoise | ATP-dependent helicase IRC3 |
| BBIF_01341 | darkturquoise | hypothetical protein |
| BBIF_01342 | darkturquoise | hypothetical protein |
| BBIF_01357 | darkturquoise | Uncharacterized conserved protein YlxW |
| BBIF_01358 | darkturquoise | Uncharacterised protein family (UPF0233) |
| BBIF_01359 | darkturquoise | N6-adenine-specific methylase |
| BBIF_01369 | darkturquoise | drug resistance transporter |
| BBIF_01370 | darkturquoise | hypothetical protein |
| BBIF_01375 | darkturquoise | Glycosyltransferase involved in cell wall bisynthesis |
| BBIF_01377 | darkturquoise | hypothetical protein |
| BBIF_01381 | darkturquoise | transposase |
| BBIF_01382 | darkturquoise | Transposase InsO and inactivated derivatives |
| BBIF_01385 | darkturquoise | NAD-dependent deacetylase |
| BBIF_01388 | darkturquoise | phospholipase/carboxylesterase |
| BBIF_01390 | darkturquoise | dCTP deaminase |
| BBIF_01391 | darkturquoise | alpha-galactosidase |
| BBIF_01396 | darkturquoise | hypothetical protein |
| BBIF_01397 | darkturquoise | sortase A |
| BBIF_01398 | darkturquoise | LPXTG-motif cell wall anchor domain-containing protein/fimbrial isopeptide formation D2 domain-containing protein |
| BBIF_01399 | darkturquoise | LPXTG-motif cell wall anchor domain-containing protein |
| BBIF_01405 | darkturquoise | #N/D |
| BBIF_01410 | darkturquoise | poly(A) polymerase |
| BBIF_01411 | darkturquoise | ADP-ribose pyrophosphatase YjhB |
| BBIF_01416 | darkturquoise | chromosome partitioning protein |
| BBIF_01417 | darkturquoise | 16S rRNA (guanine527-N7)-methyltransferase |
| BBIF_01420 | darkturquoise | large subunit ribosomal protein L34 |
| BBIF_01421 | darkturquoise | chromosomal replication initiator protein |
| BBIF_01428 | darkturquoise | alpha-N-arabinofuranosidase |
| BBIF_01431 | darkturquoise | transcriptional regulator |
| BBIF_01435 | darkturquoise | protein of unknown function (DUF4160) |
| BBIF_01440 | darkturquoise | DNA-binding transcriptional regulator |
| BBIF_01442 | darkturquoise | Ig-like domain (group 3) |
| BBIF_01443 | darkturquoise | #N/D |
| BBIF_01444 | darkturquoise | #N/D |
| BBIF_01445 | darkturquoise | Glycosyl hydrolase family 20 |
| BBIF_01446 | darkturquoise | transcriptional regulator |
| BBIF_01447 | darkturquoise | PTS system |
| BBIF_01463 | darkturquoise | hypothetical protein |
| BBIF_01466 | darkturquoise | Protein of unknown function (DUF3073) |
| BBIF_01473 | darkturquoise | transposase |
| BBIF_01474 | darkturquoise | hypothetical protein |
| BBIF_01475 | darkturquoise | Putative inner membrane protein (DUF1819) |
| BBIF_01502 | darkturquoise | protein of unknown function (DUF4193) |
| BBIF_01503 | darkturquoise | Protein of unknown function (DUF3071) |
| BBIF_01527 | darkturquoise | nicotinamide-nucleotide amidase |
| BBIF_01528 | darkturquoise | CDP-diacylglycerol--glycerol-3-phosphate 3-phosphatidyltransferase |
| BBIF_01538 | darkturquoise | DNA-binding response regulator |
| BBIF_01542 | darkturquoise | carbamoyl-phosphate synthase small subunit |
| BBIF_01545 | darkturquoise | hypothetical protein |
| BBIF_01555 | darkturquoise | Cadherin-like beta sandwich domain-containing protein |
| BBIF_01557 | darkturquoise | phosphoribosylaminoimidazole-succinocarboxamide synthase |
| BBIF_01558 | darkturquoise | PTS system |
| BBIF_01559 | darkturquoise | 6-phospho-beta-glucosidase |
| BBIF_01560 | darkturquoise | hypothetical protein |
| BBIF_01562 | darkturquoise | Putative ABC-transporter type IV |
| BBIF_01564 | darkturquoise | hypothetical protein |
| BBIF_01576 | darkturquoise | Glycosyl transferase family 2 |
| BBIF_01599 | darkturquoise | Acetyltransferase (GNAT) family protein |
| BBIF_01600 | darkturquoise | Uncharacterized membrane protein YccF |
| BBIF_01601 | darkturquoise | Methyltransferase domain-containing protein |
| BBIF_01602 | darkturquoise | hypothetical protein |
| BBIF_01604 | darkturquoise | Helix-turn-helix |
| BBIF_01614 | darkturquoise | serine/threonine protein kinase |
| BBIF_01628 | darkturquoise | phenylalanyl-tRNA synthetase |
| BBIF_01629 | darkturquoise | phenylalanyl-tRNA synthetase beta subunit |
| BBIF_01645 | darkturquoise | hypothetical protein |
| BBIF_01676 | darkturquoise | hypothetical protein |
| BBIF_01678 | darkturquoise | Head fiber protein |
| BBIF_01682 | darkturquoise | hypothetical protein |
| BBIF_01688 | darkturquoise | #N/D |
| BBIF_01690 | darkturquoise | hypothetical protein |
| BBIF_01697 | darkturquoise | hypothetical protein |
| BBIF_01701 | darkturquoise | Protein of unknwon function (DUF3310) |
| BBIF_01712 | darkturquoise | hypothetical protein |
| BBIF_01714 | darkturquoise | Helix-turn-helix. |
| BBIF_01736 | darkturquoise | aspartyl/glutamyl-tRNA(Asn/Gln) amidotransferase subunit C |
| BBIF_01737 | darkturquoise | ABC-2 type transport system ATP-binding protein |
| BBIF_01738 | darkturquoise | ABC-2 type transport system ATP-binding protein |
| BBIF_01744 | darkturquoise | ubiquinone biosynthesis protein |
| BBIF_01745 | darkturquoise | Protein of unknown function (DUF2680). |
| BBIF_01749 | darkturquoise | putative efflux protein |
| BBIF_01765 | darkturquoise | hypothetical protein |
| BBIF_01768 | darkturquoise | Protein of unknown function (DUF3000) |
| BBIF_01769 | darkturquoise | ribonuclease D |
| BBIF_01770 | darkturquoise | trigger factor |
| BBIF_01775 | darkturquoise | #N/D |
| BBIF_01776 | darkturquoise | #N/D |
| BBIF_01786 | darkturquoise | pyridoxine kinase |
| BBIF_01807 | darkturquoise | hypothetical protein |
| BBIF_01811 | darkturquoise | leader peptidase (prepilin peptidase) / N-methyltransferase |
| BBIF_01820 | darkturquoise | putative ABC transport system ATP-binding protein |
| BBIF_01823 | darkturquoise | DNA helicase-2 / ATP-dependent DNA helicase PcrA |
| BBIF_01838 | darkturquoise | hypothetical protein |
| BBIF_01839 | darkturquoise | hypothetical protein |
| BBIF_01843 | darkturquoise | hypothetical protein |
| BBIF_01849 | darkturquoise | #N/D |
| BBIF_01850 | darkturquoise | hypothetical protein |
| BBIF_01857 | darkturquoise | Acetyltransferase (GNAT) domain-containing protein |
| BBIF_01865 | darkturquoise | Putative DNA-binding domain-containing protein |
| BBIF_01866 | darkturquoise | dTDP-glucose 4 |
| BBIF_01868 | darkturquoise | Glycosyltransferase involved in cell wall bisynthesis |
| BBIF_01891 | darkturquoise | G/U mismatch-specific uracil-DNA glycosylase |
| BBIF_01895 | darkturquoise | Phage integrase family protein |
| BBIF_01896 | darkturquoise | Type I restriction modification DNA specificity domain-containing protein |
| BBIF_01897 | darkturquoise | protein of unknown function (DUF4357) |
| BBIF_01898 | darkturquoise | type I restriction enzyme M protein |
| BBIF_01899 | darkturquoise | hypothetical protein |

*** Shaded rows refer to genes associated with HMO consumption, according to Garrido et al., (2015).**

**Table S5. Genes in module Purple for B. *bifidum* SC555**.***

| **Locus Tag** | **Module** | **Annotation** |
| --- | --- | --- |
| BBIF_00013 | purple | hypothetical protein |
| BBIF_00150 | purple | transketolase |
| BBIF_00151 | purple | transaldolase |
| BBIF_00172 | purple | UDP-glucose 4-epimerase |
| BBIF_00173 | purple | UDPglucose--hexose-1-phosphate uridylyltransferase |
| BBIF_00174 | purple | N-acetylhexosamine 1-kinase |
| BBIF_00178 | purple | carbohydrate ABC transporter membrane protein 2 |
| BBIF_00179 | purple | multiple sugar transport system permease protein |
| BBIF_00180 | purple | multiple sugar transport system substrate-binding protein |
| BBIF_00265 | purple | hypothetical protein |
| BBIF_00266 | purple | hypothetical protein |
| BBIF_00271 | purple | argininosuccinate lyase |
| BBIF_00292 | purple | acetate kinase |
| BBIF_00318 | purple | SSU ribosomal protein S1P |
| BBIF_00356 | purple | DNA-directed RNA polymerase subunit beta' |
| BBIF_00394 | purple | hypothetical protein |
| BBIF_00425 | purple | acetolactate synthase |
| BBIF_00503 | purple | endo-alpha-N-acetylgalactosaminidase |
| BBIF_00527 | purple | PTS system |
| BBIF_00528 | purple | PTS system IIA component |
| BBIF_00662 | purple | hypothetical protein |
| BBIF_00753 | purple | polyribonucleotide nucleotidyltransferase |
| BBIF_00823 | purple | SSU ribosomal protein S10P |
| BBIF_00824 | purple | large subunit ribosomal protein L3 |
| BBIF_00825 | purple | large subunit ribosomal protein L4 |
| BBIF_00826 | purple | LSU ribosomal protein L23P |
| BBIF_00827 | purple | LSU ribosomal protein L2P |
| BBIF_00828 | purple | SSU ribosomal protein S19P |
| BBIF_00829 | purple | LSU ribosomal protein L22P |
| BBIF_00830 | purple | SSU ribosomal protein S3P |
| BBIF_00832 | purple | large subunit ribosomal protein L29 |
| BBIF_00834 | purple | LSU ribosomal protein L14P |
| BBIF_00835 | purple | large subunit ribosomal protein L24 |
| BBIF_00836 | purple | LSU ribosomal protein L5P |
| BBIF_00837 | purple | small subunit ribosomal protein S14 |
| BBIF_00838 | purple | SSU ribosomal protein S8P |
| BBIF_00839 | purple | large subunit ribosomal protein L6 |
| BBIF_00840 | purple | large subunit ribosomal protein L18 |
| BBIF_00841 | purple | small subunit ribosomal protein S5 |
| BBIF_00842 | purple | large subunit ribosomal protein L30 |
| BBIF_00844 | purple | protein translocase subunit secY/sec61 alpha |
| BBIF_00845 | purple | adenylate kinase |
| BBIF_00850 | purple | DNA-directed RNA polymerase subunit alpha |
| BBIF_00860 | purple | translation initiation factor IF-2 |
| BBIF_00869 | purple | hyaluronoglucosaminidase |
| BBIF_00915 | purple | para-aminobenzoate synthetase component 1 |
| BBIF_00963 | purple | Superfamily II RNA helicase |
| BBIF_01012 | purple | dihydroxyacid dehydratase |
| BBIF_01067 | purple | N-acetylglucosamine-6-phosphate deacetylase |
| BBIF_01068 | purple | glucosamine-6-phosphate deaminase |
| BBIF_01148 | purple | signal peptidase II |
| BBIF_01158 | purple | hydroxymethylpyrimidine synthase |
| BBIF_01178 | purple | Uncharacterised Sugar-binding Domain |
| BBIF_01179 | purple | hypothetical protein |
| BBIF_01203 | purple | ribonuclease J |
| BBIF_01231 | purple | LSU ribosomal protein L11P |
| BBIF_01253 | purple | hypothetical protein |
| BBIF_01261 | purple | alpha-L-fucosidase 2 |
| BBIF_01367 | purple | IstB-like ATP binding protein |
| BBIF_01419 | purple | YidC/Oxa1 family membrane protein insertase |
| BBIF_01426 | purple | DNA gyrase subunit A |
| BBIF_01438 | purple | hypothetical protein |
| BBIF_01448 | purple | PTS system |
| BBIF_01449 | purple | PTS system IIC component |
| BBIF_01457 | purple | thioredoxin reductase (NADPH) |
| BBIF_01458 | purple | hypothetical protein |
| BBIF_01476 | purple | protein of unknown function (DUF1788) |
| BBIF_01519 | purple | sigma 54 modulation protein /SSU ribosomal protein S30P |
| BBIF_01541 | purple | carbamoyl-phosphate synthase large subunit |
| BBIF_01543 | purple | NusB antitermination factor |
| BBIF_01622 | purple | AraC-like ligand binding domain-containing protein |
| BBIF_01632 | purple | glutamate N-acetyltransferase |
| BBIF_01633 | purple | N-acetylglutamate kinase |
| BBIF_01634 | purple | acetylornithine aminotransferase |
| BBIF_01637 | purple | argininosuccinate synthase |
| BBIF_01675 | purple | hypothetical protein |
| BBIF_01679 | purple | phage major capsid protein |
| BBIF_01766 | purple | formate C-acetyltransferase |
| BBIF_01767 | purple | pyruvate formate lyase activating enzyme |
| BBIF_01864 | purple | Transposase (or an inactivated derivative) |

*** Shaded rows refer to genes associated with HMO consumption according to Garrido et al., (2015).**

**Table S6. Genes in Green module for *B. longum* SC596*.**

| **Locus Tag** | **Module** | **Annotation** |
| --- | --- | --- |
| BLNG_00120 | Green | L-arabinose isomerase |
| BLNG_00123 | Green | putative ABC transport system permease protein |
| BLNG_00128 | Green | ATP-binding cassette |
| BLNG_00147 | Green | UDPglucose 6-dehydrogenase |
| BLNG_00161 | Green | carbohydrate ABC transporter membrane protein 1 |
| BLNG_00162 | Green | carbohydrate ABC transporter membrane protein 2 |
| BLNG_00163 | Green | 1,3-beta-galactosyl-N-acetylhexosamine phosphorylase |
| BLNG_00164 | Green | N-acetylhexosamine 1-kinase |
| BLNG_00165 | Green | UTP-hexose-1-phosphate uridylyltransferase |
| BLNG_00169 | Green | hypothetical protein |
| BLNG_00170 | Green | hypothetical protein |
| BLNG_00222 | Green | ABC transporter |
| BLNG_00238 | Green | D-xylose isomerase |
| BLNG_00245 | Green | Glycosyl hydrolases family 43 |
| BLNG_00246 | Green | Glycosyl hydrolases family 43 |
| BLNG_00252 | Green | xylulokinase |
| BLNG_00309 | Green | hypothetical protein |
| BLNG_00390 | Green | Helix-turn-helix domain-containing protein |
| BLNG_00393 | Green | DNA segregation ATPase FtsK/SpoIIIE |
| BLNG_00399 | Green | hypothetical protein |
| BLNG_00407 | Green | aconitase |
| BLNG_00408 | Green | cation-transporting ATPase E |
| BLNG_00459 | Green | Sugar kinase of the NBD/HSP70 family |
| BLNG_00460 | Green | Sugar kinase of the NBD/HSP70 family |
| BLNG_00535 | Green | hypothetical protein |
| BLNG_00536 | Green | Predicted DNA-binding transcriptional regulator YafY |
| BLNG_00717 | Green | putative membrane protein |
| BLNG_00718 | Green | hypothetical protein |
| BLNG_00726 | Green | Ig-like domain (group 2) |
| BLNG_00727 | Green | Glycosyl hydrolases family 43 |
| BLNG_00728 | Green | Beta-xylosidase |
| BLNG_00730 | Green | Ig-like domain (group 3) |
| BLNG_00739 | Green | F5/8 type C domain-containing protein |
| BLNG_00807 | Green | ATP-dependent Clp protease ATP-binding subunit ClpB |
| BLNG_00808 | Green | glutamyl-tRNA synthetase |
| BLNG_00850 | Green | Mg-chelatase subunit ChlD |
| BLNG_00851 | Green | LPXTG-motif cell wall anchor domain-containing protein |
| BLNG_00857 | Green | type 2 lantibiotic |
| BLNG_00860 | Green | type 2 lantibiotic biosynthesis protein LanM |
| BLNG_00861 | Green | ABC-type multidrug transport system |
| BLNG_00862 | Green | ABC-type bacteriocin/lantibiotic exporter |
| BLNG_00933 | Green | monosaccharide ABC transporter membrane protein |
| BLNG_00934 | Green | monosaccharide ABC transporter membrane protein |
| BLNG_00935 | Green | monosaccharide ABC transporter ATP-binding protein |
| BLNG_00936 | Green | monosaccharide ABC transporter substrate-binding protein |
| BLNG_00937 | Green | Lipoprotein LpqB beta-propeller domain-containing protein |
| BLNG_01059 | Green | long-chain acyl-CoA synthetase |
| BLNG_01076 | Green | WXG100 family type VII secretion target |
| BLNG_01097 | Green | proteasome accessory factor A |
| BLNG_01125 | Green | glycine cleavage system H protein |
| BLNG_01216 | Green | ATP synthase F1 subcomplex gamma subunit |
| BLNG_01217 | Green | F-type H+-transporting ATPase subunit beta |
| BLNG_01235 | Green | Small-conductance mechanosensitive channel |
| BLNG_01283 | Green | Predicted membrane protein (DUF2207) |
| BLNG_01345 | Green | carbohydrate ABC transporter ATP-binding protein |
| BLNG_01372 | Green | N-acetylglucosamine 6-phosphate deacetylase |
| BLNG_01373 | Green | oligo-1 |
| BLNG_01374 | Green | transcriptional regulator |
| BLNG_01414 | Green | glycogen operon protein |
| BLNG_01415 | Green | 2-desacetyl-2-hydroxyethyl bacteriochlorophyllide A dehydrogenase |
| BLNG_01416 | Green | polyol permease family |
| BLNG_01458 | Green | alpha-N-arabinofuranosidase |
| BLNG_01507 | Green | glucose-6-phosphate 1-dehydrogenase |
| BLNG_01509 | Green | 6-phosphogluconolactonase |
| BLNG_01517 | Green | putative ABC transport system permease protein |
| BLNG_01518 | Green | putative ABC transport system permease protein |
| BLNG_01526 | Green | endo-alpha-N-acetylgalactosaminidase |
| BLNG_01578 | Green | molecular chaperone DnaK |
| BLNG_01640 | Green | formate C-acetyltransferase |
| BLNG_01661 | Green | O-acetylhomoserine sulfhydrylase |
| BLNG_01665 | Green | Signal transduction histidine kinase |
| BLNG_01666 | Green | hypothetical protein |
| BLNG_01680 | Green | hypothetical protein |
| BLNG_01731 | Green | Iron-regulated ABC transporter ATPase subunit SufC |
| BLNG_01734 | Green | Metal-sulfur cluster biosynthetic enzyme |
| BLNG_02045 | Green | hypothetical protein |
| BLNG_02060 | Green | arabinoxylan arabinofuranohydrolase |
| BLNG_02061 | Green | 16S |
| BLNG_02070 | Green | energy-coupling factor transport system ATP-binding protein |
| BLNG_02071 | Green | energy-coupling factor transport system permease protein |
| BLNG_02141 | Green | choloylglycine hydrolase |

**Table S7. Genes in Darkgreen module for *B. longum* SC596.**

| **Locus taq** | **Module** | **Annotation** |
| --- | --- | --- |
| BLNG_00013 | darkgreen | Beta-galactosidase trimerisation domain-containing protein |
| BLNG_00025 | darkgreen | DNA polymerase I |
| BLNG_00041 | darkgreen | hypothetical protein |
| BLNG_00042 | darkgreen | hypothetical protein |
| BLNG_00043 | darkgreen | hypothetical protein |
| BLNG_00044 | darkgreen | hypothetical protein |
| BLNG_00069 | darkgreen | hypothetical protein |
| BLNG_00174 | darkgreen | MFS transporter |
| BLNG_00214 | darkgreen | Protein of unknown function (DUF3180) |
| BLNG_00215 | darkgreen | acyl-CoA thioesterase-2 |
| BLNG_00259 | darkgreen | amino acid/amide ABC transporter membrane protein 1 |
| BLNG_00260 | darkgreen | amino acid/amide ABC transporter membrane protein 2 |
| BLNG_00261 | darkgreen | amino acid/amide ABC transporter ATP-binding protein 1 |
| BLNG_00262 | darkgreen | amino acid/amide ABC transporter ATP-binding protein 2 |
| BLNG_00298 | darkgreen | CoA-substrate-specific enzyme activase |
| BLNG_00372 | darkgreen | DNA gyrase subunit B /DNA topoisomerase IV subunit B |
| BLNG_00376 | darkgreen | serine/threonine protein kinase |
| BLNG_00398 | darkgreen | EamA-like transporter family protein |
| BLNG_00409 | darkgreen | hypothetical protein |
| BLNG_00427 | darkgreen | L-aspartate oxidase |
| BLNG_00431 | darkgreen | condensin subunit ScpA |
| BLNG_00475 | darkgreen | alpha-mannosidase |
| BLNG_00476 | darkgreen | alpha-mannosidase |
| BLNG_00485 | darkgreen | UDP-N-acetylmuramoyl-tripeptide--D-alanyl-D-alanine ligase |
| BLNG_00496 | darkgreen | cobalt-zinc-cadmium efflux system protein |
| BLNG_00504 | darkgreen | 1-(5-phosphoribosyl)-5-[(5-phosphoribosylamino)methylideneamino] imidazole-4-carboxamide isomerase |
| BLNG_00505 | darkgreen | imidazole glycerol phosphate synthase subunit hisH |
| BLNG_00506 | darkgreen | hypothetical protein |
| BLNG_00507 | darkgreen | imidazoleglycerol-phosphate dehydratase |
| BLNG_00527 | darkgreen | LPXTG-motif cell wall anchor domain-containing protein/fimbrial isopeptide formation D2 domain-containing protein |
| BLNG_00531 | darkgreen | hypothetical protein |
| BLNG_00641 | darkgreen | 1-deoxy-D-xylulose-5-phosphate synthase |
| BLNG_00644 | darkgreen | 5-(carboxyamino)imidazole ribonucleotide synthase |
| BLNG_00691 | darkgreen | Histidine phosphatase superfamily (branch 2) |
| BLNG_00698 | darkgreen | DNA-(apurinic or apyrimidinic site) lyase /endonuclease III |
| BLNG_00701 | darkgreen | Putative Mn2+ efflux pump MntP |
| BLNG_00707 | darkgreen | fatty acid synthase |
| BLNG_00708 | darkgreen | Acetyl-CoA carboxylase |
| BLNG_00709 | darkgreen | acetyl-CoA/propionyl-CoA carboxylase |
| BLNG_00712 | darkgreen | hypothetical protein |
| BLNG_00775 | darkgreen | uncharacterized protein |
| BLNG_00776 | darkgreen | ABC-type lipoprotein export system |
| BLNG_00777 | darkgreen | MacB-like core domain-containing protein |
| BLNG_00778 | darkgreen | Protein of unknown function (DUF4012) |
| BLNG_00780 | darkgreen | hypothetical protein |
| BLNG_00892 | darkgreen | Forkhead associated (FHA) domain |
| BLNG_00905 | darkgreen | putative ABC transport system permease protein |
| BLNG_00926 | darkgreen | energy-coupling factor transport system ATP-binding protein |
| BLNG_00954 | darkgreen | 2' |
| BLNG_00975 | darkgreen | hypothetical protein |
| BLNG_01007 | darkgreen | 23S rRNA (adenine-N6)-dimethyltransferase |
| BLNG_01027 | darkgreen | protein of unknown function (DUF4913) |
| BLNG_01054 | darkgreen | Peptidase family M23 |
| BLNG_01154 | darkgreen | Major Facilitator Superfamily protein |
| BLNG_01162 | darkgreen | phosphoglucosamine mutase |
| BLNG_01163 | darkgreen | peptide deformylase |
| BLNG_01230 | darkgreen | putative spermidine/putrescine transport system substrate-binding protein |
| BLNG_01255 | darkgreen | multiple sugar transport system permease protein |
| BLNG_01256 | darkgreen | multiple sugar transport system permease protein |
| BLNG_01258 | darkgreen | L-fuconate dehydratase |
| BLNG_01259 | darkgreen | L-fucose dehydrogenase |
| BLNG_01260 | darkgreen | Predicted metal-dependent hydrolase |
| BLNG_01261 | darkgreen | 4-hydroxy-tetrahydrodipicolinate synthase |
| BLNG_01262 | darkgreen | L-fucose mutarotase |
| BLNG_01263 | darkgreen | alpha-L-fucosidase |
| BLNG_01264 | darkgreen | Glycosyl hydrolase family 65 |
| BLNG_01265 | darkgreen | 16S rRNA (guanine966-N2)-methyltransferase |
| BLNG_01272 | darkgreen | bleomycin hydrolase |
| BLNG_01286 | darkgreen | hypothetical protein |
| BLNG_01347 | darkgreen | ribonucleoside-diphosphate reductase class Ib beta subunit |
| BLNG_01348 | darkgreen | hypothetical protein |
| BLNG_01383 | darkgreen | Chaperone for flagella basal body P-ring formation |
| BLNG_01405 | darkgreen | multiple sugar transport system permease protein |
| BLNG_01406 | darkgreen | beta-glucosidase |
| BLNG_01465 | darkgreen | riboflavin kinase / FMN adenylyltransferase |
| BLNG_01537 | darkgreen | Putative flippase GtrA (transmembrane translocase of bactoprenol-linked glucose) |
| BLNG_01588 | darkgreen | ketol-acid reductoisomerase |
| BLNG_01590 | darkgreen | metabolite-proton symporter |
| BLNG_01609 | darkgreen | hypothetical protein |
| BLNG_01625 | darkgreen | Predicted ATPase |
| BLNG_01636 | darkgreen | D-methionine transport system permease protein |
| BLNG_01670 | darkgreen | Domain of unknown function DUF1829. |
| BLNG_01709 | darkgreen | ATP-dependent DNA helicase |
| BLNG_01719 | darkgreen | alanyl-tRNA synthetase |
| BLNG_01785 | darkgreen | zinc/manganese transport system permease protein |
| BLNG_01868 | darkgreen | beta-glucuronidase |
| BLNG_01870 | darkgreen | transferase hexapeptide (six repeat-containing protein) |
| BLNG_01873 | darkgreen | putative efflux protein |
| BLNG_01882 | darkgreen | Putative flippase GtrA (transmembrane translocase of bactoprenol-linked glucose) |
| BLNG_01883 | darkgreen | conserved hypothetical integral membrane protein |
| BLNG_01906 | darkgreen | Major Facilitator Superfamily protein |
| BLNG_01907 | darkgreen | hypothetical protein |
| BLNG_01919 | darkgreen | YggT family protein |
| BLNG_01945 | darkgreen | DivIVA domain-containing protein |
| BLNG_01964 | darkgreen | amino acid ABC transporter ATP-binding protein |
| BLNG_01965 | darkgreen | amino acid ABC transporter membrane protein 2 |
| BLNG_01981 | darkgreen | DNA replication protein DnaC |
| BLNG_01982 | darkgreen | Transposase |
| BLNG_01985 | darkgreen | Transposase (or an inactivated derivative) |
| BLNG_01996 | darkgreen | DNA replication and repair protein RecF |
| BLNG_02010 | darkgreen | Predicted amidohydrolase |
| BLNG_02015 | darkgreen | Glycosyl hydrolases related to GH101 family |
| BLNG_02016 | darkgreen | peptide/nickel transport system substrate-binding protein |
| BLNG_02139 | darkgreen | methionine synthase (B12-independent) |
| BLNG_02244 | darkgreen | Uncharacterized protein |

*** Shaded rows refer to genes associated with HMO consumption according to Garrido et al., (2015).**

**Table S8. Hub genes for each module in *B. longum subsp. infantis ATCC 15697***

| **Module** | **Locus Taq** | **Degree** | **Coding protein** |
| --- | --- | --- | --- |
| Blue | Blon_1271 | 45 | Hypothetical protein |
| Greenyellow | Blon_1037 | 25 | ABC Transporter permease |
| Cyan | Blon_0980 | 38 | Transposase mutator type |
| Purple | Blon_2437 | 48 | ABC transporter permease |
| Darkgrey | Blon_2195 | 24 | Rivoflavin Kinase/ FAD Synthetase |
| Lightcyan | Blon_1368 | 19 | Ribulose-phosphate-3-epimerase |
| Black | Blon_0825 | 43 | Imidazoleglycerol-phosphate dehydratase |
| Salmon | Blon_0146 | 10 | Putative acyl protein synthase/acyl-CoA reductase-like protein. |
| Midmightblue | Blon_0536  Blon_0990  Blon_2438  Blon_0818 | 3  3  3  3 | Putative Transciptional regulator  Transposase  ABC Transport protein  Conserved hypothetical protein pfam DUF4186 |
| Lightyellow | Blon_2414 | 6 | Extracellular solute-binding protein, family 1 |
| Darkred | Blon_0620  Blon_0607  Blon_1379 | 4  4  4 | Nucleotidyltransferase substrate binding protein  AMP-dependent synthetase and ligase  Conserved hypothetical protein |
| Royalblue | Blon_1555 | 15 | Hypothetical protein |
| Darkturquoise | Blon_0709  Blon_0710  Blon_0711 | 6  6  6 | ABC Transporter protein  Extracellular solute binding protein, family 3  Polar amino acid ABC transporter protein, inner membrane unit. |
| Darkgreen | Blon_0498 | 10 | Integrase, catalytic region. |
| Orange | Blon_0005 | 4 | Conserved hypothetical protein |
| Darkorange | Blon_1826  Blon_1568 | 5  5 | Hypothetical protein  Hypothetical protein |
| White | Blon_1817 | 6 | Phage protein Gp19/Gp15/Gp42 |
| Skyblue | Blon_0627 | 3 | Membrane protein |
| Saddlebrown | Blon_0949 | 10 | RecA protein |
| Steelblue | Blon_0943 | 4 | Filamentation induced by cAMP protein Fic |

**Table S9. Hub genes for each module in *Bifidum SC555***

| **Module** | **Locus Taq** | **Degree** | **Coding protein** |
| --- | --- | --- | --- |
| Cyan | BBIF_01407 | 56 | 16S rRNA (adenine1518-N6/adenine1519-N6)-dimethyltransferase) |
| DarkTurquoise | BBIF_01738 | 20 | ABC-2 Type transport system, ATP-binding protein |
| Black | BBIF_01747 | 76 | UDP-*N*-acetylmuramyl tripeptide synthase |
| Turquoise | BBIF_00702 | 61 | 16S rRNA (cytidine1402-2’-O)-methyltransferase |
| Brown | BBIF_01233 | 15 | TetR/AcrR family transcriptional regulator |
| Pink | BBIF_00964 | 28 | Polyphosphate: nucleotide phosphotransferase |
| Purple | BBIF_00840  BBIF_00527 | 21  21 | Large subunit ribosomal L18  PTS system |
| Darkgrey | BBIF_00921 | 12 | Nucleoside-diphosphate-sugar epimerase |
| Darkred | BBIF_00795 | 8 | Putative FmdB Regulatory Protein |
| Darkorange | BBIF_016016  BBIF_00538  BBIF_00474 | 7  7  7 | Glycosyltransferase involved in cell wall biosynthesis  Hypothetical protein  FtsX transport permease |

**Table S10. Hub genes for each module in *B. longum* SC596**

| **Module** | **Locus Taq** | **Degree** | **Coding protein** |
| --- | --- | --- | --- |
| Cyan | BLNG_00490 | 19 | MraZ protein |
| Darkorange | BLNG_02236 | 18 | Amino acid DMT transport membrane protein YddG family |
| Darkolivegreen | BLNG_01776 | 17 | ADP-pyrophosphatase |
| Orange | BLNG_01683 | 14 | Helix turn Helix protein |
| Turquoise | BLNG_00139 | 36 | Manganese transport protein |
| Darkgreen | BLNG_02244 | 11 | ATP-dependent Zn protease |
| Green | BLNG_00718 | 14 | Nitroreductase family protein |
| Magenta | BLNG_01958  BLNG_01309 | 21  21 | Regulator of protease activity  Nicotinate phosphoribosyltransferase |
| Greenyellow | BLNG_02106 | 29 | Hypothetical protein |
| Salmon | BLNG_01438 | 40 | LSU ribosomal protein L5P |
| Darkmagenta | BLNG_00479 | 15 | Cell division protein FtsQ |

## Supplementary Figures

**Supplementary Figure S1:** Normalized data of samples. A) *Bifidobacterium longum* subsp. *infantis* ATCC 15697, B) *Bifidobacterium bifidum* SC555, C) *Bifidobacterium longum* subsp *longum* SC596. Normalized samples were taken from NCBI Geo Datasets. For WGCNA analysis, log_10_ normalize read counts were used for this purpose.

**Supplementary Figure S2:** Sample clustering to detect outliers. A) *B. longum* subsp*. infantis* ATCC 15697, B) *B. bifidum* SC 555, c) *B. longum* subsp*. longum* SC596. RNA-seq datasets (GSE58773, GSE87697, GSE59053) were evaluated by sample clustering according to the Euclidean distance between different samples observed for each bacterium (Figure 1). No outliers were detected in the clusters; therefore, 56 samples were used to construct a hierarchical clustering tree (dendogram).

**Supplementary Figure S3:** Network topology for different soft-thresholding powers on each Bifidobacterium member. A) *Bifidobacterium longum* subsp*. infantis* ATCC 15697, B) *Bifidobacterium bifidum* SC 555, C) *Bifidobacterium longum* subsp*. longum* SC596. Numbers in the plots indicate the corresponding soft thresholding powers. The approximate scale-free topology can be attained at the soft-thresholding power of 12, 12, 16, respectively.

**Supplementary Figure S4:** Dendrogram of consensus module eigengenes obtained by WGCNA on the consensus correlation. The red line is the merging threshold, and groups of eigengenes below the threshold represent modules whose expressions profiles should be merged due to their similarity.
